# Methylation-directed glycosylation of chromatin factors represses retrotransposon promoters

**DOI:** 10.1073/pnas.1912074117

**Published:** 2020-06-10

**Authors:** Mathieu Boulard, Sofia Rucli, John R. Edwards, Timothy H. Bestor

**Affiliations:** ^a^Epigenetics and Neurobiology Unit, European Molecular Biology Laboratory (EMBL), 00015 Monterotondo, Italy;; ^b^Joint PhD degree program, European Molecular Biology Laboratory and Faculty of Biosciences, Heidelberg University, 69117 Heidelberg, Germany;; ^c^Center for Pharmacogenomics, Department of Medicine, Washington University School of Medicine, St. Louis, MO 63110;; ^d^Department of Genetics and Development, College of Physicians and Surgeons of Columbia University, New York, NY 10032

**Keywords:** DNA methylation, protein *O*-glycosylation, gene silencing

## Abstract

The mechanisms by which methylated mammalian promoters are transcriptionally silenced even in the presence of all of the factors required for their expression have long been a major unresolved issue in the field of epigenetics. Repression requires the assembly of a methylation-dependent silencing complex that contains the TRIM28 protein (also known as KAP1 and TIF1β), a scaffolding protein without intrinsic repressive or DNA-binding properties. The identity of the key effector within this complex that represses transcription is unknown. We developed a methylation-sensitized interaction screen which revealed that TRIM28 was complexed with *O-*linked β-*N*-acetylglucosamine transferase (OGT) only in cells that had normal genomic methylation patterns. OGT is the only glycosyltransferase that modifies cytoplasmic and nuclear protein by transfer of *N*-acetylglucosamine (*O*-GlcNAc) to serine and threonine hydroxyls. Whole-genome analysis showed that *O-*glycosylated proteins and TRIM28 were specifically bound to promoters of active retrotransposons and to imprinting control regions, the two major regulatory sequences controlled by DNA methylation. Furthermore, genome-wide loss of DNA methylation caused a loss of *O-*GlcNAc from multiple transcriptional repressor proteins associated with TRIM28. A newly developed Cas9-based editing method for targeted removal of *O-*GlcNAc was directed against retrotransposon promoters. Local chromatin de-GlcNAcylation specifically reactivated the expression of the targeted retrotransposon family without loss of DNA methylation. These data revealed that *O*-linked glycosylation of chromatin factors is essential for the transcriptional repression of methylated retrotransposons.

## Significance

Methylated mammalian promoters are transcriptionally silenced by nuclear factors, but the identity of these factors and the molecular mechanism of methylation-induced repression have long been elusive. We show here that methylated promoters recruit *O*-linked β-*N*-acetylglucosaminetransferase (OGT), which monoglycosylates multiple chromatin factors at serine and threonine hydroxyls. This modification both antagonizes protein phosphorylation at those hydroxyls and induces structural transitions in multiple chromatin factors that modify or enhance their repressive activities so as to consolidate the repressed state.

It has been known for many years that the methylation of mammalian promoters induces heritable transcriptional repression (1–3). Genome-wide demethylation reactivates expression of silenced retrotransposons (4) and causes the biallelic expression of imprinted genes (5), which are normally expressed from only the allele of maternal or paternal origin. After introduction into cells, artificially methylated Pol II-dependent promoters are actively transcribed for a brief period prior to heritable silencing (6, 7). This indicates that recruitment of methylation-dependent repressive factors rather than a direct effect of cytosine methylation on the transcriptional machinery is responsible for silencing.

Biochemical studies identified proteins that bind to methylated DNA in vitro and had the properties expected of methylation-dependent transcriptional repressors. However, ablation of the genes that encode MeCP2 and other methylation-dependent DNA-binding proteins singly or in combination did not reactivate methylated promoters in vivo (8). Ablation of methylated DNA-binding proteins produces phenotypes that are much less severe than the phenotypes caused by deletions of DNA methyltransferase genes (9).

The components of the methylation-dependent repressive complex and the actual mechanisms that repress transcription are not known. The repression of methylated retrotransposon promoters requires the TRIM28 protein (also known as KAP1 and TIF1β) (10), as does the methylation-dependent monoallelic expression of imprinted genes (11), but TRIM28 is a structural factor that does not bind to DNA and lacks repressor activity (12, 13). We developed a combined genetic and biochemical screen to identify factors that interact with TRIM28 in a methylation-dependent manner. The only such factor that was strongly enriched in this screen was *O-*linked β-*N*-acetylglucosamine transferase (OGT), the sole protein glycosyltransferase that is active in the nucleus and cytoplasm. OGT has important regulatory functions in multiple pathways (14), but had not previously been directly related to DNA methylation. Whole-genome analysis showed that TRIM28 and proteins modified by OGT colocalize at transposon promoters and at imprinting control regions. In the absence of DNA methylation, multiple proteins with key roles in gene silencing failed to undergo modification by OGT. Targeted protein deglycosylation by a novel editing method reactivated the transcription of methylated retrotransposon promoters. These data show that *O*-glycosylation is an essential component of the system that represses methylated promoters.

## Results

### Ablation of TRIM28 Phenocopies Mutations that Cause Genome-Wide Demethylation.

Homozygosity for a strongly hypomorphic allele of *Trim28* in mouse embryos does not cause appreciable demethylation of DNA (*SI Appendix*, Fig. S1 *A* and *B*) but phenocopies the reactivation of intracisternal A-type particles (IAP) retrotransposons induced by genome-wide demethylation (4), as had been previously reported for a null allele of *Trim28* (10). As in the case of reactivated IAP retrotransposons, biallelic expression of imprinted genes caused by the hypomorphic *Trim28* mutation (11) did not involve significant demethylation of imprinting control regions (*SI Appendix*, Fig. S2). These data identify TRIM28 as an essential mediator of methylation-dependent silencing of transposons and methylation-dependent monoallelic expression of imprinted genes. However, TRIM28 does not bind to DNA directly nor does it possess intrinsic repressive activity and cannot be the ultimate effector protein that represses methylated promoters (12, 13). Demethylation did not cause dissociation of TRIM28 from IAP retrotransposon sequences (*SI Appendix*, Fig. S3), which implicates an unknown factor in the repression of methylated promoters.

### Methylation-Dependent Association of OGT with the TRIM28 Complex.

**Fig. 1.**
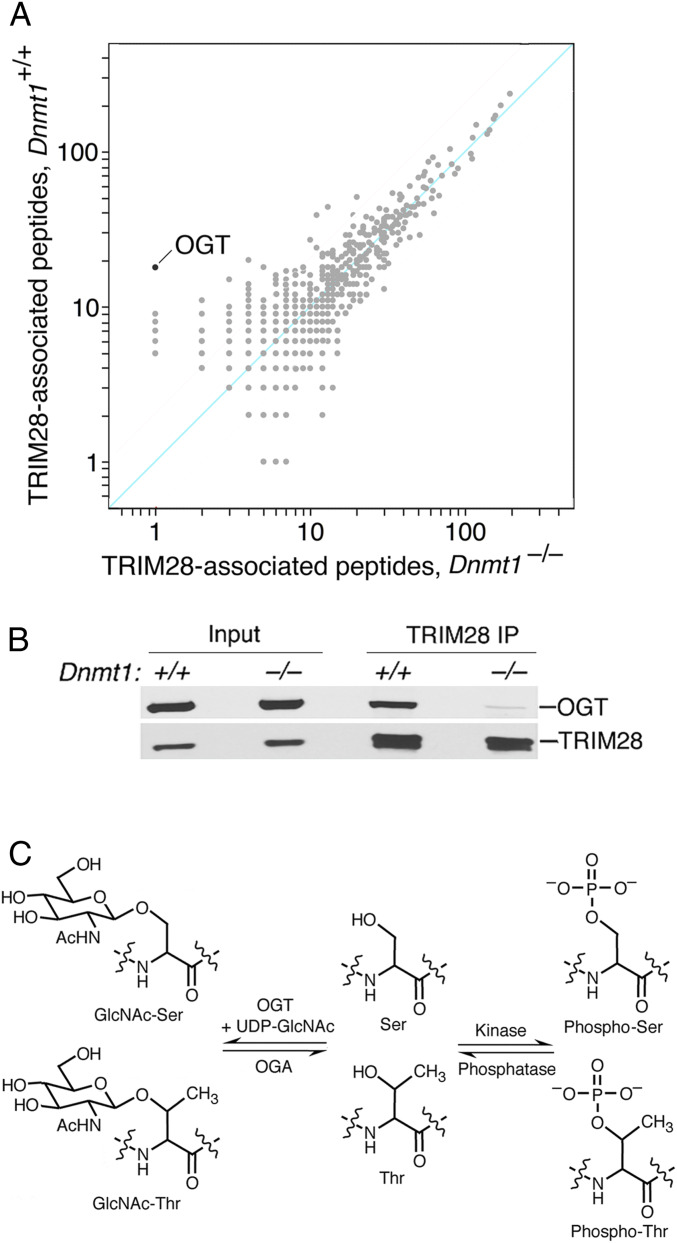
Identification of proteins in methylation-dependent TRIM28 complexes. (*A*) Native TRIM28 complexes were immunopurified from nuclear lysates of wild-type (*Dnmt1*^*+/+*^) and *Dnmt1*^*−/−*^ ES cells. Mass spectrometry analysis identified OGT as the protein most strongly dependent on DNA methylation for interaction with TRIM28. All proteins represented by three or more peptides are shown. A pseudocount of 1 was added to all peptide counts to allow for representation on the log plot shown. (*B*) Confirmation by immunoblot of the methylation-dependent association of OGT with TRIM28 complexes. (*C*) Reversible *O-*GlcNAcylation of serine and threonine by OGT and OGA at (*Left*) phosphorylation and (*Right*) dephosphorylation.

We developed a screen in which the composition of TRIM28 complexes in demethylated *Dnmt1*^*−/−*^ cells was compared to that of *Dnmt1*^*+/+*^ cells that had normal genomic methylation patterns. The only protein that showed a strong methylation-dependent association with TRIM28 was OGT (Fig. 1 *A* and *B* and *SI Appendix*, Table S1). OGT showed a methylation-dependent association with TRIM28 that was >2-fold greater than any other protein. This result was unexpected, as there had been no prior connection between DNA methylation and protein glycosylation (Fig. 1*C* and ref. 14).

### TRIM28 and *O-*GlcNAcylated Proteins Cooccupy Methylated Regulatory Sequences.

**Fig. 2.**
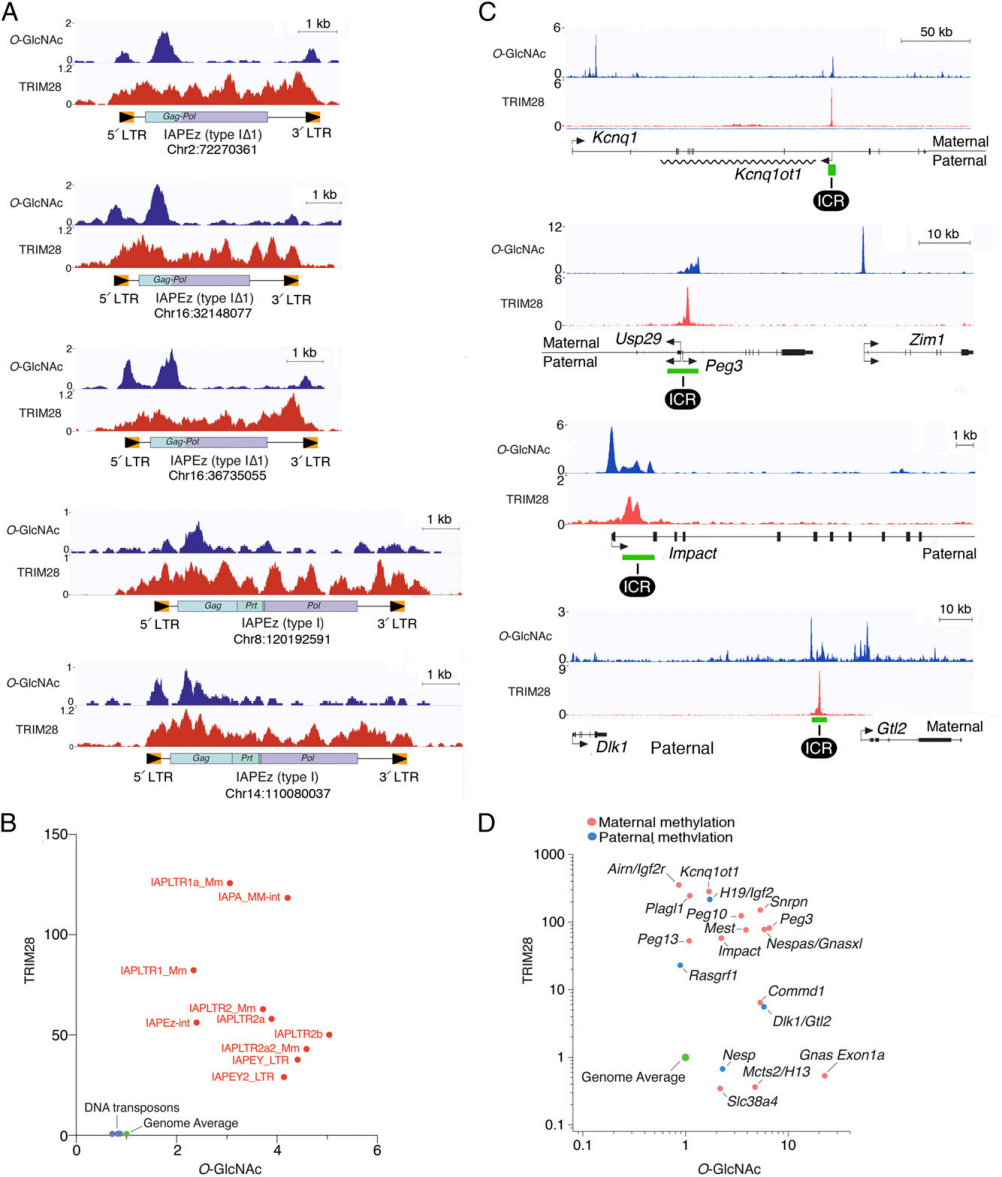
Methylated retrotransposon promoters and imprinting control regions are bound by TRIM28 and *O*-GlcNAcylated proteins. (*A*) Representative ChIP-seq data showing colocalization of *O*-GlcNAc and TRIM28 at IAPEz retrotransposons. The 5′ LTRs that contain the U3 promoter region and the Gag region are cooccupied by *O*-GlcNAcylated proteins and TRIM28. Type I is a 7-kb full-length element containing three ORFs (e.g., *Gag*, *Prt*, and *Pol*); type I∆1 has a deletion of *Prt* and part of *Gag*, resulting in a single ORF that encodes a novel GAG-POL fusion protein. IAPEz type I∆1 is the most highly reactivated subtype after removal of *O*-GlcNAc at LTRs (Fig. 4*E*). Similar strong reactivation of IAPEz I∆1 is observed in both *Dnmt1*^*−/−*^ and *Trim28*^*−/−*^ cells (*SI Appendix*, Fig. S1*B*). (*B*) TRIM28 and *O*-GlcNAc cooccupy all subtypes of LTRs. In contrast, DNA transposons, which are inactive in the mouse genome, are not occupied by TRIM28 or *O*-GlcNAcylated proteins. ChIP-seq signal of *O*-GlcNAc corresponds to the ratio of the mean of ChIP triplicates to the mean of input duplicates. ChIP-seq signal of TRIM28 is the ratio of the mean of ChIP duplicate to IgG control. Transposon subfamilies were categorized according to RepeatMasker annotation. (*C*) Genome browser views of ChIP-seq data showing cooccupancy of TRIM28 and *O*-GlcNAc at the indicated ICRs. The *Kcnq1ot1*, *Peg3*, and *Impact* ICRs are maternally methylated; *Dlk1/Gtl2* ICR (also known as IG-DMR) is paternally methylated. (*D*) TRIM28 and *O*-GlcNAcylated proteins are bound to all ICRs. ChIP-seq signals are the same as in *B*. ICR sequences were from ref. 46; TRIM28 ChIP-seq data were from ref. 45.

Whole-genome chromatin immunoprecipitation followed by DNA sequencing (ChIP-seq) using an antibody against *O-*GlcNAc revealed that long terminal repeats (LTRs) of IAP retrotransposons (the most actively proliferating retrotransposon in the mouse genome (15)) are densely occupied by *O-*GlcNAcylated proteins (Fig. 2*A*). Comparison of the ChIP-seq profiles of *O-*GlcNAc and TRIM28 showed that LTRs are cooccupied by TRIM28 and *O-*GlcNAcylated proteins (Fig. 2*B*). In contrast, DNA transposons that are incapable of transcription are not bound by *O-*GlcNAcylated proteins (Fig. 2*B*). While both LTRs have similar or identical sequences, the 5′ LTRs that contain the promoter are more densely *O-*GlcNAcylated (Fig. 2*A*). All tested subfamilies of IAP retrotransposons were enriched in both TRIM28 and *O-*GlcNAc (Fig. 2*B*).

Imprinting control regions (ICRs), which depend on DNA methylation for allele-specific expression (5), were inspected for occupancy by TRIM28 and *O-*GlcNAc. As shown in Fig. 2*C*, major ICRs recruited peaks of both TRIM28 and *O-*GlcNAc. All ICRs tested were enriched in either TRIM28 or *O-*GlcNAcylated proteins; the large majority was enriched in both (Fig. 2*D*).

### Genome Demethylation Causes Loss of *O-*GlcNAc from Proteins Complexed with TRIM28.

**Fig. 3.**
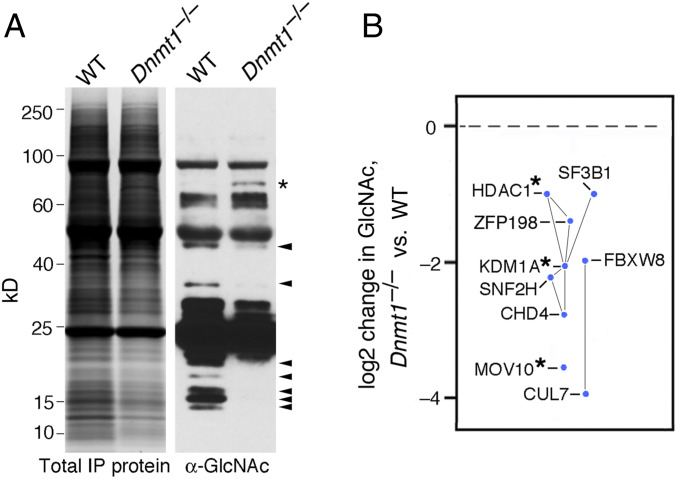
Methylation-dependent *O*-GlcNAcylation of protein associated with TRIM28. (*A*) Methylation-dependent *O*-GlcNAcylation of TRIM28-associated proteins. Nuclear extracts from wild-type (WT) and *Dnmt1*^*−/−*^ ES cells were subjected to native immunoprecipitation with anti-TRIM28 antibodies and analyzed by immunoblotting with anti-GlcNAc antibodies. Arrows at right indicate multiple proteins that are *O*-GlcNAcylated only in cells with methylated genomes. (*B*) Identification of TRIM28 interactors with methylation-dependent *O*-GlcNAcylation. Sequential immunoprecipitation with anti-TRIM28 and collection of *O*-GlcNAcylated proteins on WGA beads followed by analysis by mass spectrometry was used to identify proteins associated with TRIM28 in a DNA methylation-dependent manner. Each of the proteins shown was represented by >6 unique peptides and was depleted by >2-fold in *Dnmt1*^*−/−*^ cells. Connecting lines indicate reported interactions among the proteins identified. Proteins that have previously been implicated in the repression or restriction of retrotransposons or retroviruses (see text) are marked with asterisks. *x* axis is dimensionless.

Proteins subject to methylation-dependent *O-*GlcNAcylation were isolated from nuclear extracts of *Dnmt1*^*−/−*^ and *Dnmt1*^*+/+*^ ES cells by immunoprecipitation with antibodies to TRIM28 followed by collection by the GlcNAc-specific lectin Wheat Germ Agglutinin (WGA) and identification by mass spectrometry. As shown in Fig. 3*A*, genome demethylation in *Dnmt1*^*−/−*^ ES cells caused a loss of *O-*GlcNAc from multiple proteins complexed with TRIM28. The proteins showing the greatest degree of methylation-dependent *O-*GlcNAcylation are shown in Fig. 3*B* and *SI Appendix*, Table S2.

Multiple factors with known roles in transcriptional repression were found to undergo methylation-dependent *O-*GlcNAcylation. Many of these proteins had been previously reported to interact with each other directly or indirectly (Fig. 3*B*). TRIM28 assembles into a multiprotein complex containing HDAC1 and KDM1A (16), and ZFP198 stabilizes the repressive KDM1A-CoREST-HDAC1 complex on chromatin (17). The TRIM28-HDAC1-KDM1A complex has been reported to interact with CHD4 and SNF2H (18), and SF3B1 is a member of the SNF2H-WSTF silencing complex and a key mediator of Polycomb-dependent *Hox* gene repression (19), which is itself dependent on *O-*GlcNAcylation (20).

Each of the proteins subject to DNA methylation-dependent *O-*GlcNAcylation is involved in gene-silencing pathways. HDAC1 and KDM1A have been reported to repress retrotransposon transcription (16, 21), and MOV10 restricts LINE-1 retrotransposition (22). SNF2H and HDAC1 are required for the maintenance of silent chromatin (23). The CHD4-HDAC1 complex (also known as the NuRD complex) has nucleosome remodeling and histone deacetylase activity (24), and *O-*GlcNAcylation of HDAC1 stimulates its histone deacetylase activity and augments transcriptional silencing (25). Recessive mutations in the CUL7 gene, whose product is complexed with FBXW8, causes greatly reduced expression of the imprinted IGF2 gene and increased expression of H19 in human 3M syndrome type 1 without loss of allele-specific DNA methylation, which indicates that CUL7 is involved in the methylation-dependent imprinted expression of *H19* and *IGF2* (26, 27).

We confirmed that HDAC1, SNF2H, CHD4, ZFP198, and SF3B1 bear *O-*GlcNAc in ES cells and also found that 12 other proteins involved in transcriptional regulation were subject to *O-*GlcNAcylation (*SI Appendix*, Fig. S4). All DNA methyltransferases and all tested histones and histone variants were also *O-*GlcNAcylated. TRIM28 itself was the only silencing factor found to lack detectable *O-*GlcNAc. The number of factors subject to *O-*GlcNAcylation was larger than expected; GlcNAcylation has important roles in the regulation of transcription (28) but has received much less attention than posttranslational modifications such as acetylation, methylation, phosphorylation, or ubiquitylation.

### Targeted deGlcNAcylation Reactivates Methylated Transposable Elements.

**Fig. 4.**
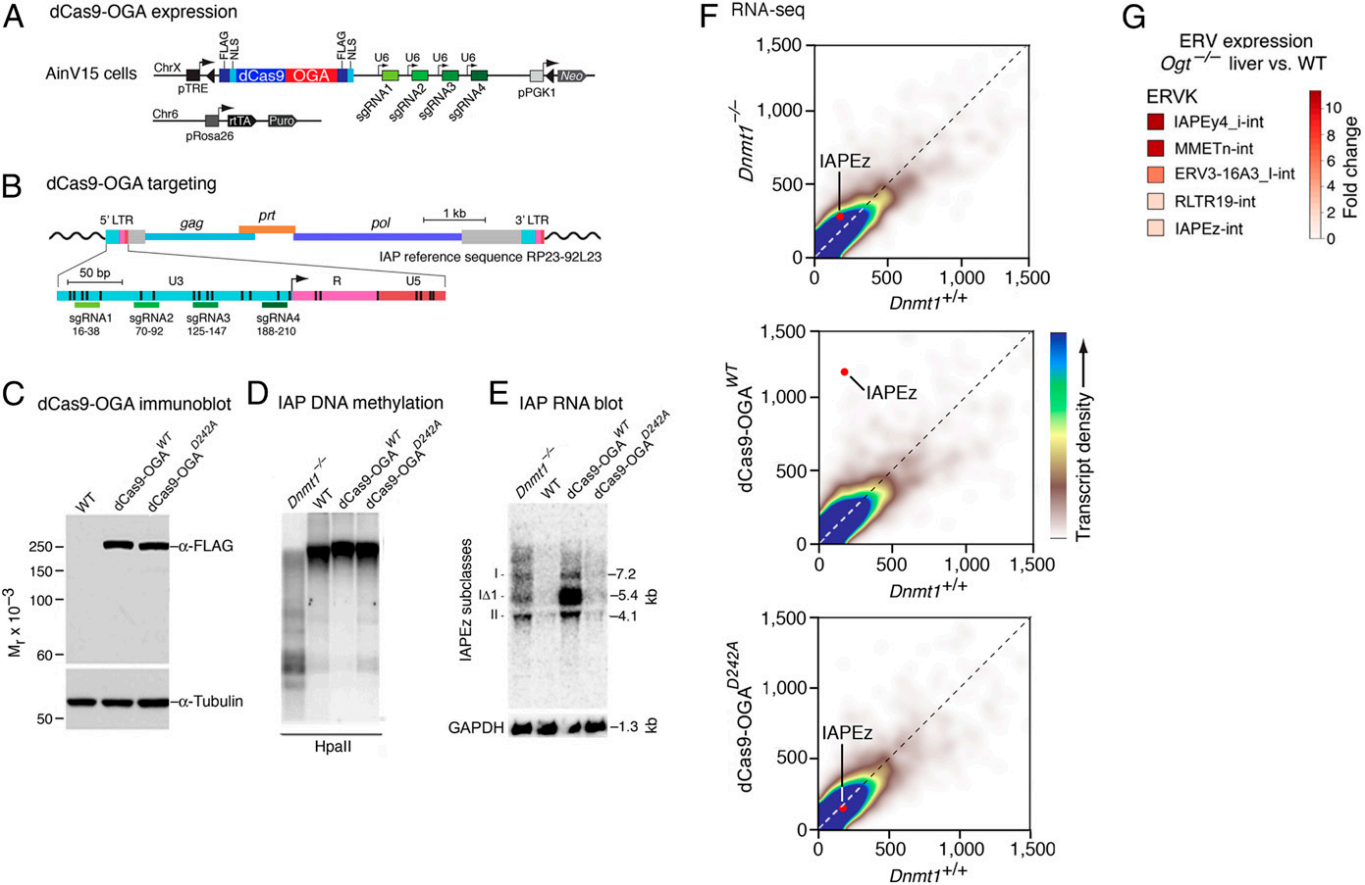
Reactivation of methylated promoters after targeted deGlcNAcylation. (*A*) An ES cell line was modified to express a dCas9-OGA fusion protein and four sgRNAs complementary to the U3 promoter region of IAP retrotransposons of the abundant IAPEz family. dCas9 from *Streptococcus pyogenes* had the RuvC endonuclease domain inactivated by a D10A substitution, and the HNH domain was inactivated by H839A and N863 substitutions. The coding region of the *Bacteroides* OGA gene (BtGH84) was synthesized and optimized for mammalian codon usage. The dCas9-OGA fusion protein was flanked by SV40 nuclear localization signals and by FLAG epitopes. The inducible cassette exchange system Plox-AinV15 was used to integrate the cDNA coding dCas9-OGA downstream of a Tet-On inducible promoter (pTRE). Tet-On regulation was provided via rtTA in *trans*. (*B*) sgRNA targets mapped to the IAP U3 promoter region. Dark vertical bars indicate position of CpG dinucleotides. (*C*) Immunoblot analysis of doxycycline-induced dCas9-OGA and dCas9-OGAD242A proteins shows equal expression of each stable fusion protein. (*D*) Expression of the dCas9-OGA and dCas9-OGA^D242A^ proteins as in *C* did not cause detectable demethylation of IAP proviral DNA, as determined by DNA blot hybridization after cleavage with the methylation-sensitive restriction endonuclease HpaII. (*E*) RNA blot hybridization shows that targeting of dCas9-OGA protein to IAP U3 regions induces strong reactivation of IAP transcription; the inactive dCas9-OGA^D242A^ mutant had no effect. (*F*) RNA-seq analysis confirms reactivation of IAP transcription by demethylation and by dCas9-OGA. RNA-seq read counts were >29,700,000 per sample. Axes are labeled in counts per million. (*G*) Reanalysis of RNA-seq data after liver-specific deletion of *Ogt* (32) shows strong reactivation of major ERVK transposons prior to death of the mutant cells by necrosis.

To test whether *O-*GlcNAcylation is required for methylation-dependent transcriptional repression, a new experimental approach was required, as genetic ablation of *Ogt* causes cell lethality (29). We therefore developed a new method to selectively deGlcNAcylate proteins bound to IAP retrotransposon promoters, which are Pol II-dependent promoters that are repressed by DNA methylation (4) but are not required for cell viability. We targeted the very well-characterized prokaryotic *O-*GlcNAc hydrolase (OGA BtGH84) from *Bacteroides thetaiotamicron* (30) to LTRs of endogenous IAP retrotransposons. A Cas9 expression vector was produced in which both Cas9 endonuclease domains had been inactivated by point mutations to produce a catalytically dead Cas9 (dCas9) that retained single guide RNA (sgRNA)-dependent DNA binding. An embryonic stem (ES) cell line was engineered to conditionally express a chimeric protein consisting of *B. thetaiotamicron* OGA fused to dCas9, together with four sgRNAs directed against the U3 promoter region of IAP retrotransposons (Fig. 4 *A* and *B*). The same fusion protein that contained a D242A mutant form of OGA that is unable to bind or hydrolyze *O-*GlcNAc (30) served as a control. As shown in Fig. 4*C*, both the dCas9-OGA and dCas9-OGA^D242A^ fusion proteins were stable and expressed at very similar levels.

The dCas9-OGA or dCas9-OGA^D242A^ fusion protein did not demethylate IAP proviral DNA (Fig. 4*D*), but the dCas9-OGA fusion protein induced a dramatic reactivation of IAP transcription (Fig. 4*E*). This strong release from silencing was specific to the subclass of IAP elements targeted (IAPEz) as other types of LTR transposons and non-LTR transposons remained repressed (*SI Appendix*, Fig. S5). The inactive dCas9-OGA^D242A^ fusion protein had no detectable effect, which indicates that reactivation was the result of deGlcNAcylation and not an effect of the binding of the dCas9-OGA-sgRNA complex. The RNA blot data were confirmed and quantitated by the RNA-seq data shown in Fig. 4*F*. The level of derepression was greater than that caused by demethylation, which may reflect the existence of both methylation-dependent (4) and methylation-independent mechanisms (31) of IAP repression. The data indicate that *O-*GlcNAcylation is required for both mechanisms of repression. However, the fact that methylated IAP retrotransposon promoters was reanimated by targeting the dCas9-OGA fusion protein to IAP promoters provides strong evidence that *O-*glycosylation mediates transcriptional repression.

Other direct evidence for a role of protein *O-*glycosylation in the silencing of retrotransposon comes from studies of a liver-specific deletion of *Ogt* in mice (32). We reanalyzed the RNA-seq data from this study for transposon reactivation. As shown in Fig. 4*G*, robust reanimation of multiple LTR transposons was apparent in deGlcNAcylated *Ogt*^−/−^ liver tissue prior to necrotic cell death. This result shows that genome-wide deGlcNAcylation reactivates multiple classes of methylated retrotransposons, whereas targeted deGlcNAcylation reactivates only the selected retrotransposon family.

## Discussion

While many glycosyltransferases modify secreted proteins and the extracellular domains of membrane proteins, OGT is the only glycosyltransferase that modifies nuclear and cytosolic proteins, and *O-*GlcNAcylation is the only form of glycosylation that is known to be highly dynamic and reversible (14). *O-*GlcNAcylation antagonizes phosphorylation of Ser and Thr, and while phosphorylation adds a strong anion that rearranges salt bridges (33), *O-*GlcNAcylation of the same residues introduces a cluster of hydrogen bond donors and acceptors that induce very different structural transitions in target proteins (Fig. 1*C*). Many repressive factors associated with TRIM28 complexes are subject to methylation-directed *O-*GlcNAcylation, which indicates that repression of methylated promoters is likely to be the result of *O-*GlcNAcylation of multiple chromatin factors.

There is abundant evidence for an important regulatory role of *O-*GlcNAcylation in gene expression, but no prior association with DNA methylation. *O-*GlcNAcylation is involved in many regulatory pathways; these include control of the interaction of YY1 with Rb1, which prevents YY1 from activating transcription (34), and STAT5 (35) and the pluripotency factor OCT4 (36) that are only active when *O-*GlcNAcylated. It is also of great interest that *O*-GlcNAcylation of the C-terminal domain (CTD) of the large subunit of RNA Pol II inhibits phosphorylation of the CTD and transcriptional elongation (37, 38). It is particularly intriguing that all Polycomb-mediated gene repression in *Drosophila* is dependent on the single *Ogt* gene (*super sex combs* or *sxc*) in the fly genome, even though Polycomb factors are bound to their normal sites in the *sxc* mutant (20).

The targeting of the repressive complex that contains TRIM28 and OGT to methylated promoters and imprinting control regions is likely to involve the very large and rapidly evolving group of KRAB-Zinc finger proteins that are restricted to tetrapod vertebrates and are especially numerous and diverse in mammals (39). We propose a model under which a class of methylation-independent KRAB-Zinc finger proteins nucleate TRIM28 complexes that lack OGT while methylation-dependent KRAB-Zinc finger proteins recruit TRIM28 and activate OGT. Cheng and colleagues estimate that ∼200 of >300 human KRAB-Zinc finger proteins are likely to display methylation-dependent binding to DNA (40). As shown in *SI Appendix*, Fig. S6 and *SI Appendix*, Table S3, many Zinc finger proteins are complexed with TRIM28. The most highly enriched KRAB-Zinc finger protein in TRIM28 complexes is Zfp568, which is required solely for the methylation-dependent imprinted expression of the *Igf2* gene (41). The data presented here support a model under which methylated regulatory sequences are bound in a sequence- and methylation-dependent manner by one or more of the many KRAB-Zinc finger proteins; this nucleates a methylation-specific complex of proteins that includes TRIM28 and OGT (*SI Appendix*, Fig. S7). We propose that subsequent *O-*GlcNAcylation induces structural transitions in multiple chromatin factors that modify or enhance their repressive activities to impose transcriptional repression on methylated promoters and to mediate monoallelic expression of imprinted genes.

## Materials and Methods

### ES Cells.

The ES cell line homozygous for a null allele of *Dnmt1* (*Dnmt1*^*−/−*^) was described previously (42). ES cells were cultured on gelatin-coated plates under standard conditions (DMEM, 2 mM Glutamax, 15% ES grade FBS, 2 mM L glutamine, MEM nonessential amino acids, 100 IU/mL penicillin, 100 μg/mL streptomycin, 0.12 mM 2-mercaptoethanol and leukemia inhibitory factor).

### Nuclear Extract Preparation.

ES cells were harvested at 80% confluency and resuspended in hypotonic lysis buffer (10 mM Hepes pH 7.65, 10 mM KCl, 1 mM MgCl2, 0.5 mM DTT, and complete protease inhibitors [Roche]) and incubated for 15 min on ice. Cells were treated with a Dounce homogenizer (25 strokes with tight pestle). Nuclei were recovered by centrifugation (10 min at 300 g at 4 °C), washed twice in buffer A (10 mM Hepes pH 7.65, 1 mM MgCl2, 0.5 mM DTT, 250 mM Sucrose, and complete protease inhibitors [Roche]), centrifuged (2,800 g for 10 min at 4 °C), and resuspended in buffer B (20 mM Hepes pH 7.65, 25% glycerol, 250 mM NaCl, 5 mM MgCl2, 0.2mM EDTA, 0.005% Nonidet P-40, 0.5 mM DTT, and complete protease inhibitors [Roche]). NaCl concentration was increased to 300 mM, and extraction of the soluble protein complexes was allowed to proceed under gentle agitation for 3 h at 4 °C. Nuclei were pelleted by centrifugation (3,000 g for 10 min at 4 °C), and the supernatant was collected as the nuclear soluble extract. Protein concentration was measured by bicinchoninic acid assay.

### Proteomic Screen for Methylation-Dependent TRIM28 Associated Proteins.

Ten micrograms of anti-TRIM28 monoclonal antibody (MAB3662, EMD Millipore) bound to 50 μL Dynabeads Protein G magnetic beads (Thermo Fisher Scientific) was incubated with 8 mg of ES cell nuclear soluble extract for 14–16 h at 4 °C. Bound material was eluted by incubating the beads at 95 °C for 5 min in a buffer containing 10 mM Hepes pH 7.65, 0.1% sodium dodecyl sulfate (SDS), 1% Nonidet P-40, 1 mM DTT, 300 mM NaCl. Complexes were resolved by SDS/PAGE, stained by SYPRO Ruby (Thermo Fisher Scientific), and identified by mass spectrometry at the Taplin Biological Mass Spectrometry Facility (Harvard Medical School, Boston, MA).

### ChIP-seq.

Chromatin immunoprecipitation was carried out on formaldehyde cross-linked chromatin. One hundred million ES cells were fixed for 10 min at room temperature with 1.1% formaldehyde and quenched with 125 mM glycine. Soluble chromatin was sheared by sonication to an average size of 250 bp using a Covaris S220 Sonicator with peak power 150, duty factor 25, cycles/burst 200. Immunoprecipitation was carried out overnight at 4 °C with 3 μg of monoclonal antibodies anti-*O*-GlcNAc (Thermo Fisher Scientific, MA1-076) bound to 10 μL Dynabeads conjugated with protein G (Life Technologies). Beads were washed and chromatin eluted as described previously (43). Immunoprecipitated DNA and input DNA were submitted to library preparation using the NEBNext Ultra II DNA Library Prep Kit for Illumina (New England Biolabs) following the manufacturer’s instructions and amplified for 15 cycles. The samples were sequenced in single-end mode on the Illumina NextSEq 500 platform at the European Molecular Biology Laboratory’s (EMBL) Genomics Core Facility.

### ChIP-seq Data Analysis.

ChIP-seq reads were mapped to the mouse genome (mm10) using bowtie2 (v2.2.2) and default parameters. Duplicate reads were removed using samtools rmdups (v1.3.1). The Macs2 (v2.0.10) callpeaks module was used to call peaks using -g 1.87e9, –SPMR, and -B flags and using the input as background (44). TRIM28 ChIP-seq reads were downloaded from GEO GSE59189 (45) and processed similarly. The coordinates of ICRs are described in ref. 46.

### Lectin-Based Purification of *O*-GlcNAcylated Proteins.

*O-*GlcNAcylated proteins were isolated with WGA conjugated to magnetic beads (47). *O-*GlcNAcylated proteins were isolated either from fractionated nuclei or from isolated TRIM28 complexes. The *O-*GlcNAase inhibitor PUGNAc (Tocris Biosciences) was added at 2 mM in hypotonic lysis buffer, buffer A and B in order to preserve physiological *O-*GlcNAc levels during the cellular fractionation procedure. Nuclei were lysed with 1% SDS, cleared of nucleic acids by treatment with Universal Nuclease (Pierce), and denatured by heating to 100 °C for 2 min in 1% SDS. Denatured proteins were incubated for 2 h at 4 °C with 200 μL of Dynabeads Streptavidin C1 (Thermo Fisher Scientific) bound to 200 μg of biotin-conjugate wheat germ agglutinin (Sigma). Beads were washed six times with 20 mM Hepes pH 7.65, 250 mM NaCl, 5 mM CaCl2, 1 mM MgCl2, 0.2% Nonidet P-40. GlcNAcylated proteins were eluted from the beads at 95 °C. The specificity of binding was controlled by competitive inhibition with 0.75 M *N*-acetylglucosamine.

### Coexpression of dCas9-OGA and sgRNA Targeted to IAP U3 Regions in ES Cells.

The chimeric protein dCas9-OGA was coexpressed with four sgRNA specific to the U3 region of the IAP retrotransposon in the tetracycline-inducible (Tet-On) gene expression system PLox-AinV15, which is designed to insert a circular Plox plasmid by cre/lox recombination into a recombinant doxycycline-inducible locus. The AinV15 cell line carries the reverse tetracycline transactivator (rtTA) integrated into the ubiquitously expressed ROSA26 locus (48). The complementary DNA (cDNA) encoding the dCas9-OGA fusion protein as well as four human U6 promoters driving expression of the sgRNAs were cloned into the P2lox vector (Adgene #34635). The mammalian codon-optimized enzymatically inactive Cas9 from *Streptococcus pyogenes* (dCas9 which bears the substitutions D10A, H839A, H840A, and N863A) fused to an N-terminal SV40 nuclear localization signal sequence, and a FLAG tag epitope was amplified by polymerase chain reaction (PCR). The mammalian codon-optimized OGA (30) from *Bacteroides* thetaiotaomicron GH84 (UniProtKB - Q89ZI2) fused to a C-terminal SV40 nuclear localization sequence and a FLAG tag epitope was synthesized using IDT gBlocks gene fragments (Integrated DNA Technologies). The DNA fragments encoding dCas9 and OGA were ligated into the SalI and the NotI sites of the P2lox vector using Gibson cloning (NEBuilder HiFi DNA Assembly Cloning Kit, NEB). The sgRNAs homologous to the IAP LTRs (Fig. 4*B*) were cloned between the BbsI sites of the px330 plasmid (Adgene #42230) to permit PCR amplification of the DNA sequences that contain the U6 promoter, the sgRNA, and the tracrRNA. The four DNA fragments containing U6 promoter and sgRNA were assembled together and cloned into the BsrGI site of the P2Lox plasmid via Gibson assembly (NEBuilder HiFi DNA Assembly Cloning Kit, NEB). The sequences of the sgRNAs are provided in *SI Appendix*, Table S4. The D242A mutation was previously shown to abolish OGA enzymatic activity and its binding to GlcNAc (30) and was generated by site-directed mutagenesis (Agilent Technologies).

Three million AinV15 ES cells were nucleofected with 10 μg of P2Lox plasmid containing the dCas9-OGA cDNA and four U6 promoter-driven sgRNAs and 10 μg of a plasmid-expressing Cre recombisase (Adgene #11543). Recombinant cells were selected by treatment with 350 μg/mL G418 and genotyped for proper integration by PCR as previously described (48). Expression of the dCas9-OGA transgene was induced by addition of 1 μg/mL of doxycycline (Sigma). After 48 h of induction, cells were harvested, and RNA, proteins, and genomic DNA were extracted for analyses.

### RNA Blot Hybridization.

Total RNA was isolated using TRIzol reagent (Thermo Fisher Scientific) from a pool of six embryos of same genotype dissected at embryonic day E8.5 or from 1 × 10^6^ ES cells. RNA was cleared of potential contaminating genomic DNA by two rounds of digestion with DNase (Turbo DNase, Ambion) and quantified using Qubit Fluorometric Quantitation (Thermo Fisher Scientific). Ten micrograms of total RNA was denatured and subjected to electrophoresis in a 1% agarose gel containing 1.9% formaldehyde prior to transfer to a nitrocellulose membrane. After ultraviolet cross-linking, the membrane was hybridized with a radiolabeled IAP probe as described (49). The *Gapdh* probe was cloned from cDNA using the primers described in *SI Appendix*, Table S4.

### RNA-seq.

Total RNA was extracted, and traces of contaminating genomic DNA were eliminated by two successive treatments with DNase (Turbo DNase, Ambion). The integrity of the RNA was verified using the Bioanalyzer RNA 2100 Nano Assay (Agilent Technologies). RNA-seq libraries were prepared with the TruSeq Stranded mRNA LT (Illumina), and massive parallel sequencing was performed in single-end reads using an Illumina HiSeq 4000 and Next-seq instruments. We obtained 38,676,845, 40,766,737, and 36,497,140 reads for three replicates of *Dnmt1*^*−/−*^ ES cells; and 40,282,098, 50,062,039, and 36,948,128 reads for three replicates of wild-type ES cells. Further, 38,798,921 and 38,840,713 reads were obtained for dCas9-OGA^WT^-expressing cells and dCa9-OGA^D242A^-expressing ES cells, respectively.

For IAPEz expression analysis, reads were mapped to the mouse reference genome (mm10) using bowtie2 (v2.2.2; ref. 50) and default parameters except for -D 10000 -R 10000. After filtering out reads that mapped to ribosomal RNA (rRNA) and messenger RNA (Ensembl v87) sequences, reads were overlapped with repeat annotations from the RepeatMasker track from the University of California Santa Cruz genome browser using featureCounts (v1.5.0) (50). Reads for individual repeat element families (e.g., IAPEz) were normalized to FPKM (fragments per 1000 bp per million reads). FPKM values from IAPEz were then background-adjusted using the FPKM value from all DNA transposons and then rescaled back to cpm (counts per million). For transcript analysis, reads were mapped to the mouse reference genome (mm10) using HISAT2 (v2.1.0) provided with known splice sites using Ensembl v87 and otherwise default parameters (51). After removal of rRNA sequences, alignment files were overlapped with gene annotations using featureCounts (v1.5.0; ref. 52) and Ensembl v87. Expression counts were normalized to cpm, and log2 fold change values were calculated using DESeq2.

### Data Availability.

The RNA-seq and ChIP-seq data reported in this study are available in the Gene Expression Omnibus (GEO) database (accession no. GSE93539).

## Supplementary Material

Supplementary File
