# Supplementary material for: Methylation-directed glycosylation of chromatin factors represses retrotransposon promoters
Source: Proc Natl Acad Sci U S A. 2020 Jun 10;117(25):14292–8. doi: 10.1073/pnas.1912074117 (PMC7322000; doi:10.1073/pnas.1912074117)
Supplement: Supplementary File [file pnas.1912074117.sapp.pdf]

# Supporting Information

## *Methylation-Directed O-Glycosylation of Chromatin Factors Represses Retrotransposon Promoters*

Mathieu Boulard[a,1], Sofia Rucli[a,b], John R. Edwards[c,1], and Timothy H. Bestor[d,1]

[a] European Molecular Biology Laboratory Rome, Via Ramarini 32, 00015 Monterotondo, Italy

[b] Joint PhD degree program, European Molecular Biology Laboratory and Heidelberg University, Faculty of Biosciences, Heidelberg, Germany

[c] Center for Pharmacogenomics, Department of Medicine, Washington University School of Medicine, 660 South Euclid Ave., St. Louis MO 63110

[d] Department of Genetics and Development, College of Physicians and Surgeons of Columbia University, 701 W. 168th St., New York NY 10032

[1] To whom correspondence should be addressed. Email: matthieu.boulard@embl.it, jredwards@wustl.edu, thb12@cumc.columbia.edu

This file includes:

**Supplementary Table S1**: Mass spectrometric identification of proteins co-IP with TRIM28.

**Supplementary Table S2**: O-GlcNAcylated proteins associated with TRIM28.

**Supplementary Table S3**: KRAB-C2H2-Zinc fingers TFs associated with TRIM28.

**Supplementary Table S4**: Reagents and Sequences.

**Supplementary Figure S1**: The protein TRIM28 is necessary to repress transcription of methylated retrotransposons.

**Supplementary Figure S2**: Loss of imprinted expression in Trim28C/C embryos occurs despite normal DNA methylation at differentialy methylated regions (DMRs).

**Supplementary Figure S3**: Demethylation does not cause dissociation of TRIM28 from IAP retrotransposon sequences.

**Supplementary Figure S4**: O-GlcNAcylation of proteins associated with TRIM28.

**Supplementary Figure S5**: Specific reactivation of IAPEz transposons upon targeting of dCas9-OGA to their LTRs.

**Supplementary Figure S6**: Predominance of proteins with KRAB and Zinc finger domains among the DNA binding proteins found to Co-IP with TRIM28 by mass spectrometry.

**Supplementary Figure S7**: Model.

**Supplementary Methods**

**Supplementray References**

www.pnas.org/cgi/doi/10.1073/pnas.1912074117

# SUPPLEMENTARY TABLES

## Table S1: Mass spectrometric identification of proteins co-IP with TRIM28 (native IP, ES cells, Anti-body MAB3662)

| WT | | | | | Dnmt1-null | | | | |
|---|---|---|---|---|---|---|---|---|---|
| Uniq | Tota | reference | Gene Syn | AVG | Uniq | Tota | reference | Gene Syn | AVG |
| 100 | 170 | PRP8_MOUSE | Prpf8 | 3.23 | 86 | 156 | PRP8_MOUSE | Prpf8 | 3.26 |
| 96 | 198 | Q6P4T2_MOUSE | Snrnp200 | 3.54 | 86 | 169 | Q6P4T2_MOUSE | Snrnp200 | 3.46 |
| 92 | 122 | E9Q7G0_MOUSE | Numa1 | 3.84 | 80 | 111 | E9Q7G0_MOUSE | Numa1 | 3.68 |
| 82 | 148 | RIF1_MOUSE | Rif1 | 3.67 | 69 | 117 | RIF1_MOUSE | Rif1 | 3.54 |
| 64 | 129 | TOP2A_MOUSE | Top2a | 3.42 | 67 | 138 | TOP2A_MOUSE | Top2a | 3.24 |
| 60 | 85 | KI20B_MOUSE | Kif20b | 3.71 | 46 | 57 | KI20B_MOUSE | Kif20b | 3.77 |
| 57 | 96 | RPB1_MOUSE | Polr2a | 3.45 | 61 | 108 | RPB1_MOUSE | Polr2a | 3.42 |
| 52 | 91 | MBB1A_MOUSE | Mybbp1a | 3.43 | 38 | 61 | MBB1A_MOUSE | Mybbp1a | 3.46 |
| 51 | 69 | CHD4_MOUSE | Chd4 | 3.42 | 46 | 53 | CHD4_MOUSE | Chd4 | 3.47 |
| 49 | 77 | SPT6H_MOUSE | Supt6h | 3.66 | 49 | 89 | SPT6H_MOUSE | Supt6h | 3.62 |
| 47 | 162 | DHX9_MOUSE | Dhx9 | 3.43 | 52 | 152 | DHX9_MOUSE | Dhx9 | 3.28 |
| 44 | 82 | SF3B1_MOUSE | Sf3b1 | 3.55 | 41 | 81 | SF3B1_MOUSE | Sf3b1 | 3.48 |
| 42 | 89 | SF3B3_MOUSE | Sf3b3 | 3.67 | 43 | 110 | SF3B3_MOUSE | Sf3b3 | 3.54 |
| 41 | 67 | SRRM2_MOUSE | Srrm2 | 3.34 | 28 | 49 | SRRM2_MOUSE | Srrm2 | 3.3 |
| 40 | 45 | CKAP5_MOUSE | Ckap5 | 3.2 | 47 | 53 | CKAP5_MOUSE | Ckap5 | 3.29 |
| 38 | 235 | TIF1B_MOUSE | Trim28 | 3.8 | 43 | 192 | TIF1B_MOUSE | Trim28 | 3.75 |
| 37 | 43 | UTP20_MOUSE | Utp20 | 3.54 | 38 | 44 | UTP20_MOUSE | Utp20 | 3.2 |
| 36 | 51 | SP16H_MOUSE | Supt16h | 3.48 | 46 | 67 | SP16H_MOUSE | Supt16h | 3.35 |
| 36 | 38 | A2ANY6_MOUSE | Mdn1 | 3.33 | 10 | 10 | A2ANY6_MOUSE | Mdn1 | 3.25 |
| 34 | 103 | LITD1_MOUSE | L1td1 | 3.17 | 32 | 79 | LITD1_MOUSE | L1td1 | 3.2 |
| 34 | 41 | INT1_MOUSE | Ints1 | 3.49 | 26 | 30 | INT1_MOUSE | Ints1 | 3.33 |
| 33 | 73 | CDC5L_MOUSE | Cdc5l | 3.61 | 30 | 54 | CDC5L_MOUSE | Cdc5l | 3.97 |
| 33 | 57 | RPB2_MOUSE | Polr2b | 3.56 | 36 | 55 | RPB2_MOUSE | Polr2b | 3.63 |
| 33 | 36 | MYH10_MOUSE | Myh10 | 3.61 | 20 | 24 | MYH10_MOUSE | Myh10 | 3.69 |
| 32 | 37 | A2A654_MOUSE | Bptf | 3.19 | 30 | 37 | A2A654_MOUSE | Bptf | 3.07 |
| 31 | 49 | Q3UJB0_MOUSE | Sf3b2 | 3.64 | 38 | 60 | Q3UJB0_MOUSE | Sf3b2 | 3.67 |
| 31 | 39 | DIDO1_MOUSE | Dido1 | 3.6 | 31 | 43 | DIDO1_MOUSE | Dido1 | 3.43 |
| 31 | 37 | ACINU_MOUSE | Acin1 | 3.14 | 24 | 30 | ACINU_MOUSE | Acin1 | 3.1 |
| 31 | 35 | G3X9B1_MOUSE | Heatr1 | 3.11 | 29 | 35 | G3X9B1_MOUSE | Heatr1 | 3.08 |
| 30 | 71 | HNRPM_MOUSE | Hnrnpm | 3.29 | 38 | 85 | HNRPM_MOUSE | Hnrnpm | 3.18 |
| 30 | 44 | PININ_MOUSE | Pnn | 3.27 | 25 | 41 | PININ_MOUSE | Pnn | 3.6 |
| 30 | 35 | RRP5_MOUSE | Pdcd11 | 3.3 | 23 | 29 | RRP5_MOUSE | Pdcd11 | 3.27 |
| 29 | 58 | DDX21_MOUSE | Ddx21 | 3.28 | 32 | 53 | DDX21_MOUSE | Ddx21 | 3.17 |
| 29 | 38 | SPT5H_MOUSE | Supt5h | 3.56 | 33 | 47 | SPT5H_MOUSE | Supt5h | 3.69 |
| 29 | 37 | E9PVX6_MOUSE | Mki67 | 3.35 | 32 | 37 | E9PVX6_MOUSE | Mki67 | 3.48 |
| 29 | 32 | NPA1P_MOUSE | Urb1 | 3.11 | 16 | 18 | NPA1P_MOUSE | Urb1 | 3.17 |
| 28 | 81 | TEX10_MOUSE | Tex10 | 3.43 | 31 | 66 | TEX10_MOUSE | Tex10 | 3.45 |
| 28 | 43 | D3Z0M9_MOUSE | Ddx23 | 3.09 | 12 | 12 | D3Z0M9_MOUSE | Ddx23 | 3.05 |
| 28 | 40 | SYMPK_MOUSE | Sympk | 3.69 | 25 | 35 | SYMPK_MOUSE | Sympk | 3.35 |
| 28 | 39 | ZN638_MOUSE | Znf638 | 3.29 | 42 | 62 | ZN638_MOUSE | Znf638 | 3.4 |
| 28 | 38 | SOGA2_MOUSE | Soga2 | 3.29 | 17 | 18 | SOGA2_MOUSE | Soga2 | 3.25 |
| 28 | 34 | CPSF1_MOUSE | Cpsf1 | 3.38 | 22 | 26 | CPSF1_MOUSE | Cpsf1 | 3.52 |
| 27 | 29 | MYH9_MOUSE | Myh9 | 3.99 | 18 | 20 | MYH9_MOUSE | Myh9 | 3.94 |
| 27 | 27 | MGAP_MOUSE | Mga | 3.12 | 35 | 43 | MGAP_MOUSE | Mga | 3.26 |
| 26 | 28 | TRIPC_MOUSE | Trip12 | 3.65 | 38 | 50 | TRIPC_MOUSE | Trip12 | 3.46 |
| 25 | 45 | SMRCD_MOUSE | Smarcad1 | 3.8 | 27 | 49 | SMRCD_MOUSE | Smarcad1 | 3.73 |
| 25 | 38 | AQR_MOUSE | Aqr | 3.67 | 26 | 37 | AQR_MOUSE | Aqr | 3.52 |
| 25 | 31 | F208A_MOUSE | Fam208a | 3.28 | 31 | 39 | F208A_MOUSE | Fam208a | 3.3 |
| 24 | 49 | NOP56_MOUSE | Nop56 | 3.54 | 24 | 48 | NOP56_MOUSE | Nop56 | 3.7 |
| 24 | 48 | TIF1A_MOUSE | Trim24 | 4.04 | 22 | 37 | TIF1A_MOUSE | Trim24 | 3.8 |
| 24 | 32 | DDX46_MOUSE | Ddx46 | 3.49 | 23 | 30 | DDX46_MOUSE | Ddx46 | 3.37 |
| 24 | 29 | BCLF1_MOUSE | Bclaf1 | 3.42 | 26 | 36 | BCLF1_MOUSE | Bclaf1 | 3.3 |
| 23 | 69 | HSP7C_MOUSE | Hspa8 | 3.47 | 27 | 70 | HSP7C_MOUSE | Hspa8 | 3.5 |
| 23 | 52 | DDX5_MOUSE | Ddx5 | 3.01 | 20 | 37 | DDX5_MOUSE | Ddx5 | 3.02 |
| 23 | 38 | SYF1_MOUSE | Xab2 | 3.55 | 26 | 40 | SYF1_MOUSE | Xab2 | 3.42 |
| 23 | 28 | SON_MOUSE | Son | 3.32 | 15 | 17 | SON_MOUSE | Son | 3.22 |
| 23 | 28 | B2RQG2_MOUSE | Phf3 | 3.4 | 15 | 16 | B2RQG2_MOUSE | Phf3 | 3.32 |
| 22 | 64 | LAS1L_MOUSE | Las1l | 3.43 | 27 | 59 | LAS1L_MOUSE | Las1l | 3.46 |
| 22 | 37 | HELLS_MOUSE | Hells | 3.43 | 22 | 31 | HELLS_MOUSE | Hells | 3.62 |
| 22 | 31 | NOG1_MOUSE | Gtpbp4 | 3.12 | 15 | 21 | NOG1_MOUSE | Gtpbp4 | 3.12 |
| 22 | 31 | RED_MOUSE | Ik | 3.19 | 24 | 32 | RED_MOUSE | Ik | 3.41 |
| 22 | 30 | MATR3_MOUSE | Matr3 | 3.22 | 20 | 27 | MATR3_MOUSE | Matr3 | 3.47 |
| 22 | 29 | CRNL1_MOUSE | Crnkl1 | 2.99 | 21 | 29 | CRNL1_MOUSE | Crnkl1 | 2.93 |
| 22 | 29 | CHD8_MOUSE | Chd8 | 3.72 | 25 | 33 | CHD8_MOUSE | Chd8 | 3.23 |
| 22 | 25 | RPRD2_MOUSE | Rprd2 | 3.38 | 20 | 23 | RPRD2_MOUSE | Rprd2 | 3.57 |
| 21 | 59 | DDX17_MOUSE | Ddx17 | 2.95 | 23 | 43 | DDX17_MOUSE | Ddx17 | 3.14 |
| 21 | 55 | DHX15_MOUSE | Dhx15 | 3.2 | 30 | 61 | DHX15_MOUSE | Dhx15 | 3.32 |
| 21 | 38 | PR40A_MOUSE | Prpf40a | 3.27 | 19 | 41 | PR40A_MOUSE | Prpf40a | 3.39 |
| 21 | 36 | THOC1_MOUSE | Thoc1 | 3.41 | 20 | 29 | THOC1_MOUSE | Thoc1 | 3.63 |
| 21 | 34 | TR150_MOUSE | Thrap3 | 3.3 | 25 | 40 | TR150_MOUSE | Thrap3 | 3 |
| 21 | 33 | SNW1_MOUSE | Snw1 | 3.37 | 22 | 34 | SNW1_MOUSE | Snw1 | 3.06 |
| 21 | 32 | SALL4_MOUSE | Sall4 | 3.75 | 15 | 21 | SALL4_MOUSE | Sall4 | 4.04 |
| 21 | 28 | TFP11_MOUSE | Tfip11 | 3.22 | 21 | 23 | TFP11_MOUSE | Tfip11 | 3.19 |
| 21 | 24 | PRP4B_MOUSE | Prpf4b | 3.68 | 18 | 20 | PRP4B_MOUSE | Prpf4b | 3.49 |
| 20 | 24 | CHD1_MOUSE | Chd1 | 3.58 | 19 | 21 | CHD1_MOUSE | Chd1 | 3.12 |
| 20 | 23 | EP400_MOUSE | Ep400 | 3.28 | 18 | 20 | EP400_MOUSE | Ep400 | 3.45 |
| 20 | 22 | E9Q7L1_MOUSE | Urb2 | 3.02 | 13 | 18 | E9Q7L1_MOUSE | Urb2 | 3.22 |
| 19 | 37 | LIN41_MOUSE | Trim71 | 3.41 | 15 | 22 | LIN41_MOUSE | Trim71 | 3.63 |
| 19 | 37 | NUCL_MOUSE | Ncl | 3.18 | 22 | 33 | NUCL_MOUSE | Ncl | 3.35 |
| 19 | 29 | NSF_MOUSE | Nsf | 3.43 | 21 | 25 | NSF_MOUSE | Nsf | 3.4 |
| 19 | 29 | YLPM1_MOUSE | Ylpm1 | 3.49 | 17 | 21 | YLPM1_MOUSE | Ylpm1 | 3.24 |
| 19 | 26 | SMCA5_MOUSE | Smarca5 | 3.09 | 28 | 37 | SMCA5_MOUSE | Smarca5 | 3.25 |
| 19 | 21 | MSH6_MOUSE | Msh6 | 3.43 | 26 | 30 | MSH6_MOUSE | Msh6 | 3.3 |
| 19 | 21 | TRRAP_MOUSE | Trrap | 2.88 | 20 | 22 | TRRAP_MOUSE | Trrap | 3.12 |
| 18 | 53 | DDX3L_MOUSE | D1Pas1 | 3.07 | 18 | 33 | DDX3L_MOUSE | D1Pas1 | 3.32 |
| 18 | 42 | PUF60_MOUSE | Puf60 | 3.62 | 16 | 27 | PUF60_MOUSE | Puf60 | 3.75 |
| 18 | 24 | LMNB1_MOUSE | Lmnb1 | 3.26 | 20 | 24 | LMNB1_MOUSE | Lmnb1 | 3.08 |
| 18 | 22 | THOC2_MOUSE | Thoc2 | 3.32 | 18 | 26 | THOC2_MOUSE | Thoc2 | 3.45 |
| 18 | 22 | F208B_MOUSE | Fam208b | 3.44 | 12 | 13 | F208B_MOUSE | Fam208b | 3.28 |
| 18 | 20 | PRP6_MOUSE | Prpf6 | 2.92 | 10 | 11 | PRP6_MOUSE | Prpf6 | 3.2 |
| 18 | 20 | MSH2_MOUSE | Msh2 | 3.56 | 11 | 11 | MSH2_MOUSE | Msh2 | 3.81 |
| 18 | 20 | BAZ2A_MOUSE | Baz2a | 3.13 | 18 | 21 | BAZ2A_MOUSE | Baz2a | 3.26 |
| 18 | 20 | NFRKB_MOUSE | Nfrkb | 3.3 | 11 | 13 | NFRKB_MOUSE | Nfrkb | 3.01 |
| 18 | 19 | DHX8_MOUSE | Dhx8 | 3.36 | 14 | 20 | DHX8_MOUSE | Dhx8 | 3.42 |
| 18 | 19 | MINT_MOUSE | Spen | 3.25 | 17 | 17 | MINT_MOUSE | Spen | 2.58 |
| 18 | 19 | MAP1B_MOUSE | Map1b | 3.9 | 3 | 3 | MAP1B_MOUSE | Map1b | 2.93 |
| 17 | 26 | IF2B1_MOUSE | Igf2bp1 | 3.46 | 8 | 9 | IF2B1_MOUSE | Igf2bp1 | 3.3 |
| 17 | 25 | MTA2_MOUSE | Mta2 | 3.35 | 12 | 17 | MTA2_MOUSE | Mta2 | 3.39 |
| 17 | 24 | PAF1_MOUSE | Paf1 | 3.57 | 24 | 35 | PAF1_MOUSE | Paf1 | 3.7 |
| 17 | 23 | Q3TA68_MOUSE | Wdr36 | 3.34 | 16 | 26 | Q3TA68_MOUSE | Wdr36 | 3.46 |
| 17 | 22 | CTR9_MOUSE | Ctr9 | 3.28 | 20 | 27 | CTR9_MOUSE | Ctr9 | 3.1 |
| 17 | 17 | NSD1_MOUSE | Nsd1 | 3.38 | 26 | 29 | NSD1_MOUSE | Nsd1 | 3.32 |
| 17 | 17 | CNOT1_MOUSE | Cnot1 | 3.1 | 20 | 23 | CNOT1_MOUSE | Cnot1 | 3.23 |
| 16 | 50 | SENP3_MOUSE | Senp3 | 3.16 | 16 | 38 | SENP3_MOUSE | Senp3 | 3.36 |
| 16 | 41 | NOP58_MOUSE | Nop58 | 3.83 | 15 | 39 | NOP58_MOUSE | Nop58 | 4.25 |
| 16 | 32 | PRPF3_MOUSE | Prpf3 | 3.21 | 19 | 28 | PRPF3_MOUSE | Prpf3 | 3.7 |
| 16 | 28 | RU17_MOUSE | Snrnp70 | 2.65 | 15 | 22 | RU17_MOUSE | Snrnp70 | 2.71 |
| 16 | 23 | NUP93_MOUSE | Nup93 | 2.96 | 10 | 10 | NUP93_MOUSE | Nup93 | 3.35 |
| 16 | 23 | HCFC1_MOUSE | Hcfc1 | 3.08 | 13 | 17 | HCFC1_MOUSE | Hcfc1 | 2.95 |
| 16 | 20 | APC1_MOUSE | Anapc1 | 3.35 | 11 | 11 | APC1_MOUSE | Anapc1 | 3.12 |
| 16 | 17 | ELYS_MOUSE | Ahctf1 | 3.15 | 24 | 27 | ELYS_MOUSE | Ahctf1 | 3.2 |
| 16 | 17 | KDM5B_MOUSE | Kdm5b | 3.06 | 18 | 20 | KDM5B_MOUSE | Kdm5b | 3.13 |
| 16 | 17 | TF3C1_MOUSE | Gtf3c1 | 3.25 | 11 | 11 | TF3C1_MOUSE | Gtf3c1 | 2.85 |
| 16 | 17 | PB1_MOUSE | Pbrm1 | 3.38 | 8 | 8 | PB1_MOUSE | Pbrm1 | 3.52 |
| 16 | 17 | CUL7_MOUSE | Cul7 | 3.4 | 4 | 4 | CUL7_MOUSE | Cul7 | 2.95 |
| 15 | 28 | SSRP1_MOUSE | Ssrp1 | 3.04 | 18 | 28 | SSRP1_MOUSE | Ssrp1 | 3.24 |
| 15 | 26 | P66A_MOUSE | Gatad2a | 3.47 | 16 | 28 | P66A_MOUSE | Gatad2a | 3.56 |
| 15 | 21 | SAFB2_MOUSE | Safb2 | 3.19 | 9 | 11 | SAFB2_MOUSE | Safb2 | 3.34 |
| 15 | 20 | SR140_MOUSE | U2surp | 3.01 | 19 | 27 | SR140_MOUSE | U2surp | 3.16 |
| 15 | 19 | WBP11_MOUSE | Wbp11 | 3.46 | 20 | 29 | WBP11_MOUSE | Wbp11 | 3.57 |
| 15 | 16 | B1AWL4_MOUSE | Zfp462 | 3.3 | 12 | 14 | B1AWL4_MOUSE | Zfp462 | 3.4 |
| 15 | 16 | SIN3A_MOUSE | Sin3a | 2.94 | 9 | 11 | SIN3A_MOUSE | Sin3a | 3.2 |
| 15 | 16 | B9EJ54_MOUSE | Nup205 | 2.93 | 8 | 8 | B9EJ54_MOUSE | Nup205 | 2.93 |
| 15 | 16 | RBP2_MOUSE | Ranbp2 | 3.02 | 6 | 6 | RBP2_MOUSE | Ranbp2 | 3.18 |
| 14 | 33 | RBM39_MOUSE | Rbm39 | 3.5 | 17 | 36 | RBM39_MOUSE | Rbm39 | 3.64 |
| 14 | 31 | PELP1_MOUSE | Pelp1 | 3.86 | 13 | 25 | PELP1_MOUSE | Pelp1 | 3.72 |
| 14 | 27 | GRP78_MOUSE | Hspa5 | 3.59 | 18 | 30 | GRP78_MOUSE | Hspa5 | 3.63 |
| 14 | 25 | KIF22_MOUSE | Kif22 | 3.49 | 20 | 29 | KIF22_MOUSE | Kif22 | 3.47 |
| 14 | 20 | EHMT2_MOUSE | Ehmt2 | 3.62 | 11 | 13 | EHMT2_MOUSE | Ehmt2 | 3.53 |
| 14 | 18 | TPX2_MOUSE | Tpx2 | 3.21 | 24 | 31 | TPX2_MOUSE | Tpx2 | 3.37 |
| 14 | 18 | SMCA4_MOUSE | Smarca4 | 3.95 | 13 | 19 | SMCA4_MOUSE | Smarca4 | 3.62 |
| 14 | 17 | OGT1_MOUSE | Ogt | 3.47 | 0 | 0 | na | na | 0 |
| 14 | 17 | TAF6_MOUSE | Taf6 | 3.09 | 13 | 15 | TAF6_MOUSE | Taf6 | 3.33 |
| 14 | 17 | ORC1_MOUSE | Orc1 | 3.12 | 19 | 22 | ORC1_MOUSE | Orc1 | 3.08 |
| 14 | 16 | CLAP1_MOUSE | Clasp1 | 3.5 | 15 | 19 | CLAP1_MOUSE | Clasp1 | 3.28 |
| 14 | 16 | EHMT1_MOUSE | Ehmt1 | 2.98 | 13 | 14 | EHMT1_MOUSE | Ehmt1 | 3.34 |
| 14 | 16 | CHD7_MOUSE | Chd7 | 3 | 11 | 11 | CHD7_MOUSE | Chd7 | 2.9 |
| 14 | 15 | RFC1_MOUSE | Rfc1 | 3.18 | 15 | 20 | RFC1_MOUSE | Rfc1 | 3.16 |
| 14 | 15 | VIR_MOUSE | Kiaa1429 | 3.25 | 10 | 11 | VIR_MOUSE | Kiaa1429 | 3.65 |
| 14 | 14 | SPTN1_MOUSE | Sptan1 | 3.35 | 6 | 6 | SPTN1_MOUSE | Sptan1 | 3.2 |
| 13 | 43 | NOL9_MOUSE | Nol9 | 3.6 | 16 | 46 | NOL9_MOUSE | Nol9 | 3.35 |
| 13 | 21 | RBM14_MOUSE | Rbm14 | 2.66 | 19 | 29 | RBM14_MOUSE | Rbm14 | 2.67 |
| 13 | 17 | ODFP2_MOUSE | Odf2 | 3.38 | 13 | 13 | ODFP2_MOUSE | Odf2 | 3.55 |
| 13 | 17 | RRP12_MOUSE | Rrp12 | 3.01 | 18 | 21 | RRP12_MOUSE | Rrp12 | 3.1 |
| 13 | 17 | SMRC1_MOUSE | Smarcc1 | 3.24 | 10 | 12 | SMRC1_MOUSE | Smarcc1 | 3.01 |
| 13 | 15 | KIF11_MOUSE | Kif11 | 3.25 | 19 | 20 | KIF11_MOUSE | Kif11 | 2.94 |
| 13 | 15 | ATRX_MOUSE | Atrx | 3.08 | 14 | 15 | ATRX_MOUSE | Atrx | 3.09 |
| 13 | 15 | CND1_MOUSE | Ncapd2 | 3.03 | 4 | 5 | CND1_MOUSE | Ncapd2 | 3.3 |
| 13 | 14 | SMC1A_MOUSE | Smc1a | 3.19 | 13 | 14 | SMC1A_MOUSE | Smc1a | 2.95 |
| 12 | 22 | ALBU_MOUSE | Alb | 3.45 | 10 | 17 | ALBU_MOUSE | Alb | 3.65 |
| 12 | 21 | HNRPL_MOUSE | Hnrnpl | 3.6 | 9 | 13 | HNRPL_MOUSE | Hnrnpl | 3.47 |
| 12 | 18 | KAT7_MOUSE | Kat7 | 3.62 | 13 | 16 | KAT7_MOUSE | Kat7 | 3.52 |
| 12 | 17 | ARHG2_MOUSE | Arhgef2 | 3.27 | 11 | 11 | ARHG2_MOUSE | Arhgef2 | 3.31 |
| 12 | 17 | BEND3_MOUSE | Bend3 | 3.4 | 11 | 17 | BEND3_MOUSE | Bend3 | 3.17 |
| 12 | 17 | A2AJ72_MOUSE | Fubp3 | 3.18 | 12 | 17 | A2AJ72_MOUSE | Fubp3 | 3.3 |
| 12 | 16 | NCOA5_MOUSE | Ncoa5 | 3.93 | 12 | 18 | NCOA5_MOUSE | Ncoa5 | 3.25 |

| WT | | | | | Dnmt1-null | | | | |
|---|---|---|---|---|---|---|---|---|---|
| Uniq | Tota | reference | Gene Syn | AVG | Uniq | Tota | reference | Gene Syn | AVG |
| 12 | 15 | CX023_MOUSE | CXorf23 H | | 7 | 7 | CX023_MOUSE | CXorf23 H | |
| 12 | 15 | INT7_MOUSE | Ints7 | 3.57 | 13 | 14 | INT7_MOUSE | Ints7 | 3.85 |
| 12 | 14 | Q0VGV0_MOUSE | Zfp568 | 3.41 | 11 | 13 | Q0VGV0_MOUSE | Zfp568 | 3.48 |
| 12 | 14 | LRC40_MOUSE | Lrrc40 | 3.06 | 11 | 13 | LRC40_MOUSE | Lrrc40 | 3.46 |
| 12 | 14 | U5S1_MOUSE | Eftud2 | 3.24 | 17 | 24 | U5S1_MOUSE | Eftud2 | 3.08 |
| 12 | 14 | AHDC1_MOUSE | Ahdc1 | 3.95 | 12 | 15 | AHDC1_MOUSE | Ahdc1 | 3.91 |
| 12 | 14 | ZMYM2_MOUSE | Zmym2 | 3.33 | 11 | 12 | ZMYM2_MOUSE | Zmym2 | 3.51 |
| 12 | 13 | CDC27_MOUSE | Cdc27 | 3.19 | 8 | 8 | CDC27_MOUSE | Cdc27 | 3.06 |
| 12 | 13 | A2BIE1_MOUSE | Qser1 | 3.51 | 18 | 18 | A2BIE1_MOUSE | Qser1 | 3.48 |
| 12 | 13 | FNBP4_MOUSE | Fnbp4 | 3.6 | 11 | 13 | FNBP4_MOUSE | Fnbp4 | 3.78 |
| 12 | 13 | NU133_MOUSE | Nup133 | 3.65 | 11 | 12 | NU133_MOUSE | Nup133 | 3.6 |
| 12 | 13 | FINC_MOUSE | Fn1 | 3.47 | 3 | 3 | FINC_MOUSE | Fn1 | 4.04 |
| 11 | 25 | NPM_MOUSE | Npm1 | 4.22 | 9 | 18 | NPM_MOUSE | Npm1 | 4.65 |
| 11 | 19 | HNRPK_MOUSE | Hnrnpk | 3.69 | 9 | 14 | HNRPK_MOUSE | Hnrnpk | 3.85 |
| 11 | 16 | SRRM1_MOUSE | Srrm1 | 4.03 | 8 | 13 | SRRM1_MOUSE | Srrm1 | 4.02 |
| 11 | 15 | Q8BVY0_MOUSE | Rsl1d1 | 3.83 | 10 | 13 | Q8BVY0_MOUSE | Rsl1d1 | 3.58 |
| 11 | 15 | ARI3B_MOUSE | Arid3b | 3.08 | 11 | 13 | ARI3B_MOUSE | Arid3b | 3.01 |
| 11 | 15 | ORC3_MOUSE | Orc3 | 3.28 | 14 | 18 | ORC3_MOUSE | Orc3 | 3.37 |
| 11 | 14 | NCBP1_MOUSE | Ncbp1 | 3.42 | 11 | 15 | NCBP1_MOUSE | Ncbp1 | 3.28 |
| 11 | 14 | ZO2_MOUSE | Tjp2 | 3.39 | 13 | 15 | ZO2_MOUSE | Tjp2 | 3.4 |
| 11 | 12 | SDA1_MOUSE | Sdad1 | 2.8 | 13 | 13 | SDA1_MOUSE | Sdad1 | 2.81 |
| 11 | 12 | RAGP1_MOUSE | Rangap1 | 3.81 | 14 | 15 | RAGP1_MOUSE | Rangap1 | 3.48 |
| 11 | 12 | CDK12_MOUSE | Cdk12 | 3.37 | 9 | 10 | CDK12_MOUSE | Cdk12 | 3.45 |
| 11 | 11 | CDC23_MOUSE | Cdc23 | 2.78 | 3 | 3 | CDC23_MOUSE | Cdc23 | 2.46 |
| 11 | 11 | EXOC3_MOUSE | Exoc3 | 2.94 | 9 | 9 | EXOC3_MOUSE | Exoc3 | 2.79 |
| 11 | 11 | RBAK_MOUSE | Rbak | 2.7 | 11 | 12 | RBAK_MOUSE | Rbak | 2.79 |
| 11 | 11 | INO80_MOUSE | Ino80 | 3.32 | 16 | 20 | INO80_MOUSE | Ino80 | 3.23 |
| 11 | 11 | FANCI_MOUSE | Fanci | 3.3 | 6 | 7 | FANCI_MOUSE | Fanci | 3.37 |
| 11 | 11 | EMSY_MOUSE | Emsy | 2.96 | 6 | 6 | EMSY_MOUSE | Emsy | 3.22 |
| 10 | 32 | A2A9J5_MOUSE | Gm13242 | 3.5 | 11 | 33 | A2A9J5_MOUSE | Gm13242 | 3.66 |
| 10 | 20 | HDAC1_MOUSE | Hdac1 | 3.87 | 10 | 15 | HDAC1_MOUSE | Hdac1 | 3.42 |
| 10 | 16 | G3X9T1_MOUSE | Zfp715 | 3 | 6 | 7 | G3X9T1_MOUSE | Zfp715 | 2.98 |
| 10 | 15 | HNRPQ_MOUSE | Syncrip | 2.8 | 7 | 7 | HNRPQ_MOUSE | Syncrip | 2.98 |
| 10 | 15 | TOP1_MOUSE | Top1 | 2.78 | 9 | 12 | TOP1_MOUSE | Top1 | 2.99 |
| 10 | 15 | KHDR1_MOUSE | Khdrbs1 | 2.83 | 9 | 14 | KHDR1_MOUSE | Khdrbs1 | 3.48 |
| 10 | 15 | MTA1_MOUSE | Mta1 | 3.25 | 12 | 14 | MTA1_MOUSE | Mta1 | 3.13 |
| 10 | 15 | THOC5_MOUSE | Thoc5 | 3.2 | 15 | 22 | THOC5_MOUSE | Thoc5 | 3.35 |
| 10 | 15 | IWS1_MOUSE | Iws1 | 3.63 | 8 | 16 | IWS1_MOUSE | Iws1 | 3.51 |
| 10 | 14 | DDX52_MOUSE | Ddx52 | 3.35 | 12 | 15 | DDX52_MOUSE | Ddx52 | 4.29 |
| 10 | 13 | FIP1_MOUSE | Fip1l1 | 3.58 | 7 | 13 | FIP1_MOUSE | Fip1l1 | 3.66 |
| 10 | 13 | P66B_MOUSE | Gatad2b | 3.64 | 8 | 9 | P66B_MOUSE | Gatad2b | 2.91 |
| 10 | 13 | CTBL1_MOUSE | Ctnnbl1 | 3.5 | 13 | 19 | CTBL1_MOUSE | Ctnnbl1 | 3.32 |
| 10 | 13 | INT10_MOUSE | Ints10 | 3.04 | 14 | 17 | INT10_MOUSE | Ints10 | 3.34 |
| 10 | 13 | MCM7_MOUSE | Mcm7 | 3.46 | 15 | 22 | MCM7_MOUSE | Mcm7 | 3.44 |
| 10 | 13 | SCAF8_MOUSE | Scaf8 | 3.93 | 11 | 16 | SCAF8_MOUSE | Scaf8 | 3.87 |
| 10 | 13 | SMC3_MOUSE | Smc3 | 2.88 | 12 | 13 | SMC3_MOUSE | Smc3 | 3.25 |
| 10 | 12 | ILF3_MOUSE | Ilf3 | 2.98 | 6 | 6 | ILF3_MOUSE | Ilf3 | 3.45 |
| 10 | 12 | MUM1_MOUSE | Mum1 | 3.34 | 10 | 12 | MUM1_MOUSE | Mum1 | 3.5 |
| 10 | 12 | CLAP2_MOUSE | Clasp2 | 3.12 | 12 | 15 | CLAP2_MOUSE | Clasp2 | 3.24 |
| 10 | 12 | E9PWW9_MOUSE | Rsf1 | 3.75 | 9 | 12 | E9PWW9_MOUSE | Rsf1 | 3.21 |
| 10 | 12 | ZCH18_MOUSE | Zc3h18 | 3.11 | 9 | 10 | ZCH18_MOUSE | Zc3h18 | 3.67 |
| 10 | 11 | RBM28_MOUSE | Rbm28 | 3.1 | 8 | 11 | RBM28_MOUSE | Rbm28 | 2.68 |
| 10 | 11 | ASUN_MOUSE | Asun | 2.73 | 11 | 11 | ASUN_MOUSE | Asun | 2.85 |
| 10 | 11 | GTSE1_MOUSE | Gtse1 | 3.53 | 13 | 17 | GTSE1_MOUSE | Gtse1 | 3.41 |
| 10 | 11 | ZC3HE_MOUSE | Zc3h14 | 3.62 | 18 | 22 | ZC3HE_MOUSE | Zc3h14 | 3.63 |
| 10 | 11 | ZMYM4_MOUSE | Zmym4 | 3.02 | 16 | 18 | ZMYM4_MOUSE | Zmym4 | 3.66 |
| 10 | 10 | ZN292_MOUSE | Zfp292 | 3.36 | 11 | 12 | ZN292_MOUSE | Zfp292 | 3.27 |
| 10 | 10 | NU160_MOUSE | Nup160 | 2.93 | 10 | 12 | NU160_MOUSE | Nup160 | 3.36 |
| 10 | 10 | ANLN_MOUSE | Anln | 3.37 | 8 | 8 | ANLN_MOUSE | Anln | 3.56 |
| 10 | 10 | TTF2_MOUSE | Ttf2 | 3.05 | 6 | 7 | TTF2_MOUSE | Ttf2 | 2.79 |
| 9 | 13 | DDX18_MOUSE | Ddx18 | 2.82 | 6 | 7 | DDX18_MOUSE | Ddx18 | 3.04 |
| 9 | 13 | CSTF3_MOUSE | Cstf3 | 3.73 | 12 | 14 | CSTF3_MOUSE | Cstf3 | 3.58 |
| 9 | 12 | Q8BIV1_MOUSE | Zfp719 | 3.1 | 6 | 6 | Q8BIV1_MOUSE | Zfp719 | 3.49 |
| 9 | 12 | CQ085_MOUSE | Ncbp3 | | 8 | 10 | CQ085_MOUSE | Ncbp3 | |
| 9 | 12 | SPA3K_MOUSE | Serpina3k | 3.12 | 8 | 9 | SPA3K_MOUSE | Serpina3k | 3.51 |
| 9 | 11 | JADE1_MOUSE | Phf17 | 3.26 | 8 | 9 | JADE1_MOUSE | Phf17 | 3.27 |
| 9 | 11 | MFAP1_MOUSE | Mfap1 | 2.99 | 11 | 13 | MFAP1_MOUSE | Mfap1 | 3.25 |
| 9 | 11 | F7BJB9_MOUSE | Morc3 | 3.41 | 7 | 7 | F7BJB9_MOUSE | Morc3 | 3.39 |
| 9 | 10 | KANL3_MOUSE | Kansl3 | 3.44 | 1 | 1 | KANL3_MOUSE | Kansl3 | 2.01 |
| 9 | 10 | DDX55_MOUSE | Ddx55 | 3.68 | 3 | 3 | DDX55_MOUSE | Ddx55 | 4.14 |
| 9 | 10 | UTP18_MOUSE | Utp18 | 2.71 | 6 | 8 | UTP18_MOUSE | Utp18 | 3.64 |
| 9 | 10 | K0020_MOUSE | Kiaa0020 | 3.47 | 10 | 10 | K0020_MOUSE | Kiaa0020 | 3.26 |
| 9 | 10 | UBF1_MOUSE | Ubtf | 3.29 | 15 | 18 | UBF1_MOUSE | Ubtf | 3.04 |
| 9 | 10 | SALL1_MOUSE | Sall1 | 3.89 | 11 | 11 | SALL1_MOUSE | Sall1 | 3.73 |
| 9 | 10 | RBBP6_MOUSE | Rbbp6 | 3.33 | 7 | 7 | RBBP6_MOUSE | Rbbp6 | 3 |
| 9 | 9 | HS90A_MOUSE | Hsp90aa1 | 2.77 | 4 | 4 | HS90A_MOUSE | Hsp90aa1 | 2.33 |
| 9 | 9 | CPSF2_MOUSE | Cpsf2 | 3.94 | 5 | 6 | CPSF2_MOUSE | Cpsf2 | 3.44 |
| 9 | 9 | PP1RA_MOUSE | Ppp1r10 | 3.28 | 6 | 7 | PP1RA_MOUSE | Ppp1r10 | 3.52 |
| 9 | 9 | TF3C4_MOUSE | Gtf3c4 | 3 | 6 | 6 | TF3C4_MOUSE | Gtf3c4 | 2.59 |
| 9 | 9 | Q6PGF5_MOUSE | Bms1 | 3.07 | 15 | 17 | Q6PGF5_MOUSE | Bms1 | 3.33 |
| 9 | 9 | PHRF1_MOUSE | Phrf1 | 2.61 | 9 | 10 | PHRF1_MOUSE | Phrf1 | 2.7 |
| 9 | 9 | POGZ_MOUSE | Pogz | 3.23 | 10 | 10 | POGZ_MOUSE | Pogz | 3.56 |
| 9 | 9 | VPRBP_MOUSE | Vprbp | 2.96 | 8 | 8 | VPRBP_MOUSE | Vprbp | 3.44 |
| 9 | 9 | SMC2_MOUSE | Smc2 | 2.94 | 2 | 2 | SMC2_MOUSE | Smc2 | 3.48 |
| 8 | 20 | SF3A2_MOUSE | Sf3a2 | 3.72 | 9 | 15 | SF3A2_MOUSE | Sf3a2 | 3.72 |
| 8 | 20 | IGG2B_MOUSE | Igh-3 | 3.07 | 6 | 20 | IGG2B_MOUSE | Igh-3 | 3.17 |
| 8 | 14 | ZN569_MOUSE | Znf569 | 3.26 | 7 | 11 | ZN569_MOUSE | Znf569 | 3.19 |
| 8 | 13 | SNUT2_MOUSE | Usp39 | 3.57 | 9 | 14 | SNUT2_MOUSE | Usp39 | 3.41 |
| 8 | 13 | SMCA1_MOUSE | Smarca1 | 3.04 | 7 | 13 | SMCA1_MOUSE | Smarca1 | 2.96 |
| 8 | 13 | TRI33_MOUSE | Trim33 | 3.2 | 5 | 5 | TRI33_MOUSE | Trim33 | 3.75 |
| 8 | 12 | TCPG_MOUSE | Cct3 | 3.09 | 3 | 3 | TCPG_MOUSE | Cct3 | 3.4 |
| 8 | 12 | PPWD1_MOUSE | Ppwd1 | 3.35 | 7 | 9 | PPWD1_MOUSE | Ppwd1 | 3.33 |
| 8 | 12 | NACC1_MOUSE | Nacc1 | 2.96 | 9 | 15 | NACC1_MOUSE | Nacc1 | 3.08 |
| 8 | 11 | CO3_MOUSE | C3 | 3.13 | 4 | 5 | CO3_MOUSE | C3 | 3.35 |
| 8 | 11 | Q9JMG9_MOUSE | Patz1 | 3.5 | 8 | 14 | Q9JMG9_MOUSE | Patz1 | 3.61 |
| 8 | 10 | ZN326_MOUSE | Znf326 | 3.36 | 6 | 9 | ZN326_MOUSE | Znf326 | 3.8 |
| 8 | 10 | SRSF4_MOUSE | Srsf4 | 3.22 | 7 | 9 | SRSF4_MOUSE | Srsf4 | 3.65 |
| 8 | 10 | CPSF3_MOUSE | Cpsf3 | 2.92 | 11 | 11 | CPSF3_MOUSE | Cpsf3 | 3.01 |
| 8 | 10 | E9PYH6_MOUSE | Setd1a | 3.46 | 8 | 9 | E9PYH6_MOUSE | Setd1a | 3.37 |
| 8 | 9 | APC5_MOUSE | Anapc5 | 3.63 | 4 | 5 | APC5_MOUSE | Anapc5 | 4.19 |
| 8 | 9 | NVL_MOUSE | Nvl | 3.42 | 4 | 4 | NVL_MOUSE | Nvl | 3.42 |
| 8 | 9 | SPAT5_MOUSE | Spata5 | 3.91 | 5 | 5 | SPAT5_MOUSE | Spata5 | 3.66 |
| 8 | 9 | CDC16_MOUSE | Cdc16 | 3.2 | 5 | 5 | CDC16_MOUSE | Cdc16 | 3.05 |
| 8 | 9 | LARP7_MOUSE | Larp7 | 2.84 | 6 | 6 | LARP7_MOUSE | Larp7 | 2.99 |
| 8 | 9 | A2ACQ1_MOUSE | Dhx35 | 3.02 | 9 | 9 | A2ACQ1_MOUSE | Dhx35 | 3.33 |
| 8 | 9 | SUZ12_MOUSE | Suz12 | 2.99 | 10 | 13 | SUZ12_MOUSE | Suz12 | 4 |
| 8 | 9 | CHERP_MOUSE | Cherp | 2.52 | 10 | 12 | CHERP_MOUSE | Cherp | 2.62 |
| 8 | 9 | SFSWA_MOUSE | Sfswap | 2.93 | 7 | 7 | SFSWA_MOUSE | Sfswap | 2.78 |
| 8 | 9 | A3KFM7_MOUSE | Chd6 | 3.37 | 7 | 7 | A3KFM7_MOUSE | Chd6 | 3.89 |
| 8 | 9 | RBM27_MOUSE | Rbm27 | 3.49 | 6 | 7 | RBM27_MOUSE | Rbm27 | 2.99 |
| 8 | 8 | DHX33_MOUSE | Dhx33 | 2.74 | 8 | 8 | DHX33_MOUSE | Dhx33 | 3.14 |
| 8 | 8 | APC4_MOUSE | Anapc4 | 3.34 | 9 | 12 | APC4_MOUSE | Anapc4 | 3.12 |
| 8 | 8 | GNL3_MOUSE | Gnl3 | 3.17 | 11 | 12 | GNL3_MOUSE | Gnl3 | 3.42 |
| 8 | 8 | TOP2B_MOUSE | Top2b | 3.33 | 14 | 16 | TOP2B_MOUSE | Top2b | 3.15 |
| 8 | 8 | WDR33_MOUSE | Wdr33 | 3.74 | 12 | 12 | WDR33_MOUSE | Wdr33 | 3.29 |
| 8 | 8 | DDB1_MOUSE | Ddb1 | 2.84 | 8 | 8 | DDB1_MOUSE | Ddb1 | 2.92 |
| 8 | 8 | Q8BIX2_MOUSE | Gcn1l1 | 2.88 | 6 | 6 | Q8BIX2_MOUSE | Gcn1l1 | 3.05 |
| 8 | 8 | SPTB2_MOUSE | Sptbn1 | 3.8 | 5 | 5 | SPTB2_MOUSE | Sptbn1 | 2.82 |
| 7 | 17 | HSP72_MOUSE | Hspa2 | 3.21 | 8 | 12 | HSP72_MOUSE | Hspa2 | 3.6 |
| 7 | 14 | CWC27_MOUSE | Cwc27 | 4.15 | 4 | 5 | CWC27_MOUSE | Cwc27 | 4.51 |
| 7 | 13 | SMCA2_MOUSE | Smarca2 | 3.39 | 5 | 9 | SMCA2_MOUSE | Smarca2 | 3.04 |
| 7 | 12 | Q8VHM5_MOUSE | Hnrnpr | 3.33 | 7 | 9 | Q8VHM5_MOUSE | Hnrnpr | 3.2 |
| 7 | 11 | Q9D654_MOUSE | Zfp84 | 3.96 | 5 | 6 | Q9D654_MOUSE | Zfp84 | 4.6 |
| 7 | 10 | Q8BP18_MOUSE | Zfp101 | 3.62 | 4 | 6 | Q8BP18_MOUSE | Zfp101 | 3.77 |
| 7 | 10 | CSTF2_MOUSE | Cstf2 | 3.53 | 6 | 9 | CSTF2_MOUSE | Cstf2 | 3.06 |
| 7 | 10 | SRP68_MOUSE | Srp68 | 2.78 | 7 | 7 | SRP68_MOUSE | Srp68 | 3.43 |
| 7 | 9 | IMB1_MOUSE | Kpnb1 | 3.1 | 6 | 7 | IMB1_MOUSE | Kpnb1 | 3.13 |
| 7 | 9 | F179B_MOUSE | Fam179b | 3.41 | 6 | 9 | F179B_MOUSE | Fam179b | 3.79 |
| 7 | 9 | SUGP1_MOUSE | Sugp1 | 3.92 | 10 | 11 | SUGP1_MOUSE | Sugp1 | 3.8 |
| 7 | 8 | NU107_MOUSE | Nup107 | 3.12 | 0 | 0 | na | na | 0 |
| 7 | 8 | MCM3_MOUSE | Mcm3 | 3.33 | 1 | 1 | MCM3_MOUSE | Mcm3 | 3.96 |
| 7 | 8 | CKAP2_MOUSE | Ckap2 | 2.86 | 3 | 3 | CKAP2_MOUSE | Ckap2 | 2.87 |
| 7 | 8 | CCD86_MOUSE | Ccdc86 | 2.99 | 3 | 4 | CCD86_MOUSE | Ccdc86 | 3.93 |
| 7 | 8 | Q9DC47_MOUSE | Zfp869 | 3.25 | 4 | 4 | Q9DC47_MOUSE | Zfp869 | 2.96 |
| 7 | 8 | EPC2_MOUSE | Epc2 | 3.12 | 4 | 4 | EPC2_MOUSE | Epc2 | 3.35 |
| 7 | 8 | B1AZ91_MOUSE | Gm13235 | 2.79 | 4 | 4 | B1AZ91_MOUSE | Gm13235 | 3.02 |
| 7 | 8 | EXOSX_MOUSE | Exosc10 | 3.18 | 5 | 5 | EXOSX_MOUSE | Exosc10 | 2.88 |
| 7 | 8 | ARI3A_MOUSE | Arid3a | 3.15 | 7 | 9 | ARI3A_MOUSE | Arid3a | 3.62 |
| 7 | 8 | ZN143_MOUSE | Znf143 | 3.43 | 8 | 12 | ZN143_MOUSE | Znf143 | 3.55 |
| 7 | 8 | DDX51_MOUSE | Ddx51 | 3.56 | 10 | 14 | DDX51_MOUSE | Ddx51 | 3.26 |
| 7 | 8 | Q6NZL1_MOUSE | Dhx37 | 2.74 | 8 | 8 | Q6NZL1_MOUSE | Dhx37 | 2.72 |
| 7 | 8 | A2M_MOUSE | A2m | 3.28 | 4 | 4 | A2M_MOUSE | A2m | 3.52 |
| 7 | 8 | ZC3H4_MOUSE | Zc3h4 | 3.85 | 3 | 3 | ZC3H4_MOUSE | Zc3h4 | 3.27 |
| 7 | 7 | F6T1S3_MOUSE | AW14615 | 2.71 | 4 | 4 | F6T1S3_MOUSE | AW14615 | 2.5 |
| 7 | 7 | MCM6_MOUSE | Mcm6 | 3.23 | 4 | 4 | MCM6_MOUSE | Mcm6 | 3.17 |
| 7 | 7 | CTND1_MOUSE | Ctnnd1 | 3.22 | 4 | 4 | CTND1_MOUSE | Ctnnd1 | 2.76 |
| 7 | 7 | DYN2_MOUSE | Dnm2 | 3.92 | 5 | 5 | DYN2_MOUSE | Dnm2 | 3.97 |
| 7 | 7 | PRP17_MOUSE | Cdc40 | 3.17 | 8 | 9 | PRP17_MOUSE | Cdc40 | 3.52 |
| 7 | 7 | GZF1_MOUSE | Gzf1 | 2.99 | 9 | 9 | GZF1_MOUSE | Gzf1 | 2.74 |
| 7 | 7 | KAT6B_MOUSE | Kat6b | 3.44 | 10 | 11 | KAT6B_MOUSE | Kat6b | 3.36 |
| 7 | 7 | TICRR_MOUSE | Ticrr | 3.13 | 10 | 10 | TICRR_MOUSE | Ticrr | 3.47 |
| 7 | 7 | CHD9_MOUSE | Chd9 | 3.48 | 8 | 8 | CHD9_MOUSE | Chd9 | 3.37 |
| 7 | 7 | TCRG1_MOUSE | Tcerg1 | 2.9 | 6 | 6 | TCRG1_MOUSE | Tcerg1 | 2.76 |
| 7 | 7 | G3X8X0_MOUSE | Dhx16 | 2.82 | 4 | 5 | G3X8X0_MOUSE | Dhx16 | 3.46 |
| 7 | 7 | ARI1A_MOUSE | Arid1a | 2.77 | 4 | 4 | ARI1A_MOUSE | Arid1a | 2.72 |
| 6 | 13 | TRI26_MOUSE | Trim26 | 3.11 | 5 | 6 | TRI26_MOUSE | Trim26 | 2.85 |

| WT | | | | | Dnmt1-null | | | | |
|---|---|---|---|---|---|---|---|---|---|
| Uniq | Tota | reference | Gene Syn | AVG | Uniq | Total | reference | Gene Syn | AVG |
| 6 | 10 | Q8BQ46_MOUSE | Taf15 | 2.98 | 3 | 4 | Q8BQ46_MOUSE | Taf15 | 3.08 |
| 6 | 10 | TFE3_MOUSE | Tfe3 | 3.55 | 9 | 13 | TFE3_MOUSE | Tfe3 | 3.34 |
| 6 | 9 | KIF2C_MOUSE | Kif2c | 2.95 | 3 | 3 | KIF2C_MOUSE | Kif2c | 2.58 |
| 6 | 9 | MYEF2_MOUSE | Myef2 | 2.81 | 6 | 6 | MYEF2_MOUSE | Myef2 | 3.09 |
| 6 | 9 | WDR43_MOUSE | Wdr43 | 2.44 | 8 | 15 | WDR43_MOUSE | Wdr43 | 3.27 |
| 6 | 8 | HNRPU_MOUSE | Hnrnpu | 3.6 | 5 | 5 | HNRPU_MOUSE | Hnrnpu | 3.61 |
| 6 | 8 | Q9CZP3_MOUSE | Zfp655 | 3.35 | 6 | 9 | Q9CZP3_MOUSE | Zfp655 | 3.37 |
| 6 | 8 | SF3A1_MOUSE | Sf3a1 | 3.2 | 6 | 6 | SF3A1_MOUSE | Sf3a1 | 3.31 |
| 6 | 8 | TOX4_MOUSE | Tox4 | 3.68 | 7 | 11 | TOX4_MOUSE | Tox4 | 3.62 |
| 6 | 8 | CALX_MOUSE | Canx | 3.38 | 7 | 10 | CALX_MOUSE | Canx | 3.53 |
| 6 | 8 | Q8CF60_MOUSE | Zfp263 | 3.5 | 7 | 10 | Q8CF60_MOUSE | Zfp263 | 3.74 |
| 6 | 8 | LMBL2_MOUSE | L3mbtl2 | 2.98 | 9 | 10 | LMBL2_MOUSE | L3mbtl2 | 3.2 |
| 6 | 7 | G5E8Z2_MOUSE | Taf4b | 3.88 | 5 | 5 | G5E8Z2_MOUSE | Taf4b | 3.63 |
| 6 | 7 | D3Z5Y8_MOUSE | Gm15446 | 2.67 | 5 | 5 | D3Z5Y8_MOUSE | Gm15446 | 2.63 |
| 6 | 7 | DDX41_MOUSE | Ddx41 | 3.02 | 7 | 7 | DDX41_MOUSE | Ddx41 | 3.67 |
| 6 | 7 | DDX1_MOUSE | Ddx1 | 2.7 | 8 | 8 | DDX1_MOUSE | Ddx1 | 3.36 |
| 6 | 7 | CIR1A_MOUSE | Cirh1a | 2.95 | 9 | 13 | CIR1A_MOUSE | Cirh1a | 3.66 |
| 6 | 7 | NIPBL_MOUSE | Nipbl | 3.21 | 10 | 11 | NIPBL_MOUSE | Nipbl | 3.28 |
| 6 | 7 | CCAR1_MOUSE | Ccar1 | 3.16 | 6 | 7 | CCAR1_MOUSE | Ccar1 | 3.27 |
| 6 | 7 | F6ZDS4_MOUSE | Tpr | 3.1 | 7 | 7 | F6ZDS4_MOUSE | Tpr | 2.76 |
| 6 | 7 | SMC4_MOUSE | Smc4 | 3.13 | 5 | 6 | SMC4_MOUSE | Smc4 | 2.53 |
| 6 | 7 | SOGA1_MOUSE | Soga1 | 2.7 | 6 | 6 | SOGA1_MOUSE | Soga1 | 3.1 |
| 6 | 7 | EDC4_MOUSE | Edc4 | 3.54 | 5 | 5 | EDC4_MOUSE | Edc4 | 3.52 |
| 6 | 7 | BNC2_MOUSE | Bnc2 | 2.99 | 4 | 4 | BNC2_MOUSE | Bnc2 | 3.32 |
| 6 | 7 | BRD8_MOUSE | Brd8 | 3.35 | 3 | 3 | BRD8_MOUSE | Brd8 | 2.74 |
| 6 | 6 | WIZ_MOUSE | Wiz | 2.63 | 1 | 1 | WIZ_MOUSE | Wiz | 2.74 |
| 6 | 6 | TCPZ_MOUSE | Cct6a | 3.76 | 1 | 1 | TCPZ_MOUSE | Cct6a | 3.4 |
| 6 | 6 | Q3TJB1_MOUSE | Zmym1 | 2.79 | 2 | 2 | Q3TJB1_MOUSE | Zmym1 | 3.19 |
| 6 | 6 | BUD13_MOUSE | Bud13 | 3.36 | 3 | 3 | BUD13_MOUSE | Bud13 | 3.36 |
| 6 | 6 | LSG1_MOUSE | Lsg1 | 3.13 | 4 | 4 | LSG1_MOUSE | Lsg1 | 2.81 |
| 6 | 6 | PLK1_MOUSE | Plk1 | 3.02 | 5 | 6 | PLK1_MOUSE | Plk1 | 2.87 |
| 6 | 6 | E9Q5K9_MOUSE | Ythdc1 | 2.64 | 5 | 5 | E9Q5K9_MOUSE | Ythdc1 | 2.19 |
| 6 | 6 | GCP2_MOUSE | Tubgcp2 | 3.2 | 6 | 7 | GCP2_MOUSE | Tubgcp2 | 3.31 |
| 6 | 6 | PABP1_MOUSE | Pabpc1 | 2.99 | 6 | 7 | PABP1_MOUSE | Pabpc1 | 3.36 |
| 6 | 6 | MLH1_MOUSE | Mlh1 | 2.7 | 6 | 6 | MLH1_MOUSE | Mlh1 | 3.19 |
| 6 | 6 | RFX5_MOUSE | Rfx5 | 2.95 | 7 | 9 | RFX5_MOUSE | Rfx5 | 3.18 |
| 6 | 6 | RCOR2_MOUSE | Rcor2 | 3.14 | 8 | 8 | RCOR2_MOUSE | Rcor2 | 3.41 |
| 6 | 6 | MD1L1_MOUSE | Mad1l1 | 3.07 | 12 | 12 | MD1L1_MOUSE | Mad1l1 | 3.05 |
| 6 | 6 | TET1_MOUSE | Tet1 | 3.17 | 9 | 10 | TET1_MOUSE | Tet1 | 3.29 |
| 6 | 6 | SETX_MOUSE | Setx | 3.45 | 7 | 8 | SETX_MOUSE | Setx | 3.31 |
| 6 | 6 | RAD50_MOUSE | Rad50 | 2.6 | 8 | 8 | RAD50_MOUSE | Rad50 | 3.9 |
| 6 | 6 | REST_MOUSE | Rest | 3.7 | 6 | 7 | REST_MOUSE | Rest | 3.34 |
| 6 | 6 | SLTM_MOUSE | Sltm | 3.13 | 6 | 6 | SLTM_MOUSE | Sltm | 3.14 |
| 6 | 6 | INT2_MOUSE | Ints2 | 3.28 | 5 | 5 | INT2_MOUSE | Ints2 | 3.42 |
| 6 | 6 | ATAD5_MOUSE | Atad5 | 2.89 | 4 | 4 | ATAD5_MOUSE | Atad5 | 3.16 |
| 6 | 6 | SAFB1_MOUSE | Safb | 2.88 | 4 | 4 | SAFB1_MOUSE | Safb | 3.93 |
| 6 | 6 | DOCK6_MOUSE | Dock6 | 2.71 | 2 | 2 | DOCK6_MOUSE | Dock6 | 3.22 |
| 6 | 6 | WAPL_MOUSE | Wapal | 2.95 | 1 | 1 | WAPL_MOUSE | Wapal | 2.87 |
| 6 | 6 | CERU_MOUSE | Cp | 2.8 | 1 | 1 | CERU_MOUSE | Cp | 3.17 |
| 6 | 6 | DYHC1_MOUSE | Dync1h1 | 2.86 | 0 | 0 | na | na | 0 |
| 6 | 6 | F8VQ29_MOUSE | Iqgap3 | 3.3 | 0 | 0 | na | na | 0 |
| 6 | 6 | E9Q784_MOUSE | Zc3h13 | 2.93 | 0 | 0 | na | na | 0 |
| 5 | 21 | HS71A_MOUSE | Hspa1a | 3.86 | 5 | 12 | HS71A_MOUSE | Hspa1a | 3.66 |
| 5 | 14 | SF01_MOUSE | Sf1 | 2.93 | 9 | 16 | SF01_MOUSE | Sf1 | 3.15 |
| 5 | 8 | NOG2_MOUSE | Gnl2 | 3.49 | 2 | 2 | NOG2_MOUSE | Gnl2 | 4.21 |
| 5 | 8 | EWS_MOUSE | Ewsr1 | 4.04 | 5 | 8 | EWS_MOUSE | Ewsr1 | 4.22 |
| 5 | 7 | INT8_MOUSE | Ints8 | 3.29 | 0 | 0 | na | na | 0 |
| 5 | 7 | ZSC10_MOUSE | Zscan10 | 3.68 | 2 | 2 | ZSC10_MOUSE | Zscan10 | 3.42 |
| 5 | 7 | Q6NZQ2_MOUSE | Ddx31 | 2.33 | 3 | 3 | Q6NZQ2_MOUSE | Ddx31 | 3.21 |
| 5 | 7 | PWP2_MOUSE | Pwp2 | 3.25 | 4 | 4 | PWP2_MOUSE | Pwp2 | 3.03 |
| 5 | 7 | NOP2_MOUSE | Nop2 | 3.63 | 4 | 4 | NOP2_MOUSE | Nop2 | 3.46 |
| 5 | 7 | J3QMI2_MOUSE | Zfp799 | 3.06 | 5 | 5 | J3QMI2_MOUSE | Zfp799 | 2.95 |
| 5 | 7 | UBQL4_MOUSE | Ubqln4 | 3.88 | 6 | 8 | UBQL4_MOUSE | Ubqln4 | 4.2 |
| 5 | 7 | PPIL4_MOUSE | Ppil4 | 4.04 | 6 | 9 | PPIL4_MOUSE | Ppil4 | 3.35 |
| 5 | 7 | E9Q5G3_MOUSE | Kif23 | 3.32 | 7 | 7 | E9Q5G3_MOUSE | Kif23 | 3.1 |
| 5 | 7 | ZFP60_MOUSE | Zfp60 | 2.95 | 7 | 8 | ZFP60_MOUSE | Zfp60 | 2.89 |
| 5 | 7 | B8JJZ4_MOUSE | Zfp808 | 3.04 | 7 | 9 | B8JJZ4_MOUSE | Zfp808 | 2.76 |
| 5 | 7 | INT9_MOUSE | Ints9 | 3.53 | 8 | 12 | INT9_MOUSE | Ints9 | 3.17 |
| 5 | 7 | A1AT1_MOUSE | Serpina1a | 3.47 | 4 | 7 | A1AT1_MOUSE | Serpina1a | 3.08 |
| 5 | 6 | HIC2_MOUSE | Hic2 | 2.42 | 3 | 3 | HIC2_MOUSE | Hic2 | 2.78 |
| 5 | 6 | ARP5_MOUSE | Actr5 | 4.77 | 5 | 6 | ARP5_MOUSE | Actr5 | 4.41 |
| 5 | 6 | UTP6_MOUSE | Utp6 | 3.29 | 6 | 9 | UTP6_MOUSE | Utp6 | 3.47 |
| 5 | 6 | Q80XR7_MOUSE | Zfp955a | 2.92 | 6 | 6 | Q80XR7_MOUSE | Zfp955a | 2.73 |
| 5 | 6 | RFX2_MOUSE | Rfx2 | 3.56 | 6 | 9 | RFX2_MOUSE | Rfx2 | 4.12 |
| 5 | 6 | GNL3L_MOUSE | Gnl3l | 3.08 | 6 | 7 | GNL3L_MOUSE | Gnl3l | 3.22 |
| 5 | 6 | DDX50_MOUSE | Ddx50 | 2.95 | 11 | 11 | DDX50_MOUSE | Ddx50 | 2.71 |
| 5 | 6 | A2AUY4_MOUSE | Baz2b | 2.78 | 1 | 1 | A2AUY4_MOUSE | Baz2b | 3.48 |
| 5 | 5 | IF2B2_MOUSE | Igf2bp2 | 2.9 | 1 | 1 | IF2B2_MOUSE | Igf2bp2 | 2.74 |
| 5 | 5 | G3UY09_MOUSE | Zfp141 | 2.15 | 1 | 1 | G3UY09_MOUSE | Zfp141 | 2.46 |
| 5 | 5 | API5_MOUSE | Api5 | 3.24 | 1 | 2 | API5_MOUSE | Api5 | 3.16 |
| 5 | 5 | H4_MOUSE | Hist1h4a | 2.52 | 3 | 3 | H4_MOUSE | Hist1h4a | 2.55 |
| 5 | 5 | SHOC2_MOUSE | Shoc2 | 3.16 | 3 | 3 | SHOC2_MOUSE | Shoc2 | 3.13 |
| 5 | 5 | ZN281_MOUSE | Znf281 | 3.93 | 3 | 4 | ZN281_MOUSE | Znf281 | 4.78 |
| 5 | 5 | VPS51_MOUSE | Vps51 | 2.8 | 3 | 3 | VPS51_MOUSE | Vps51 | 2.95 |
| 5 | 5 | RIOK2_MOUSE | Riok2 | 3.35 | 3 | 3 | RIOK2_MOUSE | Riok2 | 3.54 |
| 5 | 5 | RNF12_MOUSE | Rlim | 2.97 | 4 | 4 | RNF12_MOUSE | Rlim | 3.44 |
| 5 | 5 | MTBP_MOUSE | Mtbp | 2.78 | 5 | 5 | MTBP_MOUSE | Mtbp | 3.47 |
| 5 | 5 | RBBP5_MOUSE | Rbbp5 | 3.4 | 6 | 7 | RBBP5_MOUSE | Rbbp5 | 3.24 |
| 5 | 5 | PESC_MOUSE | Pes1 | 3.48 | 6 | 6 | PESC_MOUSE | Pes1 | 3.7 |
| 5 | 5 | MRE11_MOUSE | Mre11a | 3.21 | 8 | 8 | MRE11_MOUSE | Mre11a | 3.94 |
| 5 | 5 | DNM3A_MOUSE | Dnmt3a | 2.88 | 8 | 9 | DNM3A_MOUSE | Dnmt3a | 2.77 |
| 5 | 5 | Q3TFZ4_MOUSE | Zfp7 | 3.96 | 8 | 9 | Q3TFZ4_MOUSE | Zfp7 | 3.29 |
| 5 | 5 | MCM4_MOUSE | Mcm4 | 2.91 | 9 | 10 | MCM4_MOUSE | Mcm4 | 3.53 |
| 5 | 5 | MCM5_MOUSE | Mcm5 | 3.17 | 10 | 11 | MCM5_MOUSE | Mcm5 | 2.96 |
| 5 | 5 | TBL3_MOUSE | Tbl3 | 3.4 | 12 | 14 | TBL3_MOUSE | Tbl3 | 3.61 |
| 5 | 5 | MLL4_MOUSE | Wbp7 | 2.69 | 11 | 12 | MLL4_MOUSE | Wbp7 | 2.83 |
| 5 | 5 | SPAG5_MOUSE | Spag5 | 3.28 | 8 | 9 | SPAG5_MOUSE | Spag5 | 3.92 |
| 5 | 5 | NU155_MOUSE | Nup155 | 3.94 | 7 | 7 | NU155_MOUSE | Nup155 | 3.82 |
| 5 | 5 | G5E8B9_MOUSE | Rpl24 | 2.65 | 6 | 7 | G5E8B9_MOUSE | Rpl24 | 3.01 |
| 5 | 5 | CENPE_MOUSE | Cenpe | 3.42 | 6 | 6 | CENPE_MOUSE | Cenpe | 2.79 |
| 5 | 5 | BAZ1A_MOUSE | Baz1a | 3.25 | 4 | 5 | BAZ1A_MOUSE | Baz1a | 2.85 |
| 5 | 5 | TANC1_MOUSE | Tanc1 | 2.94 | 5 | 5 | TANC1_MOUSE | Tanc1 | 2.49 |
| 5 | 5 | ZFR_MOUSE | Zfr | 2.87 | 4 | 4 | ZFR_MOUSE | Zfr | 3.21 |
| 5 | 5 | Q7TSH6_MOUSE | Scaf4 | 2.86 | 3 | 4 | Q7TSH6_MOUSE | Scaf4 | 3.06 |
| 5 | 5 | TOPRS_MOUSE | Topors | 2.7 | 4 | 4 | TOPRS_MOUSE | Topors | 2.83 |
| 5 | 5 | DHX30_MOUSE | Dhx30 | 2.91 | 3 | 3 | DHX30_MOUSE | Dhx30 | 3.11 |
| 5 | 5 | ZN532_MOUSE | Znf532 | 3.57 | 2 | 2 | ZN532_MOUSE | Znf532 | 3.13 |
| 5 | 5 | ZFP26_MOUSE | Zfp26 | 2.85 | 2 | 2 | ZFP26_MOUSE | Zfp26 | 2.74 |
| 5 | 5 | PHIP_MOUSE | Phip | 2.96 | 2 | 2 | PHIP_MOUSE | Phip | 3.58 |
| 5 | 5 | ATAD2_MOUSE | Atad2 | 3.74 | 0 | 0 | na | na | 0 |
| 4 | 8 | FUS_MOUSE | Fus | 3.65 | 5 | 7 | FUS_MOUSE | Fus | 3.41 |
| 4 | 7 | HS90B_MOUSE | Hsp90ab1 | 3.27 | 4 | 6 | HS90B_MOUSE | Hsp90ab1 | 3.82 |
| 4 | 7 | B0V2W5_MOUSE | Zfp472 | 4.41 | 4 | 5 | B0V2W5_MOUSE | Zfp472 | 4.8 |
| 4 | 6 | PIAS4_MOUSE | Pias4 | 3.87 | 2 | 4 | PIAS4_MOUSE | Pias4 | 4.24 |
| 4 | 6 | RSRC2_MOUSE | Rsrc2 | 3.5 | 3 | 4 | RSRC2_MOUSE | Rsrc2 | 2.69 |
| 4 | 6 | TYY1_MOUSE | Yy1 | 2.98 | 3 | 4 | TYY1_MOUSE | Yy1 | 2.32 |
| 4 | 6 | EXOS9_MOUSE | Exosc9 | 3.38 | 4 | 5 | EXOS9_MOUSE | Exosc9 | 3.62 |
| 4 | 6 | MTA3_MOUSE | Mta3 | 3.91 | 4 | 5 | MTA3_MOUSE | Mta3 | 3.31 |
| 4 | 6 | A2A8V7_MOUSE | Gm13247 | 2.94 | 6 | 14 | A2A8V7_MOUSE | Gm13247 | 3.32 |
| 4 | 5 | U2AF2_MOUSE | U2af2 | 3.54 | 1 | 2 | U2AF2_MOUSE | U2af2 | 3.56 |
| 4 | 5 | Q8BFS8_MOUSE | Zfp418 | 2.42 | 2 | 2 | Q8BFS8_MOUSE | Zfp418 | 2.79 |
| 4 | 5 | CTNA1_MOUSE | Ctnna1 | 2.69 | 2 | 2 | CTNA1_MOUSE | Ctnna1 | 2.61 |
| 4 | 5 | DDX27_MOUSE | Ddx27 | 3.32 | 3 | 3 | DDX27_MOUSE | Ddx27 | 3.58 |
| 4 | 5 | SREK1_MOUSE | Srek1 | 3.53 | 3 | 6 | SREK1_MOUSE | Srek1 | 3.3 |
| 4 | 5 | VRTN_MOUSE | Vrtn | 2.97 | 3 | 3 | VRTN_MOUSE | Vrtn | 2.85 |
| 4 | 5 | TRI41_MOUSE | Trim41 | 3.53 | 4 | 4 | TRI41_MOUSE | Trim41 | 2.95 |
| 4 | 5 | SLU7_MOUSE | Slu7 | 3.28 | 5 | 5 | SLU7_MOUSE | Slu7 | 4.01 |
| 4 | 5 | WDR46_MOUSE | Wdr46 | 3.64 | 5 | 7 | WDR46_MOUSE | Wdr46 | 4.12 |
| 4 | 5 | ORC2_MOUSE | Orc2 | 3.04 | 6 | 6 | ORC2_MOUSE | Orc2 | 3.22 |
| 4 | 5 | CWC22_MOUSE | Cwc22 | 2.87 | 4 | 6 | CWC22_MOUSE | Cwc22 | 3.42 |
| 4 | 5 | E9PZM7_MOUSE | Scaf11 | 2.86 | 5 | 5 | E9PZM7_MOUSE | Scaf11 | 3.38 |
| 4 | 5 | CASZ1_MOUSE | Casz1 | 2.94 | 1 | 1 | CASZ1_MOUSE | Casz1 | 2.31 |
| 4 | 4 | ANC2_MOUSE | Anapc2 | 3.25 | 0 | 0 | na | na | 0 |
| 4 | 4 | TCPA_MOUSE | Tcp1 | 2.85 | 0 | 0 | na | na | 0 |
| 4 | 4 | INCE_MOUSE | Incenp | 3.47 | 1 | 1 | INCE_MOUSE | Incenp | 4.28 |
| 4 | 4 | HRG_MOUSE | Hrg | 2.79 | 1 | 1 | HRG_MOUSE | Hrg | 3.46 |
| 4 | 4 | PSPC1_MOUSE | Pspc1 | 2.72 | 1 | 1 | PSPC1_MOUSE | Pspc1 | 2.62 |
| 4 | 4 | SGOL2_MOUSE | Sgol2 | 2.67 | 2 | 2 | SGOL2_MOUSE | Sgol2 | 3.82 |
| 4 | 4 | GTPB1_MOUSE | Gtpbp1 | 3.59 | 2 | 2 | GTPB1_MOUSE | Gtpbp1 | 4.04 |
| 4 | 4 | CDC6_MOUSE | Cdc6 | 2.53 | 2 | 2 | CDC6_MOUSE | Cdc6 | 2.57 |
| 4 | 4 | PR38B_MOUSE | Prpf38b | 2.64 | 2 | 2 | PR38B_MOUSE | Prpf38b | 2.84 |
| 4 | 4 | N42L2_MOUSE | N4bp2l2 | 2.51 | 2 | 2 | N42L2_MOUSE | N4bp2l2 | 2.56 |
| 4 | 4 | GTF2I_MOUSE | Gtf2i | 2.28 | 3 | 3 | GTF2I_MOUSE | Gtf2i | 3.29 |
| 4 | 4 | 3BP1_MOUSE | Sh3bp1 | 2.93 | 3 | 3 | 3BP1_MOUSE | Sh3bp1 | 2.82 |
| 4 | 4 | EXOC2_MOUSE | Exoc2 | 3.13 | 3 | 3 | EXOC2_MOUSE | Exoc2 | 3.42 |
| 4 | 4 | VPS53_MOUSE | Vps53 | 3.34 | 3 | 3 | VPS53_MOUSE | Vps53 | 3.35 |
| 4 | 4 | RGAP1_MOUSE | Racgap1 | 2.85 | 3 | 3 | RGAP1_MOUSE | Racgap1 | 2.82 |
| 4 | 4 | IGHM_MOUSE | Igh-6 | 3.98 | 3 | 4 | IGHM_MOUSE | Igh-6 | 3.54 |
| 4 | 4 | HMMR_MOUSE | Hmmr | 2.56 | 4 | 4 | HMMR_MOUSE | Hmmr | 3.34 |
| 4 | 4 | EF2_MOUSE | Eef2 | 2.38 | 4 | 4 | EF2_MOUSE | Eef2 | 2.85 |
| 4 | 4 | NOL11_MOUSE | Nol11 | 2.62 | 4 | 4 | NOL11_MOUSE | Nol11 | 2.64 |
| 4 | 4 | VPS52_MOUSE | Vps52 | 2.98 | 4 | 4 | VPS52_MOUSE | Vps52 | 2.74 |
| 4 | 4 | Q6IQX8_MOUSE | Zfp219 | 3.22 | 5 | 5 | Q6IQX8_MOUSE | Zfp219 | 3.64 |
| 4 | 4 | GRP75_MOUSE | Hspa9 | 3.08 | 5 | 5 | GRP75_MOUSE | Hspa9 | 2.98 |
| 4 | 4 | AATF_MOUSE | Aatf | 4.29 | 5 | 5 | AATF_MOUSE | Aatf | 3.47 |
| 4 | 4 | CPSF6_MOUSE | Cpsf6 | 3.55 | 5 | 6 | CPSF6_MOUSE | Cpsf6 | 3.76 |

| WT | | | | | Dnmt1-null | | | | |
|---|---|---|---|---|---|---|---|---|---|
| Uniq | Tota | reference | Gene Syn | AVG | Uniq | Tota | reference | Gene Syn | AVG |
| 4 | 4 | BRD9_MOUSE | Brd9 | 3.62 | 5 | 6 | BRD9_MOUSE | Brd9 | 2.99 |
| 4 | 4 | Q6DFU8_MOUSE | Zfp948 | 2.28 | 5 | 5 | Q6DFU8_MOUSE | Zfp948 | 2.25 |
| 4 | 4 | CNOTA_MOUSE | Cnot10 | 4.07 | 6 | 6 | CNOTA_MOUSE | Cnot10 | 3.6 |
| 4 | 4 | LRWD1_MOUSE | LRWD1 | 3.08 | 6 | 6 | LRWD1_MOUSE | LRWD1 | 3.44 |
| 4 | 4 | SAS10_MOUSE | Utp3 | 3.54 | 6 | 8 | SAS10_MOUSE | Utp3 | 3.94 |
| 4 | 4 | DIAP3_MOUSE | Diaph3 | 3.27 | 11 | 14 | DIAP3_MOUSE | Diaph3 | 3.53 |
| 4 | 4 | NCOR1_MOUSE | Ncor1 | 3.33 | 10 | 11 | NCOR1_MOUSE | Ncor1 | 3.28 |
| 4 | 4 | GCFC1_MOUSE | Gcfc1 | 3.88 | 7 | 9 | GCFC1_MOUSE | Gcfc1 | 3.17 |
| 4 | 4 | TOPB1_MOUSE | Topbp1 | 3.18 | 6 | 7 | TOPB1_MOUSE | Topbp1 | 3.67 |
| 4 | 4 | PDS5A_MOUSE | Pds5a | 3.34 | 7 | 7 | PDS5A_MOUSE | Pds5a | 3.92 |
| 4 | 4 | SRRT_MOUSE | Srrt | 3.53 | 6 | 7 | SRRT_MOUSE | Srrt | 3.38 |
| 4 | 4 | CHAP1_MOUSE | Champ1 | 2.57 | 5 | 6 | CHAP1_MOUSE | Champ1 | 2.62 |
| 4 | 4 | BRCA1_MOUSE | Brca1 | 2.93 | 6 | 6 | BRCA1_MOUSE | Brca1 | 3.2 |
| 4 | 4 | MOR2A_MOUSE | Morc2a | 2.67 | 5 | 6 | MOR2A_MOUSE | Morc2a | 2.54 |
| 4 | 4 | ELP1_MOUSE | Ikbkap | 3.76 | 5 | 5 | ELP1_MOUSE | Ikbkap | 3.38 |
| 4 | 4 | ZO1_MOUSE | Tjp1 | 3.14 | 5 | 5 | ZO1_MOUSE | Tjp1 | 2.69 |
| 4 | 4 | ZF106_MOUSE | Zfp106 | 2.57 | 5 | 5 | ZF106_MOUSE | Zfp106 | 2.72 |
| 4 | 4 | BCOR_MOUSE | Bcor | 3.9 | 4 | 4 | BCOR_MOUSE | Bcor | 4.37 |
| 4 | 4 | E9Q4F8_MOUSE | Ankrd11 | 3.29 | 3 | 3 | E9Q4F8_MOUSE | Ankrd11 | 2.14 |
| 4 | 4 | CNDD3_MOUSE | Ncapd3 | 3.75 | 3 | 3 | CNDD3_MOUSE | Ncapd3 | 3.75 |
| 4 | 4 | E9QAP7_MOUSE | Taf4a | 4.28 | 2 | 2 | E9QAP7_MOUSE | Taf4a | 4.03 |
| 4 | 4 | DPOE1_MOUSE | Pole | 2.88 | 1 | 1 | DPOE1_MOUSE | Pole | 2.58 |
| 4 | 4 | B2RT41_MOUSE | Zfc3h1 | 2.78 | 0 | 0 | na | na | 0 |
| 4 | 4 | NU188_MOUSE | Nup188 | 2.86 | 0 | 0 | na | na | 0 |
| 4 | 4 | ASCC3_MOUSE | Ascc3 | 2.77 | 0 | 0 | na | na | 0 |
| 4 | 4 | IQGA1_MOUSE | Iqgap1 | 2.25 | 0 | 0 | na | na | 0 |
| 3 | 9 | Q3UIX4_MOUSE | Srsf11 | 2.47 | 4 | 15 | Q3UIX4_MOUSE | Srsf11 | 2.81 |
| 3 | 7 | DDX3X_MOUSE | Ddx3x | 2.8 | 4 | 5 | DDX3X_MOUSE | Ddx3x | 2.79 |
| 3 | 6 | B1AZK8_MOUSE | Gm9631 | 2.78 | 3 | 6 | B1AZK8_MOUSE | Gm9631 | 3.13 |
| 3 | 5 | Q8K0G9_MOUSE | Zfp119b | 2.96 | 1 | 2 | Q8K0G9_MOUSE | Zfp119b | 3.2 |
| 3 | 5 | NELFA_MOUSE | Whsc2 | 3.53 | 4 | 4 | NELFA_MOUSE | Whsc2 | 3.71 |
| 3 | 5 | CCNK_MOUSE | Ccnk | 3.74 | 6 | 6 | CCNK_MOUSE | Ccnk | 3.71 |
| 3 | 4 | PPM1D_MOUSE | Ppm1d | 2.55 | 0 | 0 | na | na | 0 |
| 3 | 4 | ZNF48_MOUSE | Znf48 | 2.76 | 0 | 0 | na | na | 0 |
| 3 | 4 | F8VQC1_MOUSE | Srp72 | 3.66 | 0 | 0 | na | na | 0 |
| 3 | 4 | RFA1_MOUSE | Rpa1 | 2.57 | 1 | 1 | RFA1_MOUSE | Rpa1 | 2.89 |
| 3 | 4 | G5E8C8_MOUSE | Zfp612 | 2.27 | 1 | 1 | G5E8C8_MOUSE | Zfp612 | 2.5 |
| 3 | 4 | AKAP8_MOUSE | Akap8 | 2.85 | 1 | 2 | AKAP8_MOUSE | Akap8 | 3.8 |
| 3 | 4 | DYN1_MOUSE | Dnm1 | 3.18 | 2 | 2 | DYN1_MOUSE | Dnm1 | 2.64 |
| 3 | 4 | ARFG2_MOUSE | Arfgap2 | 2.43 | 2 | 3 | ARFG2_MOUSE | Arfgap2 | 3.71 |
| 3 | 4 | IMA1_MOUSE | Kpna1 | 3.38 | 2 | 3 | IMA1_MOUSE | Kpna1 | 3.29 |
| 3 | 4 | E9Q981_MOUSE | 9830147E | 2.48 | 2 | 3 | E9Q981_MOUSE | 9830147E | 2.96 |
| 3 | 4 | MARK2_MOUSE | Mark2 | 2.83 | 2 | 2 | MARK2_MOUSE | Mark2 | 2.97 |
| 3 | 4 | ASH2L_MOUSE | Ash2l | 3.94 | 3 | 4 | ASH2L_MOUSE | Ash2l | 4.18 |
| 3 | 4 | BRD7_MOUSE | Brd7 | 3.86 | 3 | 3 | BRD7_MOUSE | Brd7 | 3.46 |
| 3 | 4 | AK17B_MOUSE | Akap17b | 2.86 | 3 | 4 | AK17B_MOUSE | Akap17b | 2.8 |
| 3 | 4 | TCF20_MOUSE | Tcf20 | 2.53 | 3 | 3 | TCF20_MOUSE | Tcf20 | 2.97 |
| 3 | 4 | PUM1_MOUSE | Pum1 | 2.49 | 4 | 4 | PUM1_MOUSE | Pum1 | 2.71 |
| 3 | 4 | TOX3_MOUSE | Tox3 | 4.32 | 4 | 5 | TOX3_MOUSE | Tox3 | 4.21 |
| 3 | 4 | RBM40_MOUSE | Rnpc3 | 2.8 | 4 | 4 | RBM40_MOUSE | Rnpc3 | 3.09 |
| 3 | 4 | B2RPU8_MOUSE | 2410018M | 2.93 | 4 | 4 | B2RPU8_MOUSE | 2410018M | 2.92 |
| 3 | 4 | PSIP1_MOUSE | Psip1 | 3.19 | 5 | 8 | PSIP1_MOUSE | Psip1 | 3.7 |
| 3 | 4 | NXF1_MOUSE | Nxf1 | 3.17 | 6 | 7 | NXF1_MOUSE | Nxf1 | 3.75 |
| 3 | 4 | MPP8_MOUSE | Mphosph8 | 2.66 | 7 | 9 | MPP8_MOUSE | Mphosph8 | 3.8 |
| 3 | 4 | UBP26_MOUSE | Usp26 | 3.23 | 7 | 8 | UBP26_MOUSE | Usp26 | 3.21 |
| 3 | 4 | BAZ1B_MOUSE | Baz1b | 2.92 | 8 | 9 | BAZ1B_MOUSE | Baz1b | 2.87 |
| 3 | 4 | YETS2_MOUSE | Yeats2 | 3.17 | 6 | 6 | YETS2_MOUSE | Yeats2 | 2.34 |
| 3 | 4 | NU214_MOUSE | Nup214 | 3.21 | 4 | 4 | NU214_MOUSE | Nup214 | 2.74 |
| 3 | 4 | SIN3B_MOUSE | Sin3b | 2.86 | 2 | 2 | SIN3B_MOUSE | Sin3b | 3.1 |
| 3 | 4 | IGH1M_MOUSE | Ighg1 | 2.84 | 1 | 1 | IGH1M_MOUSE | Ighg1 | 3.3 |
| 3 | 4 | MUG1_MOUSE | Mug1 | 3.3 | 1 | 1 | MUG1_MOUSE | Mug1 | 2.97 |
| 3 | 4 | TAF1_MOUSE | Taf1 | 2.93 | 0 | 0 | na | na | 0 |
| 3 | 3 | ZN250_MOUSE | Znf250 | 2.67 | 3 | 4 | ZN250_MOUSE | Znf250 | 3.07 |
| 3 | 3 | COIL_MOUSE | Coil | 4.04 | 3 | 4 | COIL_MOUSE | Coil | 4.13 |
| 3 | 3 | SP2_MOUSE | Sp2 | 4.02 | 3 | 4 | SP2_MOUSE | Sp2 | 3.58 |
| 3 | 3 | EXOC5_MOUSE | Exoc5 | 2.85 | 3 | 3 | EXOC5_MOUSE | Exoc5 | 3.65 |
| 3 | 3 | E9QAG4_MOUSE | 4930522L | 2.11 | 4 | 4 | E9QAG4_MOUSE | 4930522L | 2.8 |
| 3 | 3 | LIN54_MOUSE | Lin54 | 2.84 | 4 | 4 | LIN54_MOUSE | Lin54 | 2.87 |
| 3 | 3 | Q91WM0_MOUSE | Zfp617 | 2.68 | 4 | 4 | Q91WM0_MOUSE | Zfp617 | 2.41 |
| 3 | 3 | MET16_MOUSE | Mettl16 | 3.56 | 4 | 4 | MET16_MOUSE | Mettl16 | 3.5 |
| 3 | 3 | RFX1_MOUSE | Rfx1 | 2.86 | 4 | 4 | RFX1_MOUSE | Rfx1 | 3.35 |
| 3 | 3 | SNUT1_MOUSE | Sart1 | 3.65 | 4 | 4 | SNUT1_MOUSE | Sart1 | 2.93 |
| 3 | 3 | PO2F1_MOUSE | Pou2f1 | 3.66 | 5 | 5 | PO2F1_MOUSE | Pou2f1 | 3.88 |
| 3 | 3 | NOL8_MOUSE | Nol8 | 3.89 | 5 | 5 | NOL8_MOUSE | Nol8 | 3.6 |
| 3 | 3 | SIX4_MOUSE | Six4 | 2.77 | 6 | 6 | SIX4_MOUSE | Six4 | 3.14 |
| 3 | 3 | TAF5_MOUSE | Taf5 | 3.33 | 6 | 7 | TAF5_MOUSE | Taf5 | 3.75 |
| 3 | 3 | TDIF2_MOUSE | Dnttip2 | 3.17 | 6 | 6 | TDIF2_MOUSE | Dnttip2 | 3.15 |
| 3 | 3 | DDX54_MOUSE | Ddx54 | 2.79 | 6 | 7 | DDX54_MOUSE | Ddx54 | 3.65 |
| 3 | 3 | LAP2A_MOUSE | Tmpo | 3.39 | 10 | 11 | LAP2A_MOUSE | Tmpo | 3.53 |
| 3 | 3 | DNLI1_MOUSE | Lig1 | 3.73 | 9 | 11 | DNLI1_MOUSE | Lig1 | 3.31 |
| 3 | 3 | NCOR2_MOUSE | Ncor2 | 3.85 | 8 | 8 | NCOR2_MOUSE | Ncor2 | 3.36 |
| 3 | 3 | E9Q5F9_MOUSE | Setd2 | 3.49 | 5 | 6 | E9Q5F9_MOUSE | Setd2 | 3.1 |
| 3 | 3 | RREB1_MOUSE | Rreb1 | 4.23 | 5 | 6 | RREB1_MOUSE | Rreb1 | 3.74 |
| 3 | 3 | MLL1_MOUSE | Mll | 3.18 | 5 | 5 | MLL1_MOUSE | Mll | 3.4 |
| 3 | 3 | ESF1_MOUSE | Esf1 | 2.4 | 4 | 4 | ESF1_MOUSE | Esf1 | 2.39 |
| 2 | 10 | A2AWF2_MOUSE | Rex2 | 3.77 | 2 | 8 | A2AWF2_MOUSE | Rex2 | 4.1 |
| 2 | 5 | E9Q5C9_MOUSE | Nolc1 | 2.25 | 5 | 6 | E9Q5C9_MOUSE | Nolc1 | 2.32 |
| 2 | 4 | DGC14_MOUSE | Dgcr14 | 4.64 | 0 | 0 | na | na | 0 |
| 2 | 3 | H3BLB0_MOUSE | Gm13150 | 3.22 | 2 | 4 | H3BLB0_MOUSE | Gm13150 | 2.98 |
| 2 | 3 | E9Q3R6_MOUSE | Zfp169 | 3.35 | 3 | 4 | E9Q3R6_MOUSE | Zfp169 | 3.06 |
| 2 | 3 | RXRB_MOUSE | Rxrb | 3.63 | 4 | 6 | RXRB_MOUSE | Rxrb | 3.15 |
| 2 | 3 | SFR19_MOUSE | Scaf1 | 3.8 | 8 | 8 | SFR19_MOUSE | Scaf1 | 3.14 |
| 2 | 2 | BBX_MOUSE | Bbx | 1.93 | 3 | 4 | BBX_MOUSE | Bbx | 2.35 |
| 2 | 2 | Q8BGV5_MOUSE | Zfp128 | 2.58 | 3 | 3 | Q8BGV5_MOUSE | Zfp128 | 3.03 |
| 2 | 2 | A2ART0_MOUSE | OTTMUS | 2.86 | 3 | 3 | A2ART0_MOUSE | OTTMUS | 2.42 |
| 2 | 2 | CSTFT_MOUSE | Cstf2t | 3.36 | 3 | 3 | CSTFT_MOUSE | Cstf2t | 2.92 |
| 2 | 2 | KDM2A_MOUSE | Kdm2a | 3.31 | 3 | 3 | KDM2A_MOUSE | Kdm2a | 3.63 |
| 2 | 2 | ZN710_MOUSE | Znf710 | 3.45 | 3 | 5 | ZN710_MOUSE | Znf710 | 4.02 |
| 2 | 2 | FUBP2_MOUSE | Khsrp | 3.26 | 3 | 3 | FUBP2_MOUSE | Khsrp | 2.53 |
| 2 | 2 | UBIP1_MOUSE | Ubp1 | 2.92 | 3 | 5 | UBIP1_MOUSE | Ubp1 | 2.88 |
| 2 | 2 | MED17_MOUSE | Med17 | 2.78 | 3 | 3 | MED17_MOUSE | Med17 | 3.06 |
| 2 | 2 | NOP9_MOUSE | Nop9 | 3.34 | 3 | 3 | NOP9_MOUSE | Nop9 | 3.06 |
| 2 | 2 | FIBG_MOUSE | Fgg | 2.39 | 3 | 4 | FIBG_MOUSE | Fgg | 3.45 |
| 2 | 2 | UBQL1_MOUSE | Ubqln1 | 3.54 | 3 | 3 | UBQL1_MOUSE | Ubqln1 | 2.89 |
| 2 | 2 | MEN1_MOUSE | Men1 | 2.87 | 3 | 3 | MEN1_MOUSE | Men1 | 2.94 |
| 2 | 2 | SENP1_MOUSE | Senp1 | 2.62 | 3 | 3 | SENP1_MOUSE | Senp1 | 3.3 |
| 2 | 2 | A2A7I1_MOUSE | Gm13145 | 4.47 | 4 | 7 | A2A7I1_MOUSE | Gm13145 | 4.27 |
| 2 | 2 | WDR76_MOUSE | Wdr76 | 2.66 | 4 | 6 | WDR76_MOUSE | Wdr76 | 2.82 |
| 2 | 2 | HAUS5_MOUSE | Haus5 | 2.83 | 4 | 4 | HAUS5_MOUSE | Haus5 | 2.93 |
| 2 | 2 | HAUS3_MOUSE | Haus3 | 3.59 | 4 | 5 | HAUS3_MOUSE | Haus3 | 3.68 |
| 2 | 2 | EIF3D_MOUSE | Eif3d | 3.48 | 4 | 4 | EIF3D_MOUSE | Eif3d | 3.65 |
| 2 | 2 | A2ARX0_MOUSE | Gm14443 | 2.98 | 4 | 5 | A2ARX0_MOUSE | Gm14443 | 2.58 |
| 2 | 2 | MYBB_MOUSE | Mybl2 | 2.79 | 4 | 6 | MYBB_MOUSE | Mybl2 | 2.9 |
| 2 | 2 | RPN1_MOUSE | Rpn1 | 3.1 | 4 | 5 | RPN1_MOUSE | Rpn1 | 3.07 |
| 2 | 2 | NRF1_MOUSE | Nrf1 | 2.68 | 5 | 5 | NRF1_MOUSE | Nrf1 | 2.94 |
| 2 | 2 | SCYL2_MOUSE | Scyl2 | 2.82 | 5 | 5 | SCYL2_MOUSE | Scyl2 | 3.45 |
| 2 | 2 | Q80XN4_MOUSE | Zfp871 | 2.49 | 5 | 6 | Q80XN4_MOUSE | Zfp871 | 2.93 |
| 2 | 2 | EXC6B_MOUSE | Exoc6b | 2.78 | 5 | 5 | EXC6B_MOUSE | Exoc6b | 3.27 |
| 2 | 2 | RRMJ3_MOUSE | Ftsj3 | 3.78 | 6 | 6 | RRMJ3_MOUSE | Ftsj3 | 3.18 |
| 2 | 2 | Z280C_MOUSE | Znf280c | 3.22 | 7 | 7 | Z280C_MOUSE | Znf280c | 2.89 |
| 2 | 2 | LMNA_MOUSE | Lmna | 3.39 | 9 | 11 | LMNA_MOUSE | Lmna | 3.09 |
| 2 | 2 | TTK_MOUSE | Ttk | 3.31 | 11 | 13 | TTK_MOUSE | Ttk | 3.53 |
| 2 | 2 | GT2D1_MOUSE | Gtf2ird1 | 3.24 | 7 | 7 | GT2D1_MOUSE | Gtf2ird1 | 2.72 |
| 2 | 2 | GPTC1_MOUSE | Gpatch1 | 2.77 | 5 | 5 | GPTC1_MOUSE | Gpatch1 | 3.76 |
| 2 | 2 | PHC1_MOUSE | Phc1 | 3.74 | 4 | 5 | PHC1_MOUSE | Phc1 | 3.97 |
| 2 | 2 | BLM_MOUSE | Blm | 3.61 | 5 | 5 | BLM_MOUSE | Blm | 3.51 |
| 2 | 2 | Q6XZL8_MOUSE | Dot1l | 2.19 | 5 | 5 | Q6XZL8_MOUSE | Dot1l | 2.64 |
| 2 | 2 | UBN2_MOUSE | Ubn2 | 2.95 | 3 | 4 | UBN2_MOUSE | Ubn2 | 2.92 |
| 2 | 2 | MCM2_MOUSE | Mcm2 | 2.94 | 4 | 4 | MCM2_MOUSE | Mcm2 | 2.77 |
| 1 | 5 | Q3UTQ6_MOUSE | Zfp868 | 3.41 | 1 | 2 | Q3UTQ6_MOUSE | Zfp868 | 3.31 |
| 1 | 4 | B1ASQ7_MOUSE | Gm13212 | 3.89 | 0 | 0 | na | na | 0 |
| 1 | 4 | K2C1_MOUSE | Krt1 | 2.56 | 1 | 4 | K2C1_MOUSE | Krt1 | 2.91 |
| 1 | 2 | SP1_MOUSE | Sp1 | 5.92 | 2 | 4 | SP1_MOUSE | Sp1 | 4.83 |
| 1 | 2 | EXOC8_MOUSE | Exoc8 | 3.73 | 4 | 4 | EXOC8_MOUSE | Exoc8 | 2.79 |
| 1 | 2 | ARP8_MOUSE | Actr8 | 4.52 | 4 | 4 | ARP8_MOUSE | Actr8 | 2.56 |
| 1 | 1 | CDC45_MOUSE | Cdc45 | 4.14 | 3 | 4 | CDC45_MOUSE | Cdc45 | 2.84 |
| 1 | 1 | Q6PFD9_MOUSE | Nup98 | 4.69 | 4 | 4 | Q6PFD9_MOUSE | Nup98 | 2.75 |
| 1 | 1 | HP1B3_MOUSE | Hp1bp3 | 3.15 | 4 | 4 | HP1B3_MOUSE | Hp1bp3 | 3.14 |
| 1 | 1 | ZN148_MOUSE | Znf148 | 3.49 | 5 | 6 | ZN148_MOUSE | Znf148 | 3.13 |
| 1 | 1 | ZKSC3_MOUSE | Zkscan3 | 2.76 | 10 | 11 | ZKSC3_MOUSE | Zkscan3 | 3.11 |
| 1 | 1 | E9PYL2_MOUSE | Prr12 | 3.79 | 6 | 6 | E9PYL2_MOUSE | Prr12 | 3.94 |
| 1 | 1 | UBP2L_MOUSE | Ubap2l | 2.7 | 4 | 4 | UBP2L_MOUSE | Ubap2l | 3.57 |
| 1 | 1 | C8AP2_MOUSE | Casp8ap2 | 3.93 | 4 | 4 | C8AP2_MOUSE | Casp8ap2 | 3 |
| 1 | 1 | PCNT_MOUSE | Pcnt | 2.65 | 4 | 4 | PCNT_MOUSE | Pcnt | 2.78 |
| 0 | 0 | na | na | 0 | 3 | 4 | ATRIP_MOUSE | Atrip | 3.52 |
| 0 | 0 | na | na | 0 | 4 | 4 | E9Q1A5_MOUSE | Zfp384 | 3.75 |
| 0 | 0 | na | na | 0 | 4 | 4 | UT14A_MOUSE | Utp14a | 2.99 |
| 0 | 0 | na | na | 0 | 4 | 4 | NR2C2_MOUSE | Nr2c2 | 3.1 |
| 0 | 0 | na | na | 0 | 4 | 4 | EPC1_MOUSE | Epc1 | 3.06 |
| 0 | 0 | na | na | 0 | 5 | 6 | ZFP59_MOUSE | Zfp59 | 2.83 |
| 0 | 0 | na | na | 0 | 5 | 5 | CE170_MOUSE | Cep170 | 2.67 |
| 0 | 0 | na | na | 0 | 5 | 5 | B1AVB4_MOUSE | Scml2 | 3.08 |
| 0 | 0 | na | na | 0 | 4 | 4 | BAG6_MOUSE | Bag6 | 2.79 |
| 0 | 0 | na | na | 0 | 4 | 4 | MYPT1_MOUSE | Ppp1r12a | 2.55 |
| 0 | 0 | na | na | 0 | 4 | 4 | E9Q8X5_MOUSE | Zbtb10 | 3.54 |
| 0 | 0 | na | na | 0 | 4 | 4 | CTRO_MOUSE | Cit | 2.13 |

## Table S2: *O*-GlcNAcylated proteins associated with TRIM28 in wild type and *Dnmt1*$^{-/-}$ ES cells.

| Gene Symbol | Tandem IP : TRIM28 followed by WGA | | | | Proteins depleted by more than 2 folds in *D1*$^{-/-}$ VS WT after tandem IP (Unique WT >= 6) | Function | Other name | Protein complex |
|---|---|---|---|---|---|---|---|---|
| | Wild type | | *Dnmt1*$^{-/-}$ | | | | | |
| | Unique | Total | Unique | Total | | | | |
| Rif1 | 13 | 13 | 9 | 9 | | Telomere-associated protein | | |
| Snrnp200 | 8 | 8 | 7 | 7 | | RNA helicase | | |
| **Chd4** | **7** | **8** | **0** | **0** | **CHD4/MI2β** | **Transcriptional regulator** | **Mi-2β** | **NURD** |
| Hcfc1 | 10 | 10 | 15 | 17 | | Interacts with OGT | | OGT |
| Nup98 | 11 | 13 | 13 | 16 | | Nuclear pore complex | | |
| **Cul7** | **16** | **17** | **1** | **1** | **CUL7** | **Regulates the imprinted cluster *H19-Igf2*** | **p185** | **Cul7-RING** |
| Qser1 | 1 | 1 | 18 | 18 | | In complex with RNA PolII | | RNA PolII |
| Smarca4 | 5 | 5 | 2 | 2 | | Transcriptional regulator | Brg1 | SNF2/SMARC5 |
| Top2a | 42 | 49 | 34 | 36 | | DNA topoisomerase II | | |
| **Zmym2** | **16** | **16** | **6** | **6** | **ZMYM2/ZFP198** | **in complex with HDAC1 and LSD1** | **Zfp198** | **LSD1** |
| Mybbp1a | 17 | 17 | 25 | 26 | | Transcriptional regulator | | SNF2/SMARC5 |
| Msh6 | 13 | 13 | 18 | 18 | | DNA mismatch repair | | |
| Dhx9 | 30 | 34 | 23 | 23 | | RNA helicase | | |
| **Sf3b1** | **12** | **12** | **6** | **6** | **SF3B1** | **Essential for Polycomb silencing in mouse** | | **SNF2/SMARCA5** |
| Bms1 | 11 | 11 | 17 | 18 | | Ribosome biogenesis | | |
| Sf3b3 | 27 | 28 | 22 | 22 | | Splicing factor | | |
| Emsy | 10 | 11 | 13 | 13 | | Transcriptional repressor | | HDAC1 |
| Tcof1 | 10 | 10 | 8 | 8 | | Ribosome biogenesis | | |
| Rfc1 | 7 | 7 | 11 | 11 | | DNA replication | | |
| Tcerg1 | 10 | 10 | 16 | 16 | | Interacts with RNA PolII | | RNA PolII |
| Smarca1 | 9 | 10 | 6 | 6 | | Nucleosome remodeling | Snf2l | NURF |
| **Smarca5** | **19** | **20** | **4** | **4** | **SMARCA5/SNF2H** | **Transcriptional repressor** | **Snf2h** | **SNF2/SMARC5** |
| Supt16h | 18 | 18 | 13 | 13 | | Nucleosome remodeling | | FACT |
| U2surp | 0 | 0 | 14 | 14 | | U2 snRNP-associated SURP motif-containing protein | | |
| Zfr | 9 | 9 | 16 | 19 | | Zinc-finger protein | | |
| Smarcad1 | 13 | 16 | 18 | 22 | | Transcriptional regulator - interacts with TRIM28 | | SNF2/SMARC5 |
| **Mov10** | **13** | **13** | **1** | **2** | **MOV10** | **inhibits retrotransposition** | | **RISC** |
| Parp1 | 12 | 12 | 10 | 10 | | Chromatin compaction | | |
| Eftud2 | 25 | 26 | 23 | 25 | | U5 small nuclear ribonucleoprotein | | |
| Prpf40a | 12 | 13 | 12 | 12 | | Pre-mRNA-processing | | |
| Thrap3 | 9 | 11 | 8 | 9 | | Pre-mRNA-processing | | |
| Msh2 | 8 | 8 | 12 | 12 | | Transcriptional regulator | | Smarcad1/SNF2 |
| Zfp715 | 7 | 8 | 5 | 5 | | Transcriptional regulator | | |
| Dnmt3l | 7 | 7 | 6 | 6 | | Chromatin histone binding | | |
| Wdr36 | 7 | 7 | 9 | 9 | | rRNA processing | | |
| Rbm25 | 9 | 9 | 11 | 11 | | RNA binding | | |
| Scml2 | 0 | 0 | 17 | 21 | | Regulate ubiquitination of H2A | | RNF2-RING1B |
| Sf3b2 | 8 | 8 | 20 | 21 | | Splicing factor | | |
| Ilf3 | 24 | 25 | 22 | 26 | | RNA binding | | PRMT1 |
| Ddx23 | 18 | 18 | 20 | 23 | | RNA helicase | | |
| Hells | 8 | 8 | 5 | 5 | | DNA helicase | LSH | |
| Ddx21 | 22 | 33 | 23 | 25 | | Transcriptional regulator | | SNF2/SMARC5 |
| **Kdm1a** | **13** | **14** | **3** | **3** | **LSD1/KDM1A** | **Transposon silencing** | **KDM1A** | **LSD1** |
| Wdr75 | 8 | 8 | 5 | 5 | | Transcriptional regulator | | |
| Trim71 | 9 | 9 | 5 | 5 | | RNA binding | mLin41 | |
| Sart1 | 2 | 2 | 16 | 17 | | RNA binding | | |
| Top1 | 17 | 19 | 19 | 23 | | DNA topoisomerase | | |
| Ubtf | 16 | 16 | 18 | 19 | | Transcriptional regulator of rDNA | | |
| Las1l | 6 | 7 | 15 | 15 | | Transcriptional regulator | | PRMT1 |
| Trim28 | 25 | 46 | 30 | 102 | | Transcriptional repressor | KAP1, TIF1β | TRIM28 |
| Cdc5l | 12 | 13 | 19 | 19 | | Cell division cycle 5-like protein | | |
| Tbl3 | 6 | 6 | 6 | 6 | | mRNA-processing | | |
| L1td1 | 17 | 17 | 19 | 29 | | inhibits retrotransposition | Ecat 11 | |
| Hnrnpu | 13 | 13 | 10 | 10 | | Pre-mRNA-processing | | |
| Utp14a | 1 | 1 | 13 | 13 | | U3 small nucleolar RNA-associated protein 14 | | |
| Nop2 | 14 | 14 | 12 | 12 | | RNA methyltransferase | | |
| Ddx27 | 8 | 8 | 11 | 11 | | RNA helicase | | |
| Gnl2 | 5 | 5 | 14 | 15 | | rRNA binding | | |
| Ssrp1 | 12 | 13 | 15 | 17 | | Nucleosome remodeling | | FACT |
| Mphosph10 | 8 | 8 | 8 | 8 | | Pre-rRNA-processing | | |
| Hnrnpm | 12 | 14 | 11 | 13 | | Pre-mRNA-processing | | |
| Prpf3 | 8 | 8 | 14 | 14 | | U4/U6 small nuclear ribonucleoprotein | | |
| Zfp568 | 12 | 20 | 11 | 17 | | In complex with TRIM28 | CHATO | |
| Ncl | 13 | 13 | 18 | 18 | | rRNA processing | Nucleolin | |
| Ddx18 | 7 | 7 | 12 | 12 | | RNA helicase | | |
| Gtpbp4 | 12 | 13 | 17 | 20 | | Nucleolar GTP-binding protein 1 | | |
| Kiaa0020 | 6 | 6 | 7 | 7 | | PARP1 inhibitor | Pum3 | |
| Ddx17 | 16 | 17 | 16 | 16 | | Interacts with HDAC1 | | HDAC1 |
| Ddx5 | 16 | 18 | 19 | 24 | | Transcriptional repressor, interacts with HDAC1 | | HDAC1 |
| **Fbxw8** | **12** | **12** | **3** | **3** | **FBXW8** | **Cul7-RING ubiquitin-protein ligase complex** | | **Cul7-RING** |
| Ddx52 | 6 | 6 | 5 | 5 | | RNA helicase | | |
| Dnttip2 | 6 | 6 | 7 | 7 | | Nuclear hormone receptor | ER-alpha | |
| Lmnb1 | 16 | 17 | 20 | 20 | | Lamin-B1 | | |
| Nop56 | 22 | 22 | 21 | 21 | | Ribosome biogenesis | | |
| Senp3 | 7 | 8 | 8 | 10 | | SUMO-1-specific protease 3 | | |
| Zfp472 | 7 | 7 | 4 | 4 | | Transcriptional regulator | | |
| Nop58 | 11 | 13 | 8 | 10 | | Ribosome biogenesis | | |
| Rbm39 | 8 | 9 | 8 | 9 | | Transcriptional regulator | | |
| Smu1 | 10 | 12 | 9 | 11 | | Pre-mRNA-processing | | |
| Nacc1 | 9 | 9 | 5 | 5 | | Transcriptional regulator | | |
| Prpf19 | 10 | 11 | 6 | 6 | | Ubiquitin-protein ligase | | |
| **Hdac1** | **6** | **6** | **3** | **3** | **HDAC1** | **Transcriptional repressor** | | **HDAC1** |
| D1Pas1 | 8 | 10 | 10 | 10 | | RNA helicase | | |
| Vim | 6 | 6 | 13 | 14 | | Vimentin | | |
| U2af2 | 8 | 9 | 6 | 9 | | Pre-mRNA-processing | | |
| Rest | 8 | 8 | 8 | 9 | | Transcriptional repressor | | LSD1 |
| Rsl1d1 | 11 | 11 | 11 | 11 | | Ribosomal protein | | |
| Tuba1a | 9 | 9 | 9 | 13 | | Alpha-tubulin 1 | | |
| Ddx39a | 8 | 9 | 8 | 9 | | RNA helicase | | |
| Rpl4 | 13 | 14 | 21 | 26 | | Ribosomal protein | | |
| Eif4a3 | 12 | 14 | 9 | 9 | | RNA helicase | | |
| Dek | 11 | 13 | 13 | 20 | | Chromatin DNA binding | | SNF2/SMARC5 |
| Rbmx | 7 | 8 | 8 | 10 | | RNA binding | | |
| Snrnp40 | 7 | 7 | 6 | 6 | | Pre-mRNA-processing | | |
| Gnb2l1 | 11 | 11 | 10 | 11 | | 40S ribosomal subunit | Rack1 | |
| Hnrnpc | 13 | 14 | 11 | 12 | | Pre-mRNA-processing | | |
| Fbl | 12 | 14 | 11 | 18 | | Fibrilarin | | |
| Tra2b | 8 | 12 | 7 | 7 | | RNA binding | | |
| Raly | 8 | 8 | 10 | 11 | | RNA binding | Merc | |
| Rpl7 | 17 | 19 | 20 | 29 | | Ribosomal protein | | |
| Rpl7a | 9 | 10 | 10 | 14 | | Ribosomal protein | | |
| Rps3a | 7 | 8 | 11 | 15 | | Ribosomal protein | | |
| Rps4x | 15 | 21 | 16 | 21 | | Ribosomal protein | | |
| Rps6 | 8 | 8 | 9 | 9 | | Ribosomal protein | | |
| Rpl8 | 9 | 9 | 7 | 7 | | Ribosomal protein | | |
| Srsf1 | 10 | 14 | 11 | 12 | | Ribosomal protein | | |
| Rps3 | 15 | 18 | 13 | 18 | | Ribosomal protein | | |
| Rpl13 | 7 | 8 | 7 | 9 | | Ribosomal protein | | |
| Rps15a | 7 | 7 | 8 | 8 | | Ribosomal protein | | |
| Rps9 | 12 | 12 | 14 | 16 | | Ribosomal protein | | |
| Hist1h1b | 6 | 7 | 7 | 7 | | Histone | Histone H1 | |
| Hist1h1a | 7 | 8 | 5 | 5 | | Histone | Histone H1 | |
| Rpl18 | 6 | 7 | 8 | 16 | | Ribosomal protein | | |
| Rpl23a | 9 | 9 | 14 | 17 | | Ribosomal protein | | |
| Rps13 | 8 | 8 | 8 | 8 | | Ribosomal protein | | |
| Hist1h4a | 8 | 20 | 7 | 12 | | Histone | Histone H4 | |

## Table S3: KRAB-C2H2-Zinc fingers TFs associated with TRIM28 in ES cells

| Gene Symbol | Ensembl ID | Mass spec after TRIM28 IP (peptide counts) | | | |
|---|---|---|---|---|---|
| | | WT | | D1−/− | |
| | | Unique | Total | Unique | Total |
| Zfp568 | ENSMUSG00000074221 | 12 | 14 | 11 | 13 |
| Rbak | ENSMUSG00000061898 | 11 | 11 | 11 | 12 |
| Zfp715 | ENSMUSG00000012640 | 10 | 16 | 6 | 7 |
| Zfp600 | ENSMUSG00000058186 | 10 | 32 | 11 | 33 |
| Zfp719 | ENSMUSG00000030469 | 9 | 12 | 6 | 6 |
| Znf569 | ENSMUSG00000059975 | 8 | 14 | 7 | 11 |
| Zfp101 | ENSMUSG00000055240 | 7 | 10 | 4 | 6 |
| Zfp84 | ENSMUSG00000046185 | 7 | 11 | 5 | 6 |
| Zfp869 | ENSMUSG00000054648 | 7 | 8 | 4 | 4 |
| Zfp986 | ENSMUSG00000078500 | 7 | 8 | 4 | 4 |
| AW146154 | ENSMUSG00000074166 | 7 | 7 | 4 | 4 |
| Gm15446 | ENSMUSG00000090015 | 6 | 7 | 5 | 5 |
| Zfp655 | ENSMUSG00000007812 | 6 | 8 | 6 | 9 |
| Zfp263 | ENSMUSG00000022529 | 6 | 8 | 7 | 10 |
| Zfp26 | ENSMUSG00000063108 | 5 | 5 | 2 | 2 |
| Zfp808 | ENSMUSG00000074867 | 5 | 7 | 7 | 9 |
| Zfp60 | ENSMUSG00000037640 | 5 | 7 | 7 | 8 |
| Zfp7 | ENSMUSG00000033669 | 5 | 5 | 8 | 9 |
| Zfp141 | ENSMUSG00000092416 | 5 | 5 | 1 | 1 |
| Zfp799 | ENSMUSG00000095253 | 5 | 7 | 5 | 5 |
| Zfp955a | ENSMUSG00000094441 | 5 | 6 | 6 | 6 |
| Zfp948 | ENSMUSG00000067931 | 4 | 4 | 5 | 5 |
| Zfp418 | ENSMUSG00000034538 | 4 | 5 | 2 | 2 |
| Zfp472 | ENSMUSG00000053600 | 4 | 7 | 4 | 5 |
| Zfp981 | ENSMUSG00000056300 | 4 | 6 | 6 | 14 |
| Zfp317 | ENSMUSG00000057551 | 3 | 3 | 2 | 2 |
| Zfp266 | ENSMUSG00000060510 | 3 | 3 | 0 | 0 |
| Zfp617 | ENSMUSG00000066880 | 3 | 3 | 4 | 4 |
| Zfp811 | ENSMUSG00000055202 | 3 | 3 | 0 | 0 |
| Zfp119b | ENSMUSG00000062101 | 3 | 5 | 1 | 2 |
| Znf250 | ENSMUSG00000054967 | 3 | 3 | 3 | 4 |
| Znf354c | ENSMUSG00000044807 | 3 | 3 | 3 | 3 |
| Zfp612 | ENSMUSG00000044676 | 3 | 4 | 1 | 1 |
| Zfp558 | ENSMUSG00000074500 | 3 | 6 | 3 | 6 |
| 4930522L14Rik | ENSMUSG00000072762 | 3 | 3 | 4 | 4 |
| 9830147E19Rik | ENSMUSG00000074158 | 3 | 4 | 2 | 3 |
| Zfp61 | ENSMUSG00000066880 | 3 | 3 | 4 | 4 |
| Zfp51 | ENSMUSG00000023892 | 2 | 3 | 1 | 1 |
| Zfp120 | ENSMUSG00000068134 | 2 | 3 | 0 | 0 |
| Zfp600 | ENSMUSG00000066007 | 2 | 2 | 0 | 0 |
| Zfp871 | ENSMUSG00000024298 | 2 | 2 | 5 | 6 |
| Zfp169 | ENSMUSG00000050954 | 2 | 3 | 3 | 4 |
| Zfp128 | ENSMUSG00000060397 | 2 | 2 | 3 | 3 |
| Zfp870 | ENSMUSG00000095325 | 2 | 2 | 0 | 0 |
| 5730507C01Rik | ENSMUSG00000073197 | 2 | 3 | 0 | 0 |
| Zfp989 | ENSMUSG00000086147 | 2 | 3 | 2 | 4 |
| Zfp978 | ENSMUSG00000078497 | 2 | 2 | 4 | 7 |
| Gm14443 | ENSMUSG00000078902 | 2 | 2 | 4 | 5 |
| Zfp640 | ENSMUSG00000074830 | 2 | 2 | 2 | 2 |
| Rex2 | ENSMUSG00000067919 | 2 | 10 | 2 | 8 |
| Znf12 | ENSMUSG00000029587 | 1 | 1 | 2 | 2 |
| Zfp938 | ENSMUSG00000062931 | 1 | 1 | 1 | 3 |
| Zfp819 | ENSMUSG00000055102 | 1 | 1 | 0 | 0 |
| Zfp90 | ENSMUSG00000031907 | 1 | 1 | 0 | 0 |
| Zfp37 | ENSMUSG00000028389 | 1 | 1 | 0 | 0 |
| Znf182 | ENSMUSG00000054737 | 1 | 1 | 0 | 0 |
| Zfp931 | ENSMUSG00000078861 | 1 | 1 | 0 | 0 |
| Gm13212 | ENSMUSG00000078502 | 1 | 4 | 0 | 0 |
| Zfp985 | ENSMUSG00000069727 | 1 | 1 | 1 | 1 |
| Zfp975 | ENSMUSG00000078498 | 1 | 1 | 1 | 1 |
| Zfp988 | ENSMUSG00000096916 | 1 | 1 | 0 | 0 |
| Zfp975 | ENSMUSG00000079008 | 1 | 1 | 0 | 0 |
| Zfp977 | ENSMUSG00000092335 | 1 | 1 | 0 | 0 |
| Gm17067 | ENSMUSG00000091594 | 1 | 1 | 0 | 0 |
| Zfp759 | ENSMUSG00000057396 | 1 | 1 | 1 | 1 |
| Zfp868 | ENSMUSG00000060427 | 1 | 5 | 1 | 2 |
| Zfp758 | ENSMUSG00000044501 | 1 | 2 | 1 | 2 |
| Zfp599 | ENSMUSG00000062794 | 1 | 1 | 0 | 0 |
| Zfp53 | ENSMUSG00000057409 | 1 | 1 | 1 | 1 |
| Gm14124 | ENSMUSG00000079008 | 1 | 1 | 0 | 0 |
| Zfp937 | ENSMUSG00000060336 | 1 | 1 | 0 | 0 |
| Zfp160 | ENSMUSG00000067942 | 1 | 1 | 0 | 0 |
| Zfp873 | ENSMUSG00000061371 | 1 | 1 | 1 | 1 |
| 9130019O22Rik | ENSMUSG00000030823 | 1 | 1 | 2 | 2 |
| Zfp597 | ENSMUSG00000039789 | 1 | 1 | 0 | 0 |
| Zfp712 | ENSMUSG00000090641 | 1 | 1 | 0 | 0 |
| Zfp763 | ENSMUSG00000067430 | 1 | 1 | 0 | 0 |
| Zfp820 | ENSMUSG00000069743 | 1 | 1 | 0 | 0 |
| Zfp934 | ENSMUSG00000091183 | 1 | 1 | 1 | 1 |
| Zfp949 | ENSMUSG00000032425 | 1 | 1 | 0 | 0 |
| Zfp952 | ENSMUSG00000053390 | 1 | 1 | 0 | 0 |
| Zfp97 | ENSMUSG00000095990 | 1 | 1 | 0 | 0 |
| Zfp59 | ENSMUSG00000078779 | 0 | 0 | 5 | 6 |
| Zfp57 | ENSMUSG00000036036 | 0 | 0 | 1 | 1 |
| Zfp457 | ENSMUSG00000055341 | 0 | 0 | 1 | 1 |
| Zfp985 | ENSMUSG00000065999 | 0 | 0 | 1 | 2 |
| Zfp951 | ENSMUSG00000072774 | 0 | 0 | 1 | 1 |
| AI987944 | ENSMUSG00000056383 | 0 | 0 | 2 | 2 |
| Zfp235 | ENSMUSG00000047603 | 0 | 0 | 1 | 1 |
| Zfp747 | ENSMUSG00000054381 | 0 | 0 | 1 | 1 |
| Zfp809 | ENSMUSG00000057982 | 0 | 0 | 1 | 1 |

# Table S4: Reagents and Sequences

**Primers Bisulfite sequencing**

| | Primer Name | Forward Primer | Reverse Primer | Position (Assembly NCBI37/mm9) | Primer Source |
|---|---|---|---|---|---|
| **Gnas** | Gnas-XL_BS1 | TAGTTAGTTTTTTTTGTAAGTTTTAGATTTTG | CTTAAACAACAAAAACTAACTTAACATCC | Chr 2: 174123938; 174124546 | |
| | Gnas-XL_BS2 | GGAAGTATTGGTTTTTAGAGTTTTTTT | TCCATTACTTCAAACTAAATAAAAAATCC | | |
| **Snrpn** | Snrpn_BS1 | TATGTAATATGATATAGTTTAGAAATTAG | AATAAACCCAAATCTAAAATATTTTAATC | Chr 7: 67149878; 67150301 | |
| | Snrpn_BS1 | AATTTGTGTGATGTTTGTAATTATTTGG | ATAAAATACACTTTCACTACTAAAATCC | | Hiura et al. 2006 |
| **H19** | H19_BS1 | GAGTATTTAGGAGGTATAAGAATT | ATCAAAAACTAACATAAACCCCT | Chr 7: 149767598; 149768017 | |
| | H19_BS2 | GTAAGGAGATTATGTTTATTTTTGG | CCTCATTAATCCCATAACTAT | | Tremblay et al. 199[illegible] |
| **Kcnq1ot1** | Kv-DMR_BS1 | TATAAGGAAGGTTAAGAATTTATTGTAATTATTG | AATTTCTTCTCTAAATCAACACRACACAAA | Chr 7: 150480959; 150481648 | |
| | Kv-DMR_BS2 | GTTTTTATTTTAAGTTTTTAATTTTTTATATTTGAATTT | ACAAACACTCACRACCAAAAACAAAAATAAT | | |
| **Gtl2** | IG-DMR_BS1 | AGATGTGTTGTGGATTTAGGTTGTAG | CTAAACTACAATCTATATAATCACAACAC | Chr 12: 110766408; 110766806 | |
| | IG-DMR_BS2 | AAGTGTTGTGGTTTGTTATGGGTAAG | CCATTCCCAATCTATAAAAATATTTTAACC | | |

**Primers Northern-blot probe**

| | Primer Name | Forward Primer | Reverse Primer |
|---|---|---|---|
| **GAPDH** | GAPDH | GCACAGTCAAGGCCGAGAATGG | TGGGGGCCGAGTTGGGATAGG |

**Primers ChIP**

| | Primer Name | Forward Primer | Reverse Primer | Position* | Primer Source |
|---|---|---|---|---|---|
| **Negative** | TRIM28_negative_ChIP | CTAGTCTCCTGTGCGTATGTGG | CTCAAAGAGAGCCAGCCATTAG | Chr 5: 135766812; 135767045 | |
| **IAP U3** | U3_ChIP | CGAGGGTGGTTCTCTACTCCAT | GACGTGTCACTCCCTGATTGG | 5' LTR:87 - 174 ; 3 'LTR : 6817 - 6904 | Rowe et al. 2010 |
| **IAP 5' LTR** | 5'LTR_ChIP | TGGGGAACGAGAGTACCAGT | GGAACAAAAGGGCTTCTAACAC | 509 - 653 | |
| **IAP pol** | pol_ChIP | TGTGCCCCAAAAGGTAGAGATA | CCTTAACTCAGCGGAAGGAAT | 3755 - 3875 | |

* reference IAP sequence RP23-92L23

**Sequence of the sgRNAs targeting the U3 region of the IAP transposons LTRs**

| sgRNA | Sequence | Position* | |
|---|---|---|---|
| | | 5' LTR | 3' LTR |
| sgRNA_IAP_U3_#1 | CCATCTTGTAACGGCGAATG | 19 - 38 | 6749 - 6768 |
| sgRNA_IAP_U3_#2 | ATAATCTGCGCATGTGCCGA | 70 - 89 | 6800 - 6819 |
| sgRNA_IAP_U3_#3 | GTGACGTCAACTCGGCCGAT | 125 - 144 | 6855 - 6874 |
| sgRNA_IAP_U3_#4 | AATTCTCTTTAATAGGGACG | 188 - 207 | 6918 - 6937 |

* reference IAP sequence RP23-92L23

| Antibody | Source | Identifier | Application |
|---|---|---|---|
| TRIM28 | EMD Millipore | MAB3662 | IP - ChIP |
| O-GlcNAc | Thermo Fisher Scientific | HGAC85 | ChIP-seq |
| TRIM28 | Abcam | ab10483 | WB |
| OGT | Santa Cruz | SC-32921 | WB |
| O-GlcNAc | Santa Cruz | SC-59623 | WB |
| TUBULIN | Sigma | T6199 | WB |
| FLAG | Sigma | F3165 | WB |
| DNMT1 | Santa Cruz | SC-20701 | WB |
| DNMT3A | Bethyl Laboratories | A-304-278A | WB |
| DNMT3B | Novus | 52A1018 | WB |
| CHD3 | Cell Signaling | 4241 | WB |
| CHD4 | Sigma | SAB4200107 | WB |
| BRG1 | Cell Signaling | D1Q7F | WB |
| ZNF198 | Bethyl Laboratories | A301-711A | WB |
| SMARCA5 | EMD Millipore | ABE1026 | WB |
| SF3B1 | Thermo Fisher Scientific | PA5-19679 | WB |
| SIN3A | EMD Millipore | MABE607 | WB |
| SETDB1 | Proteintech | 11231-1-AP | WB |
| G9A | Cell Signaling | 3306 | WB |
| PARP1 | Cell Signaling | 9532 | WB |
| LSD1 | Abcam | Ab17721 | WB |
| CTCF | Cell Signaling | 3418 | WB |
| SUV39H1 | Cell Signaling | 8729 | WB |
| HDAC2 | Cell Signaling | 5113 | WB |
| HDAC1 | Cell Signaling | 5356 | WB |
| HP1α | EMD Millipore | 05-689 | WB |
| Histone H3.3 | EMD Millipore | 09-838 | WB |
| Histone H2A.Z | Gift from Stefan Dimitrov | | WB |
| Histone H2A.X | Cell Signaling | 7631 | WB |
| Histone H3 | Abcam | Ab1791 | WB |
| Histone H2A | EMD Millipore | ABE327 | WB |
| Histone H2B | EMD Millipore | 07-371 | WB |
| Histone H4 | Thermo Fisher Scientific | MA5-14816 | WB |
| NANOG | Abcam | Ab80892 | WB |

**Bisulfite sequencing: SNPs positions**

| ICR | SNP Position (Assembly NCBI37/mm9) | Bl6 | CAST |
|---|---|---|---|
| **Gnas** | Chr 2:174,123,975 | G | A |
| | Chr 2:174,124,504 | G | A |
| **Snrpn** | Chr 7:67,150,273 | G | A |
| | Chr 7:67,150,263 | T | A |
| | Chr 7:67,150,203 | T | G |
| | Chr 7:67,150,193 | TTT | **A |
| | Chr 7:67,150,183 | G | A |
| | Chr 7:67,150,103 | G | T |
| **H19** | Chr 7:149,767,840 | G | * |
| | Chr 7:149,767,759 | G | A |
| | Chr 7:149,767,672 | A | G |
| **Kv-DMR** | Chr 7:150,481,047 | A | G |
| | Chr 7:150,481,242 | GGA | AGC |
| **IG-DMR** | Chr 12:110,766,358 | A | G |
| | Chr 12:110,766,469 | G | A |
| | Chr 12:110,766,665 | G | A |

* deletion

## SUPPLEMENTARY FIGURES

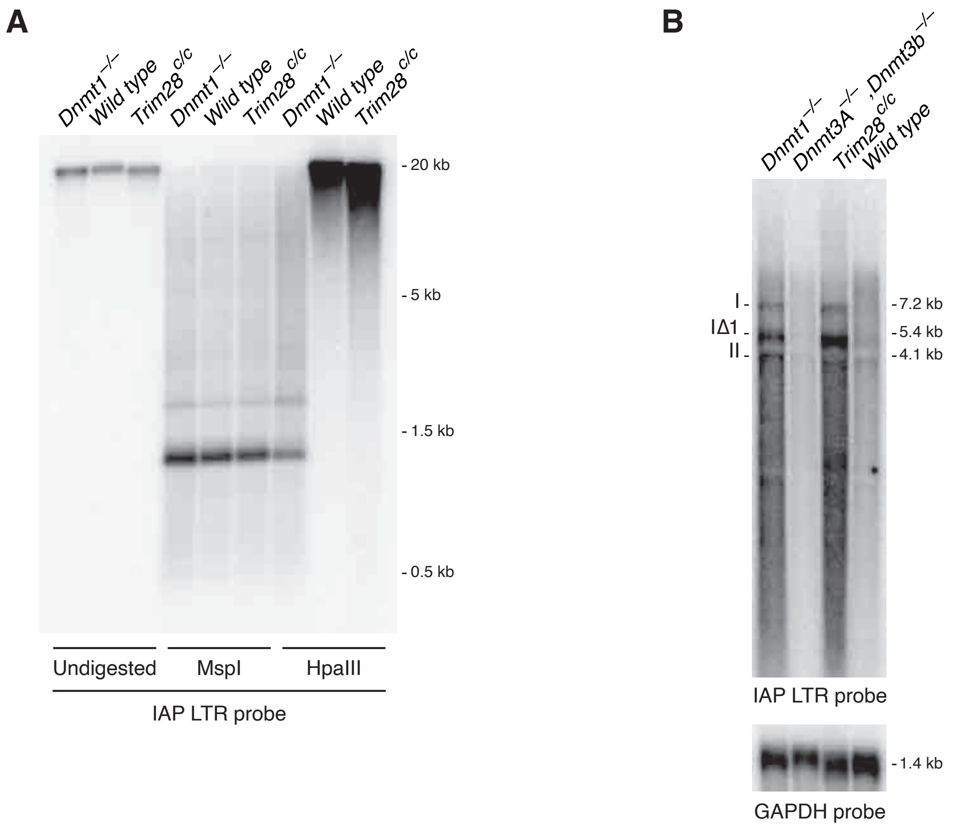


**Fig. S1. The protein TRIM28 is necessary to repress transcription of methylated retrotransposons.** The *Chatwo* or *C* allele of *Trim28* was ENU-induced and bears two adjacent non-conservative amino acid substitutions and has a similar but slightly less severe phenotype than the null allele (1, 2). (A) Methyl-sensitive southern-blot showing dense cytosine methylation at IAP (intracisternal A-type particles) LTR (long terminal repeats) in embryos bearing the homozygous mutation *Chatwo* ($Trim28^{C}$). Genomic DNA extracted from wild type embryos is digested by the methylation-insensitive restriction enzyme MspI, but is not digested by its methylation sensitive isoschizomer HpaII. Genomic DNA isolated from $Dnmt1^{-/-}$ ES cells is fully digested by HpaII, reflecting global DNA demethylation. In contrast, DNA extracted from $Trim28^{C/C}$ embryos is refractory to cleavage by HpaII, showing that IAP LTRs are densely methylated in $Trim28^{C/C}$ embryos. (B) Transcriptional activation of IAP retrotransposons in $Trim28^{C/C}$ embryos. RNA blot hybridized with IAP LTR probes showing that IAPs are expressed at the same high levels in $Trim28^{C/C}$ embryos and $Dnmt1^{-/-}$ cells; the spectrum of IAP subtypes reactivated is very similar. In contrast, IAP retrotransposons are repressed in wild type littermate embryos and early passage $Dnmt3a^{-/-}$, $Dnmt3b^{-/-}$ ES cells (normally methylated at IAP). Expression of methylated IAP retrotranspons in $Trim28^{C/C}$ embryos indicates that DNA methylation requires TRIM28 protein to repress transcription.

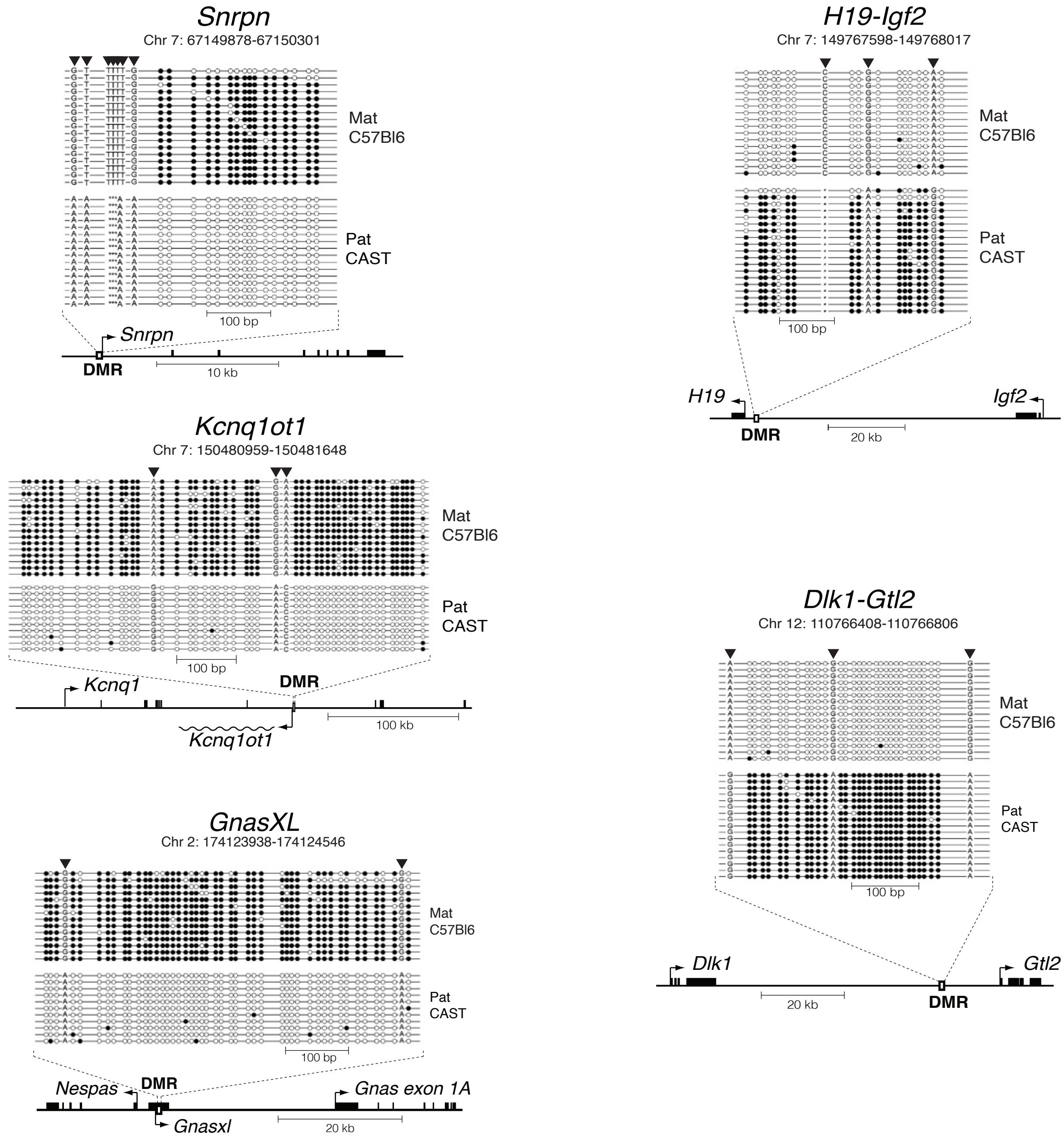


**Fig. S2. Loss of imprinted expression in *Trim28<sup>C/C</sup>* embryos occurs despite normal methylation at differentialy methylated regions (DMRs)**. Normal mono-allelic methylation at imprinting control regions DMRs in *Trim28$^{C}$* homozygous embryos that show bi-allelic expression of imprinted genes and derepression of methylated IAP retrotransposon. The DMRs of the *H19-Igf2* and *Dlk1-Gtl2* reciprocally imprinted gene pairs are paternally (Pat) methylated; *Snrpn*, *Kcnq1ot1* and *GnasXL* are maternally (Mat) methylated. The methylation status of each CpG site within the five analyzed DMRs was determined by bisulfite sequencing from single *Trim28$^{C/C}$* embryos. The parent of origin of the alleles was determined using single nucleotide polymorphisms in C57Bl6J x M. m. castaneus (CAST) F1 hybrids as indicated by arrows. Open and filled circles respectively represent unmethylated and methylated CpG sites. The previously reported bi-allelic expression of *H19*, *Gtl2*, *Snrpn*, *Kcnq1ot1* and *GnasXL* in *Trim28$^{C/C}$* homozygous embryos (2) indicates that TRIM28 protein is crucial for the monoallelic expression of imprinted genes.

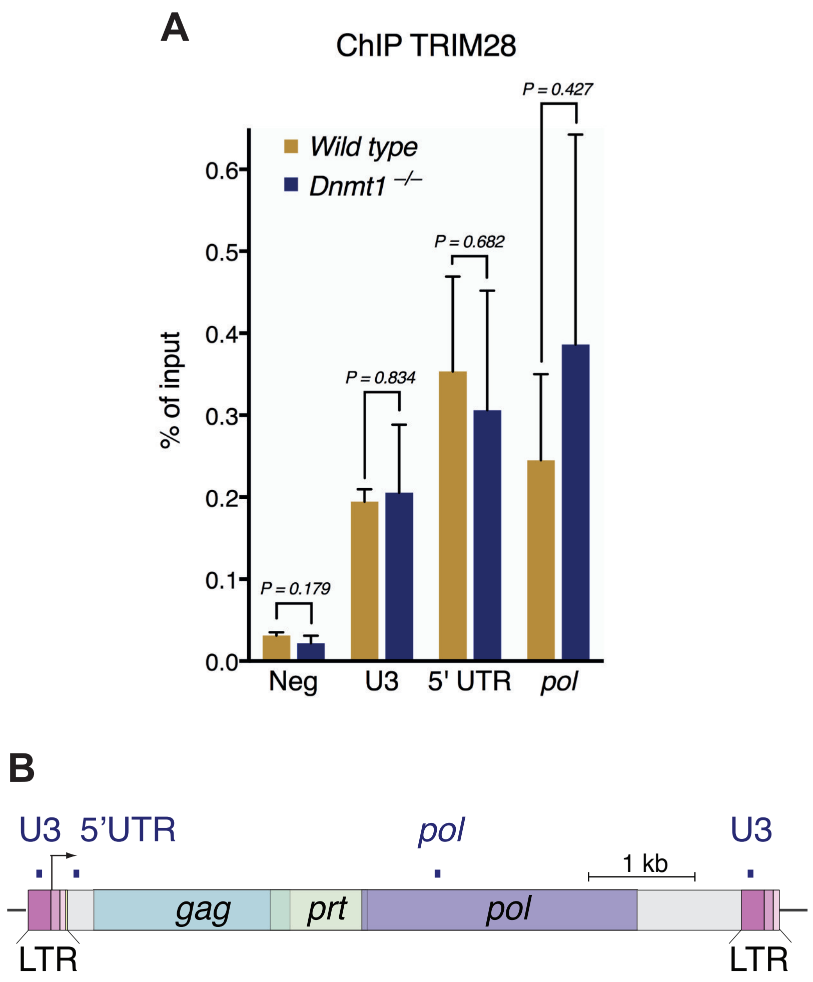


**Fig. S3. Demethylation does not cause dissociation of TRIM28 from IAP retrotransposon sequences.** (A) ChIP-qPCR showing high occupancy of TRIM28 at IAP LTRs in both wild type (normally methylated) and *Dnmt1*$^{-/-}$ ES cells (demethylated). The binding of TRIM28 to unmethylated IAP sequences indicates that its recruitment to retrotransposons is independent of DNA methylation (see Fig. S7). This is comparable to the case in Drosophila where *Hox* genes are derepressed in the absence of OGT/Scx despite normal binding of Polycomb factors (3). (B) The positions of the primer sets used for real-time PCR quantification of DNA binding are indicated as black squares on the schematic representation of an IAP element. Sequence coordinates are in Table S4. Results are presented as a relative enrichment as compared to an arbitrarily chosen genomic locus (Chr5: 135766812-135767045 in mm10). Bar represents means and standard deviations of three biological replicates. P values were calculated using an unpaired *t*-test.

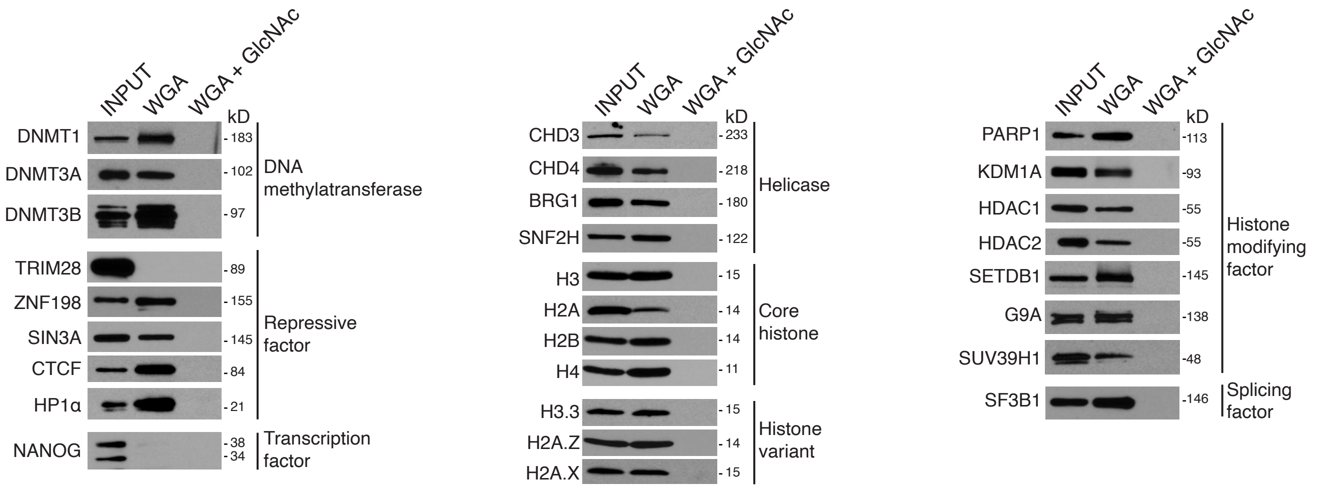


**Fig. S4. *O*-GlcNAcylation of proteins associated with TRIM28.** Nearly all chromatin-associated proteins tested were *O*-GlcNAcylated in wild type ES cells, as assessed by binding to the *O*-GlcNAc specific lectin wheat germ agglutinin (WGA); a notable exception is TRIM28 itself. NANOG had been previously reported to lack *O*-GlcNAclyation (4), as confirmed here. Specificity of binding of WGA to *O*-GlcNAcylated proteins was evaluated by measuring inhibition of binding by free N-Acetylglucosamine (GlcNAc). Gel loading from left to right, first lane: input, second lane: WGA purification, third lane: WGA purification in presence of free GlcNAc.

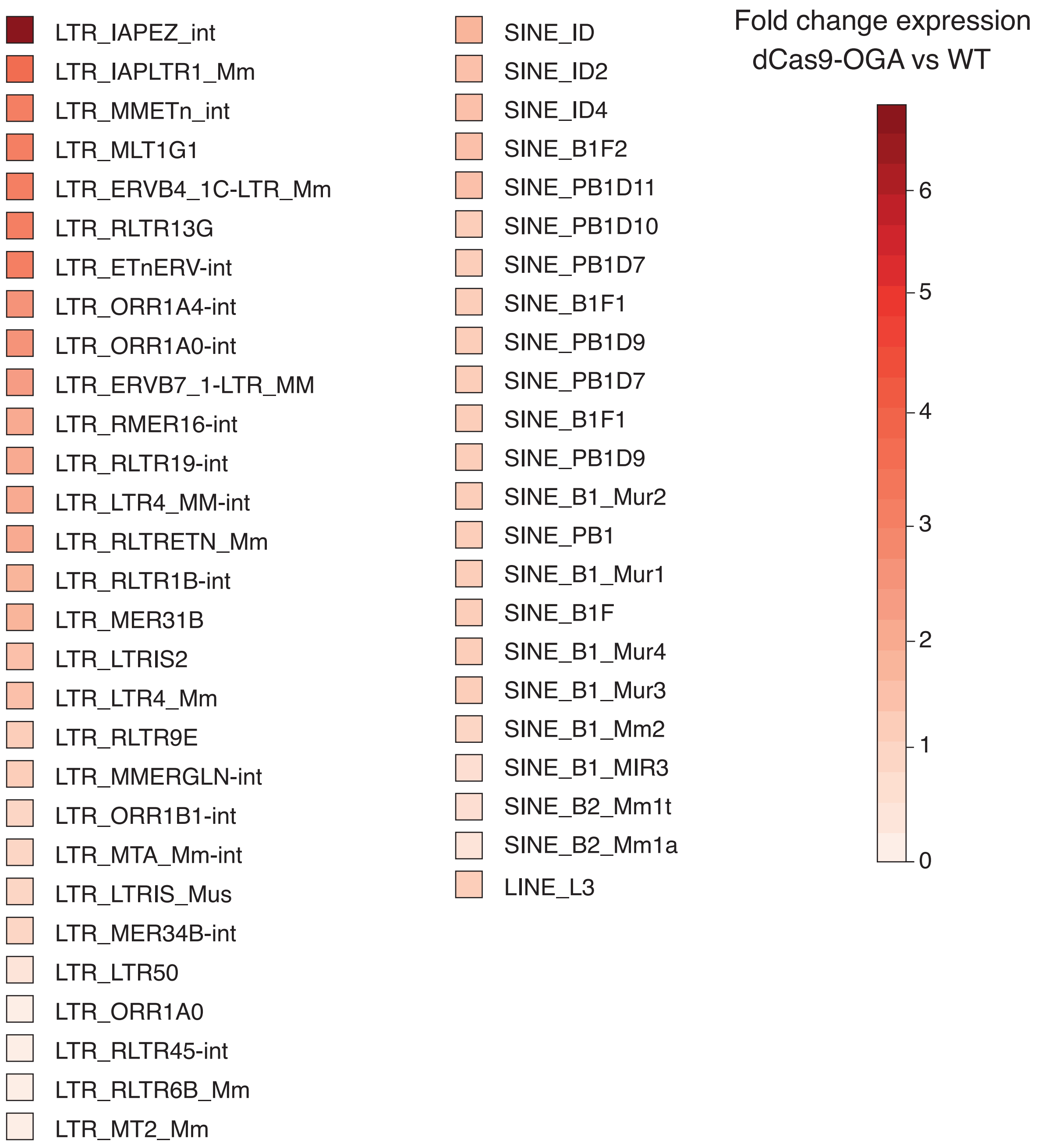


**Fig. S5. Specific reactivation of IAPEz transposons upon targeting of dCas9-OGA to their LTRs.** Heat map showing the expression fold change of all families of transposons in ES cells expressing dCas9-OGA and four sgRNA against IAPEz LTRs. Transposons families with fold change equal to zero are not shown. IAPEz is the only type of transposons that substan-tially reactivates after targeted de-GlcNAcylation of proteins bound to their LTRs.

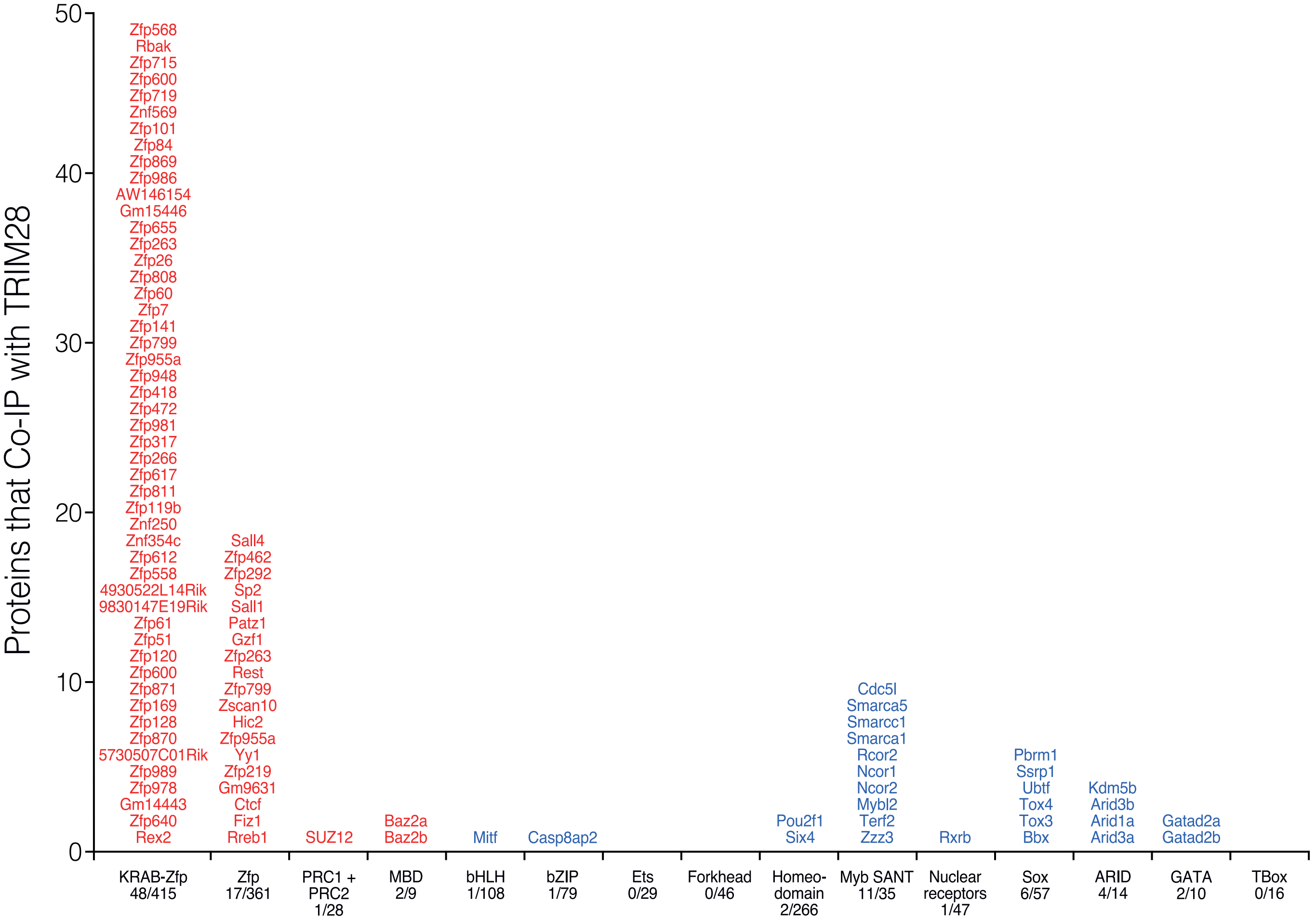


**Fig. S6. Predominance of proteins with KRAB and Zinc finger domains among the DNA binding proteins found to Co-IP with TRIM28 by mass spectrometry.** TRIM28 primarily associates with KRAB and Zinc finger domains proteins. Above each category are the names of the TRIM28-associated transcription factors.

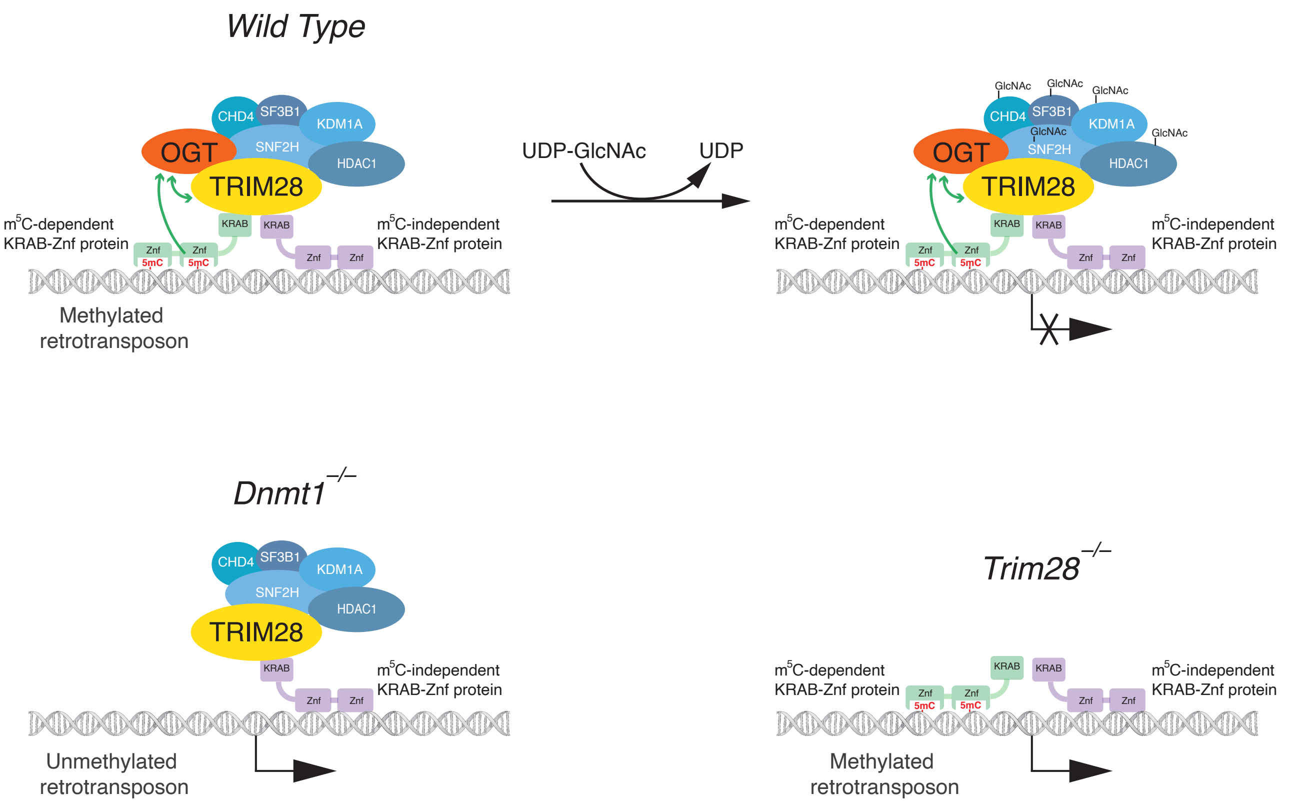


**Fig. S7. Model.** Top: Schematic representation of the interactions between methylated LTRs and the TRIM28-OGT repressive complex. TRIM28 and local O-GlcNAcylation are both required for stable silencing of methylated retrotransposons. OGT catalyzes the linkage of *O*-GlcNAc on serine and threonine residues of several repressors complexed with TRIM28/OGT, including CHD4, SF3B1, KDM1A, SNF2H and HDAC1. The binding of TRIM28 to retrotransposons' LTRs occurs independently of their methylation state while OGT's recruitment to the TRIM28 complex and subsequent O-GlcNAcylation of associated proteins depend both on cytosine methylation. The identity of the transcription factor(s) that recruit TRIM28 and OGT to methylated transposons is unknown. TRIM28 is complexed with a plethora of KRAB-Znfs, the largest family of transcription factors. Most of KRAB-Znfs have unknown binding motif but structure studies showed that ~1/3 have features compatible with recognition of 5-methyl cytosines (5). Based on this evidence, we propose that one or several methylation-independent KRAB-Znfs recruit TRIM28 while other(s) methylation-dependent KRAB-Znfs are responsible for OGT recruitment to the repressive complex. Note that the protein interactions depicted in the scheme are not necessarily direct. Bottom: Schematic representation of possible disruptions of the repressive complex in *Dnmt1*$^{-/-}$ and *Trim28*$^{-/-}$ cells. Both mutations cause a pervasive reactivation of transposons and bi-allelic expression of imprinted genes. Local de-*O*-GlcNAcylation of chromatin at LTRs phenocopies *Trim28*$^{-/-}$: retrotransposons undergo release from silencing without loss of DNA methylation.

## SUPPLEMENTARY METHODS

*DNA methylation analysis by methylation-sensitive restriction enzyme*

Genomic DNA was isolated from a pool of three E8.5 dpc *Trim28*$^{C/C}$ embryos or from 1 x $10^6$ ES cells and quantified using Qubit Fluorometric Quantitation (Thermo Fisher Scientific). Genomic DNA was digested with either the methylation-sensitive endonuclease HpaII or its methylation insensitive isoschizomer MspI (New England Biolabs). The southern blot protocol and the probes were previously described [6]. The ENU-induced *Trim28*$^{C}$ mutation was previously described [1,2].

*DNA methylation analysis by bisulfite sequencing*

Genomic DNA extracted form a single E8.5 embryo homozygous for *Trim28*$^{C}$ and heterozygous for C57Bl6 and M.m castaneus SNPs at imprinting control regions (ICRs) was bisulfite converted using the EZ DNA Methylation Kit (Zymo Research). ICRs were amplified with 2 rounds of nested PCR (primers sequences SI Appendix table S4). The PCR product resulting from the second round of PCR was cloned into the pJET vector (Thermo Fisher Scientific). Approximately 40 positive clones for each ICR were sequenced and all sequences had a conversion rate >95%. The parental origin of each ICR was determined on the basis of its strain-specific SNP content according to http://www.sanger.ac.uk/science/data/mousegenomes-project. Sequences were analyzed with Quma online software [7].

*Chromatin Immunoprecipitation followed by QPCR*

ChIP-QPCR of TRIM28 was performed on formaldehyde cross-linked chromatin. $10^8$ ES cells were fixed for 10 min at room temperature with 1.1% formaldehyde and quenched with 125 mM glycine. Soluble chromatin isolated as previously described [8] was sheared by sonication to an average size of 500-1,000 bp (Branson sonicator). The chromatin was then diluted to a concentration of 1 mg/ml. Immunoprecipitation was carried out overnight at 4°C with 3 µg of monoclonal antibody to TRIM28 (EMD Millipore, MAB3662) bound to 10 µl Dynabeads conjugated with protein G (Life Technologies). Beads were washed and chromatin eluted as described previously [8]. Enrichment compared to input was analyzed by qPCR using the primers listed in SI Appendix Table. S4. The experiment was performed in biological triplicate and p-values were calculated with the unpaired *t*-test.

## SUPPLEMENTAL REFERENCES


1. M. Shibata, K. E. Blauvelt, K. F. Liem, M. J. García-García, TRIM28 is required by the mouse KRAB domain protein ZFP568 to control convergent extension and morphogenesis of extra-embryonic tissues. *Development* **138**, 5333 5343 (2011).

2. K. A. Alexander, X. Wang, M. Shibata, A. G. Clark, M. J. García-García, TRIM28 Controls Genomic Imprinting through Distinct Mechanisms during and after Early Genome- wide Reprogramming. *Cell Reports* **13**, 1194 1205 (2015).

3. M. C. Gambetta, K. Oktaba, J. Müller, Essential role of the glycosyltransferase sxc/Ogt in polycomb repression. *Science* **325**, 93 96 (2009).

4. H. Jang, *et al.*, O-GlcNAc Regulates Pluripotency and Reprogramming by Directly Acting on Core Components of the Pluripotency Network. *Cell Stem Cell* **11**, 62 74 (2012).

5. Y. Liu, X. Zhang, R. M. Blumenthal, X. Cheng, A common mode of recognition for methylated CpG. *Trends Biochem Sci* **38**, 177 183 (2013).

6. S. K. Ooi, *et al.*, Dynamic instability of genomic methylation patterns in pluripotent stem cells. *Epigenet Chromatin* **3**, 17 10 (2010).

7. Y. Kumaki, M. Oda, M. Okano, QUMA: quantification tool for methylation analysis. *Nucleic Acids Res* **36**, W170 W175 (2008).

8. M. Boulard, J. R. Edwards, T. H. Bestor, FBXL10 protects Polycomb-bound genes from hypermethylation. *Nat Genet* **47**, 479 485 (2015).
